# Characterizing protein sequence determinants of nuclear condensates by high-throughput pooled imaging with CondenSeq

**DOI:** 10.1038/s41592-025-02726-y

**Published:** 2025-06-16

**Authors:** Kalli Kappel, Daniel Strebinger, KeHuan K. Edmonds, Samuel Chau-Duy-Tam VO, Christopher M. Vockley, Tridib Biswas, Samouil L. Farhi, Rhiannon Macrae, Feng Zhang, Aviv Regev

**Affiliations:** 1 Howard Hughes Medical Institute, Cambridge, MA 02139, USA; 2 Broad Institute of MIT and Harvard, Cambridge, MA 02142, USA; 3 McGovern Institute for Brain Research, Massachusetts Institute of Technology, Cambridge, MA 02139, USA; 4 Department of Brain and Cognitive Science, Massachusetts Institute of Technology, Cambridge, MA 02139, USA; 5 Department of Biological Engineering, Massachusetts Institute of Technology, Cambridge, MA 02139, USA; 6 Spatial Technology Platform, Broad Institute of MIT and Harvard, Cambridge, MA 02142, USA; 7 Current address: Genentech, 1 DNA Way, South San Francisco, CA, USA

## Abstract

Biomolecular condensates — nonstoichiometric, membrane-less assemblies — organize numerous subcellular processes and are implicated in diseases, including neurodegeneration and cancer. Protein sequences intrinsically encode their propensity to form condensates, but specific sequence features that regulate this behavior have not been systematically explored at scale. Here, we develop and apply CondenSeq, a high-throughput pooled imaging with *in situ* sequencing approach to measure propensities of thousands of protein sequences to form nuclear condensates. Leveraging the large scale of these experiments, we evaluated the impacts of dozens of sequence features across a wide range of sequence contexts, identifying several with highly consistent, context-independent effects, and others with less consistent effects. We also identified multiple classes of condensates and discovered distinct sequence properties that drive their formation. Our results provide a systematic overview of the relationships between protein sequences and nuclear condensate formation and establish a general approach for further dissecting these relationships at scale.

## INTRODUCTION

Biomolecular condensates, membrane-less subcellular assemblies of proteins and/or nucleic acids that selectively concentrate specific biomolecules, are widespread in cell biology^1, 2^. They include membrane-less organelles, such as nucleoli and stress granules; we use the term condensate here to refer generally to concentrated foci that lack an encapsulating membrane. Condensates are implicated in processes such as gene regulation, RNA processing, and signaling^3^, and a growing body of evidence implicates aberrant condensates in human disease, including neurodegeneration and cancer^4–6^. Condensates form through diverse mechanisms and have different material properties^1, 2, 7–9^. Many condensates are driven to form through interactions between intrinsically disordered protein regions (IDRs)^10–12^. However, not all IDRs form condensates.

Deciphering the protein sequence features that govern condensate formation in cells would transform our understanding of this fundamental form of subcellular organization. Several landmark studies of model systems have begun to uncover protein sequence features within IDRs that are critical for condensate formation, by mutational analysis of up to tens of protein sequence variants^13–25^. These studies, together with a large body of theoretical work^26–35^, have revealed that in different contexts nearly every amino acid type (aromatic, hydrophobic, polar, positively charged, negatively charged, etc.) and the patterning of those amino acids can affect condensate formation. Yet, many questions remain open. First, how consistent are the impacts of these sequence features across diverse sequence contexts? Because each study typically focused on a single model protein or family of proteins, it is unclear if the impactful features discovered in one system would generalize to others, or what the relative effects of impactful features discovered in different systems might be. Second, are there other important sequence features that have yet to be discovered? As new model systems have continued to be studied, new sequence features have been revealed, suggesting that a one-by-one approach may not scale to the full scope of protein sequence space. Finally, how can we compare conclusions from studies performed in different experimental conditions? Condensate formation is concentration dependent and sensitive to solution and environmental conditions^2, 36–38^. A protein will not necessarily have the same propensity to form condensates *in vitro* versus in cells^38^, across different cell types, or when localized, even in the same cell type, to the nucleus versus the cytoplasm^37, 39^. This environmental sensitivity makes it difficult to compare conclusions across studies.

Addressing these questions requires an assay that can test large numbers of protein sequence variants in consistent and biologically-relevant experimental conditions. Existing methods to study the propensity of a protein of interest to form condensates fall into three broad categories: computational, *in vitro*, and cellular approaches^40^. Many computational approaches have been developed to predict the propensity of a protein sequence to form condensates^41–48^. While significant progress has been made, these methods are not yet robust enough to obviate the need for experiments, and can benefit from larger training datasets. *In vitro* approaches report on the intrinsic biophysical properties of the protein^38, 49, 50^. While they have yielded critical biophysical information, they do not necessarily suffice to determine a protein’s behavior in a cell^38^, where a protein can make heterogeneous interactions with other cellular factors^38^ (Supplementary Figure 1A). Fluorescent imaging can measure the propensity of a protein to form condensates directly in cells^40^. However, because existing fluorescent imaging approaches have been performed in an arrayed format, with one protein sequence variant per well, it has been challenging to scale to more than tens of sequence variants. Other experimental techniques characterize other properties of condensates in cells, beyond just the propensity of a sequence to form condensates, including mass spectrometry-based approaches to measure condensate components, cryo-electron tomography to reveal the detailed three-dimensional architecture of a condensate, and FRAP and microrheology to measure diffusion of molecules in a condensate^38, 40, 51^ (Supplementary Table 1). These approaches are even more challenging to scale to large numbers of protein variants. Thus, given the enormity of protein sequence space and that even the dozens of sequence features proposed to drive condensate formation have yet to be tested across a wide range of sequence contexts in consistent, biologically-relevant conditions, there remains a critical need for an experimental approach that can readily scale to hundreds to thousands of protein sequence variants.

Here, to address these challenges and systematically discover, compare, and assess the protein sequence features that drive condensate formation in cells, we developed CondenSeq, which combines high-throughput pooled imaging of fluorescently tagged synthetic protein sequences, a previously developed approach for *in situ* barcode sequencing^52, 53^, and computational analysis to characterize the propensities of thousands of protein sequences to form condensates in the nucleus. We applied CondenSeq to libraries of approximately 14,000 short synthetic protein sequences, designed to assess the impacts of amino acid composition and patterning across diverse sequence contexts. We focused on condensate formation in the biologically relevant cellular environment, within the nucleus, a compartment in which condensates spatially organize numerous processes^9^. Using images of approximately 7.8 million cells, we identified multiple classes of nuclear condensates and learned the sequence features that distinguish the proteins in each class. Our large-scale, highly controlled dataset, enabled by the unique efficiency and scalability of CondenSeq, elucidated the relative impacts of different protein sequence features on nuclear condensate formation and, surprisingly, revealed features that consistently promoted condensate formation, largely independent of sequence context, alongside more context-specific ones.

## RESULTS

### Pooled image-based characterization of condensates

**Fig. 1.**
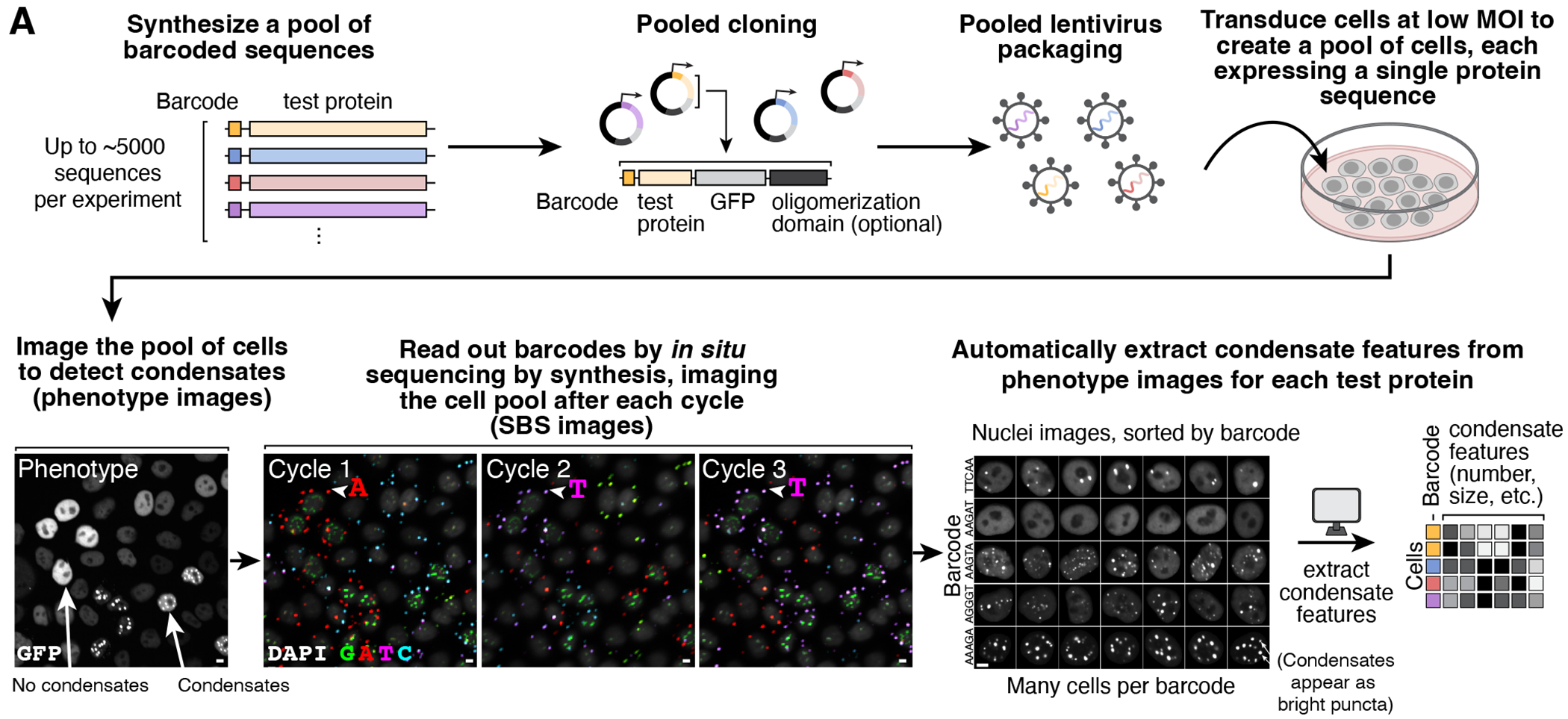
CondenSeq: Pooled image-based characterization of condensates. (A) CondenSeq overview. A DNA oligo pool, with each oligo encoding a unique barcode (BC) and a test protein, is synthesized, cloned as a fusion to a fluorescent protein (GFP or SNAP-tag), packaged into lentiviral particles, and delivered at low MOI to cells (HeLa, unless otherwise specified). The cells are imaged with a confocal microscope in the Hoechst and GFP/SNAP-tag channels to detect nuclei and condensates, respectively (phenotype imaging). An example phenotype image is shown with arrows that point out example cells with and without condensates. The cells are then fixed and barcodes are detected through *in situ* sequencing by synthesis (SBS). Example SBS images corresponding to the example phenotype image are shown. Arrows denote example base calls for one cell over the three cycles. Phenotype images are then mapped to the barcodes and condensate features are computed with an automated computational workflow. The final output is a matrix of cells, with associated barcodes and condensate features. See Supplementary Table 1 for a detailed comparison between CondenSeq and other approaches for measuring the propensities of protein sequences to form condensates in cells. Scale bars denote 5 μm.

To systematically dissect the protein sequence features impacting nuclear condensate formation, we developed CondenSeq to efficiently assay thousands of protein sequences in the controlled cellular context of the nucleus (Fig. 1A). A CondenSeq assay visualizes condensate formation by imaging a pool of cells, each expressing one fluorescently tagged protein sequence. *In situ* sequencing is then used to link cells to the specific protein sequences they express. Finally, automated image analysis extracts and quantifies condensate features in each cell. Unlike other approaches for measuring propensities of protein variants to form condensates in cells, CondenSeq is inherently scalable (Supplementary Table 1). Here, we assayed up to ~5,000 protein sequence variants per sample, whereas other approaches measure only one sequence per sample. Additionally, CondenSeq measures condensates in live cells to avoid fixation artifacts^54^; CondenSeq measures condensate morphology; and, CondenSeq captures cell-to-cell variability as all measurements are made in single cells. CondenSeq also has limitations (see Discussion). Importantly, it does not reveal specific interactions that a test protein may make with endogenous cellular factors.

A CondenSeq experiment begins with a DNA oligonucleotide pool, with each oligonucleotide encoding one protein sequence and a short DNA barcode (5 or 8 nucleotides here) (Fig. 1A). We clone this pooled library into a lentiviral expression vector as a fusion to (Supplementary Figure 1B): (**1**) a fluorescent protein for visualization (here, GFP or SNAP-tag), (**2**) a nuclear localization signal, so all protein sequences are characterized in a single defined cellular compartment, the nucleus, and (**3**) optionally, an oligomerization domain, which enabled manipulation of protein valence, a parameter known to impact condensate formation (Supplementary Figure 1C); by measuring libraries fused to different oligomerization domains (*e.g.,* 4-mer versus 24-mer), we can determine the effect of valence. We transduce HeLa cells (unless otherwise specified) with the library in a pooled format, and image live cells in both the fluorescent protein and Hoechst channels, to visualize the test protein and nucleus, respectively (“phenotype images”, Fig. 1A). Condensates appear as bright puncta in the fluorescent protein channel. This approach does not provide information about material properties of the condensates. To assign cell/nucleus images to individual protein sequences, we fix the cells and read out the DNA barcodes directly in cells through *in situ* sequencing by synthesis^53^ (SBS) (Fig. 1A). Finally, we automatically detect condensates in the phenotype images and computationally extract their visual features, along with total nuclear protein concentration (Fig. 1A, Supplementary Figure 1D–E, Methods, Supplementary Note 1). On average, CondenSeq yielded data for 93,735 cells per well of a 24-well plate. Control experiments confirmed that Hoechst staining did not impact condensates (Supplementary Figure 1F).

### CondenSeq recapitulates known sequence-dependent trends

To test whether CondenSeq recapitulates previously reported sequence-dependent trends, and to assess its technical accuracy and reproducibility, we performed live-cell time-lapse imaging in HeLa cells over ~28 hours for a library of 99 protein sequences, each 66 amino acids long (“small sequence library”, Supplementary Data 1). This library included disordered fragments of proteins previously shown to drive condensate formation and variants designed to test previously reported sequence-dependent trends^14, 15, 55–58^ (Supplementary Note 1, Supplementary Figure 2A). All proteins were 66 amino acids long, but we also performed separate proof-of-principle tests demonstrating that CondenSeq can be applied to longer protein sequences (Supplementary Results, Supplementary Data 2, Supplementary Figure 3).

In an initial test of this small sequence library fused to GFP without an oligomerization domain imaged at a single timepoint, only 3.7% of protein sequences formed condensates in more than 30% of cells. To improve the dynamic range of our measurements and our ability to observe condensate formation for a larger fraction of this library of short protein fragments within the protein concentration range accessible in a cell, we fused it to an oligomerization domain, FTH1, which forms 24-mers, and has previously been used to manipulate protein valence in condensate formation assays^59–61^. Indeed, with this oligomerization domain, 61.4% of the protein sequences formed condensates in >30% of cells (Supplementary Figure 4A). Without a fused test protein, the oligomerization domain did not form condensates (Supplementary Figure 4B) and endogenous FTH1 levels in HeLa nuclei were low compared to the GFP library expression level (Supplementary Figure 4C).

**Extended Data Fig. 1:**
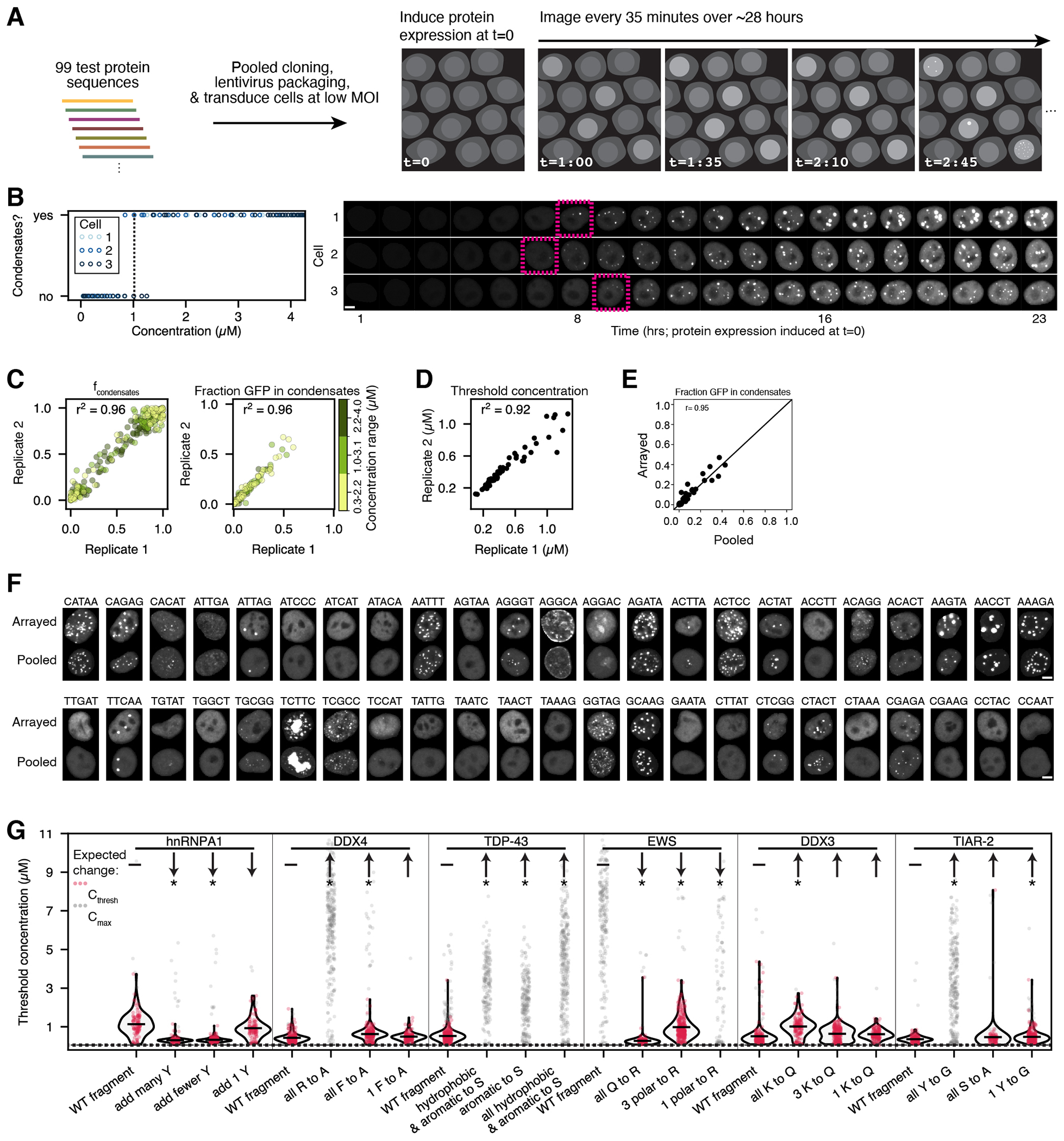
Live cell timelapse experiments. (**A**) Schematic overview of the live cell timelapse experiments. (**B**) Example traces from live cell timelapse imaging (barcode: TCGGG). Left: The presence of condensates in three example cells as a function of total protein concentration. The dashed black line denotes the average threshold concentration (C_thresh_), the concentration at which condensates are first observed, for these cells. Right: Images of the same three cells (nuclei, masked) over time. Dashed pink boxes denote the first frame in which condensates appear. Two independent biological replicates of this experiment were performed with the same results. (**C**) Reproducibility between replicates for live cell timelapse experiments. For the fraction of cells with condensates (*f*_*condensates*_; left), each point represents *f*_*condensates*_ for one protein sequence within a defined concentration bin. For the fraction of GFP in condensates, each point represents the mean value of the fraction of the GFP signal in condensates over all cells that express a particular protein sequence within a defined concentration bin. (**D**) Reproducibility of threshold concentrations determined from independent replicates of live cell timelapse experiments. Each point represents the threshold concentration for a single protein sequence. (**E**) The fraction of the total GFP signal found in condensates for the pooled versus arrayed experiments. Each point represents the mean value of the fraction of the GFP signal in condensates over all cells that express a particular protein sequence within the medium concentration bin. Pearson’s correlation is noted on the plot. (**F**) Representative images of nuclei (masked) from arrayed and pooled experiments (all sequences were fused to the 24-mer oligomerization domain and GFP). Barcodes are indicated on the left of each pair of images. Scale bar = 5 μm. Two independent biological replicates of this experiment were performed with the same results. (**G**) C_thresh_ for protein sequences corresponding to previously studied constructs. Each red point denotes C_thresh_ for a single cell. Each gray point represents the maximum concentration (C_max_) observed over the full timelapse for cells that do not form condensates. Black lines in the violin plots show the medians of C_thresh_ for each protein sequence. The expected change in C_thresh_ relative to the corresponding wild type (WT) fragment, based on previous studies, is indicated with an arrow pointing up for increased C_thresh_ or down for decreased C_thresh_. Solid horizontal lines are shown for WT sequences. * denotes statistically significant difference compared to the corresponding wild type threshold intensity (two-sided t-test, p values adjusted for multiple comparisons by applying the Bonferroni correction). The dashed black line denotes 0.06 μM protein concentration, the lowest protein concentration that we could reliably distinguish from background. p values: hnRNPA1 add many Y = 2×10^−32^; hnRNPA1 add fewer Y = 2×10^−51^; hnRNPA1 add 1 Y = 0.19; DDX4 all R to A = 4×10^−124^; DDX4 all F to A = 3×10^−10^; DDX4 1 F to A = 0.43; TDP-43 hydrophobic & aromatic to S = 7×10^−87^; TDP-43 aromatic to S = 8×10^−58^; TDP-43 all hydrophobic & aromatic to S = 7×10^−83^; EWS all Q to R = 2×10^−86^; EWS 3 polar to R = 2×10^−119^; EWS 1 polar to R = 2×10^−16^; DDX3 all K to Q = 2×10^−13^; DDX3 3 K to Q = 0.26; DDX3 1 K to Q = 0.73; TIAR-2 all Y to G = 2×10^−41^; TIAR-2 all S to A = 1.0; TIAR-2 1 Y to G = 0.0002. hnRNPA1 WT fragment, n = 102 cells; hnRNPA1 add many Y, n = 116 cells; hnRNPA1 add fewer Y, n = 204 cells; hnRNPA1 add 1 Y, n = 100 cells; DDX4 WT fragment, n = 203 cells; DDX4 all R to A, n = 307 cells; DDX4 all F to A, n = 194 cells; DDX4 1 F to A, n = 149 cells; TDP-43 WT fragment, n = 234 cells; TDP-43 hydrophobic & aromatic to S, n = 181 cells; TDP-43 aromatic to S, n = 219 cells; TDP-43 all hydrophobic & aromatic to S, n = 306 cells; EWS WT fragment, n = 224 cells; EWS all Q to R, n = 146 cells; EWS 3 polar to R, n = 275 cells; EWS 1 polar to R, n = 82 cells; DDX3 WT fragment, n = 206 cells; DDX3 all K to Q, n = 134 cells; DDX3 3 K to Q, n = 186 cells; DDX3 1 K to Q, n = 140 cells; TIAR-2 WT fragment, n = 154 cells; TIAR-2 all Y to G, n = 297 cells; TIAR-2 all S to A, n = 88 cells; TIAR-2 1 Y to G, n = 258 cells.

Next, we performed live-cell time-lapse imaging with the small sequence library fused to the 24-mer FTH1 oligomerization domain. To map the concentration-dependence of condensate formation for each sequence in our library at fine resolution, we induced protein expression at time 0 and imaged from ~1 hour, every 35 minutes over 28 hours (48 timepoints) (Extended Data Fig. 1A). For each protein sequence, we defined a threshold concentration (C_thresh_) as the protein concentration at which condensates were first observed (Extended Data Fig. 1B). Over two independent biological replicates, we detected single barcodes for 28,119 of 50,428 tracked cells (68% of tracked cells with barcodes; 82% of them with single barcode), with high reproducibility of the fraction of cells with condensates, the fraction of GFP signal within condensates, and the threshold concentration (Extended Data Fig. 1C–D, Extended Data Table 1, Methods). We performed a corresponding ‘traditional’ arrayed imaging screen, in which each well contained cells expressing a single barcode, such that *in situ* SBS was not required, and confirmed close agreement with our CondenSeq results (Methods, Extended Data Fig. 1E–F).

Importantly, CondenSeq recapitulated previously reported sequence-dependent trends for protein sequences from hnRNPA1^15^, DDX4^14^, TDP-43^55^, EWS^56^, DDX3^57^, and TIAR-2^58^. We designed fragments that varied the known condensate-promoting sequence features and observed threshold concentration changes in the expected directions (Extended Data Fig. 1G; two-sided t-test, effects were all in the expected directions, but were not always statistically significant).

CondenSeq measurements capture the overall condensate formation propensities of protein sequences in a cellular context, which are the result of both the intrinsic biophysical properties of the test proteins and their heterogeneous interactions with cellular factors *in vivo* (Supplementary Figure 1A). Consistently, although CondenSeq measurements qualitatively recover sequence features that have previously been shown to drive condensate formation, we do not observe a simple quantitative relationship between C_thresh_ and *in vitro* saturation concentration (C_sat_) (Supplementary Figure 5A). Thus, CondenSeq measurements should not be interpreted as measurements of *in vitro* condensate formation propensity.

Time-lapse live-cell imaging is a powerful way to precisely map threshold concentrations, but is relatively resource intensive, making it challenging to substantially scale up the size of the sequence library. Thus, in subsequent larger experiments, we imaged live cells at a single timepoint and calculated the fraction of cells that contain condensates within a specified narrow concentration range (*f_condensates_*). *f_condensates_* values are correlated with threshold concentrations (Supplementary Figure 5B).

### A large CondenSeq screen of thousands of synthetic sequences

**Extended Data Fig. 2:**
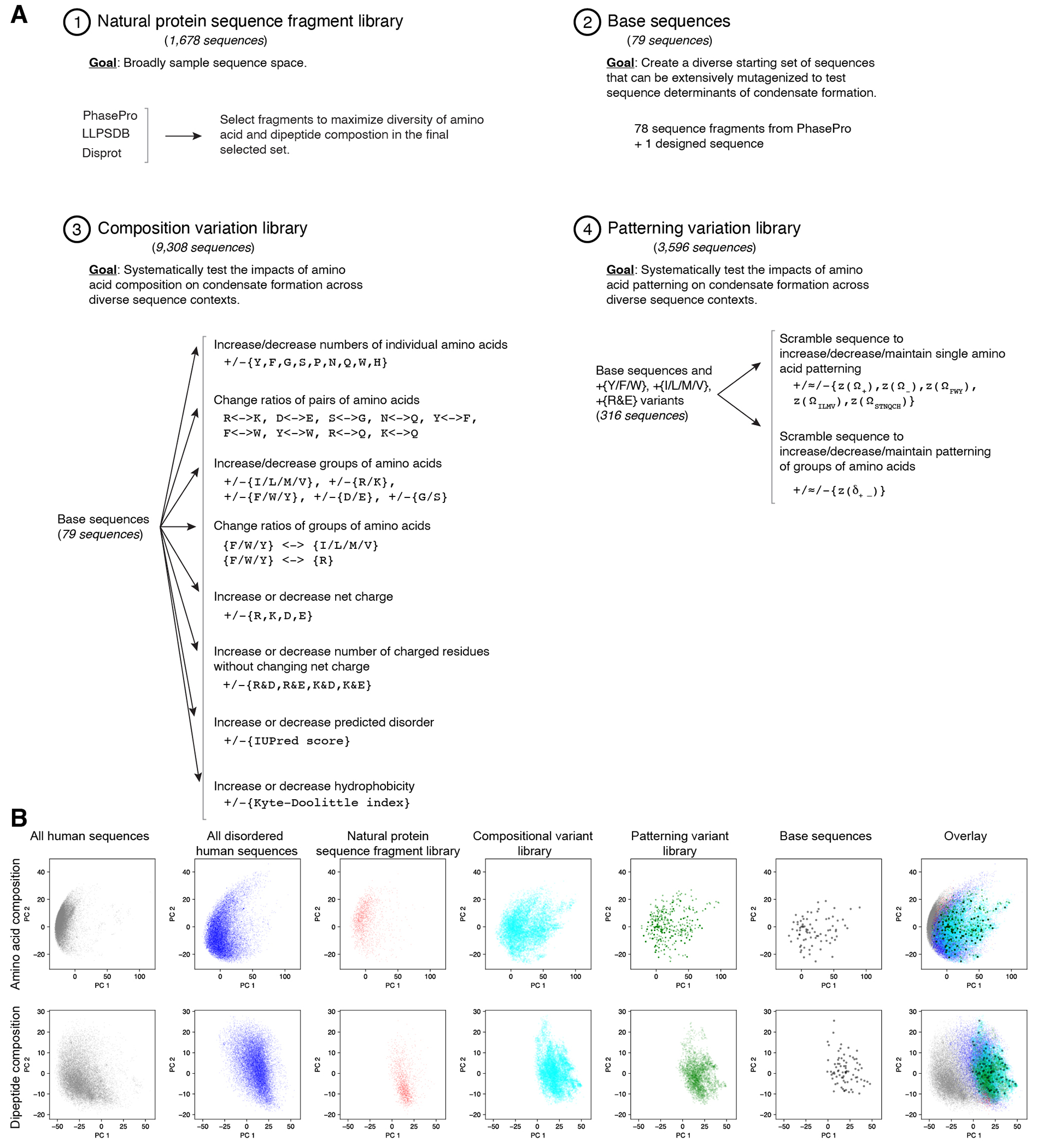
Details of protein sequence libraries. (**A**) Composition of the large protein sequence library. (**B**) Principal component analysis of the amino acid composition and dipeptide composition of all sequences in the large protein sequence, as well as all sequences in the human proteome, and all disordered regions in the human proteome.

**Fig. 2.**
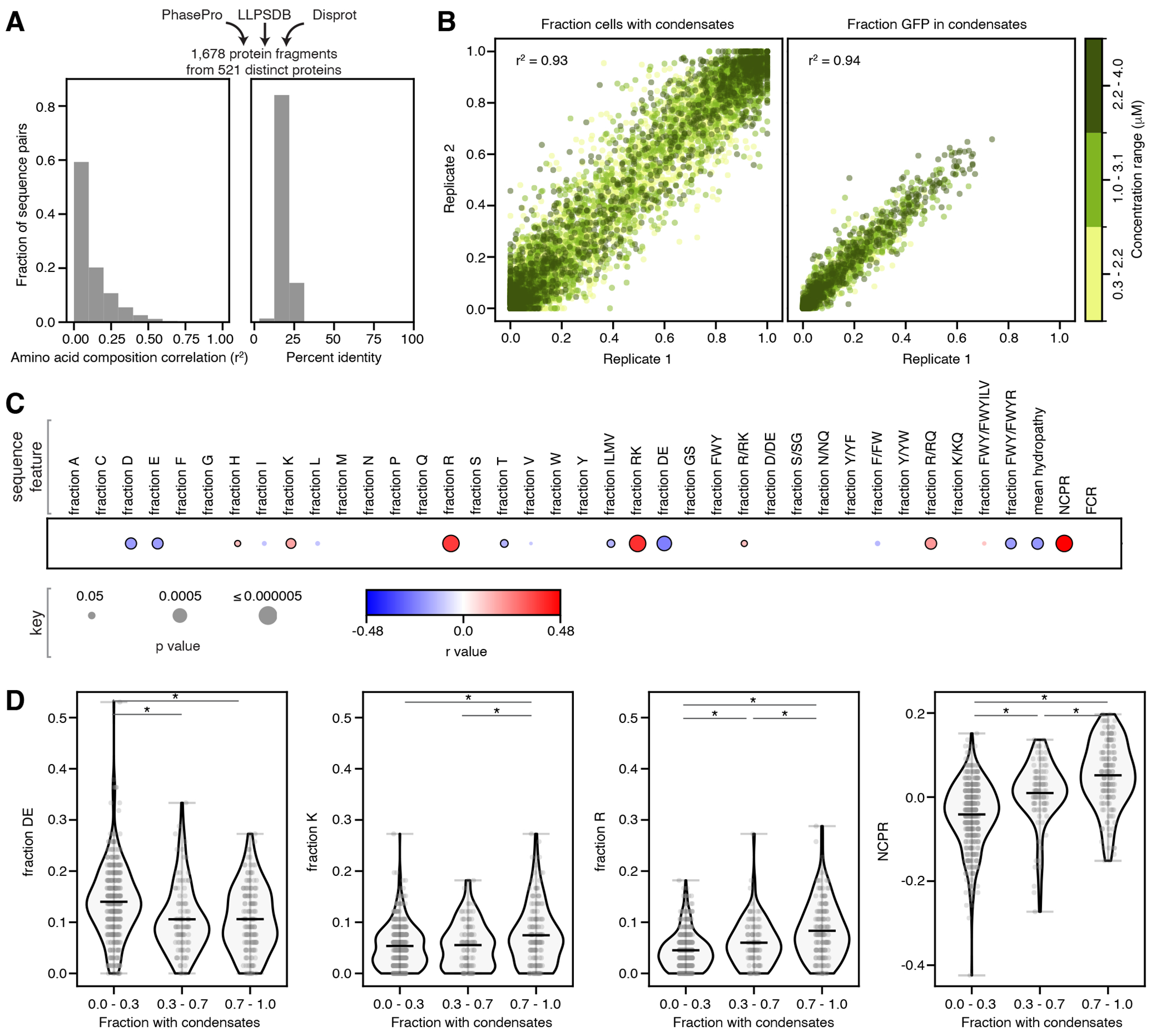
Characterization of a diverse library of protein sequences. (A) Summary of the natural protein sequence fragment set composition and sequence similarity. Left: Histogram of amino acid composition correlation (r^2^) for all pairs of sequences in the library (two sequences that contain the same amino acids, but in different orders would have correlation of 1). Right: Histogram of the percent sequence identity between all pairs of sequences in the library. (B) Experimental reproducibility for one GFP sub-library. Each point represents one protein sequence with expression in a defined concentration bin indicated by the color of the point. (C) Correlation between sequence features and *f*_*condensates*_ in the medium concentration bins (GFP fusions). Dots for sequence features with two-sided p values < 0.05 are outlined in black. p values are adjusted for multiple comparisons by applying the Bonferroni correction. (D) Protein sequence features versus the fraction of cells with condensates for GFP fusions. Each point represents a protein sequence. Black lines show the mean values. * denotes statistically significant difference (one-way ANOVA followed by Tukey post-hoc test, p values: fraction DE 0.0-0.3 vs 0.3-0.7 = 3×10^−5^; fraction DE 0.0-0.3 vs 0.7-1.0 = 6×10^−7^; fraction K 0.0-0.3 vs 0.7-1.0 = 9×10^−6^; fraction K 0.3-0.7 vs 0.7-1.0 = 0.005; fraction R 0.0-0.3 vs 0.3-0.7 = 0.006; fraction R 0.0-0.3 vs 0.7-1.0 = 1×10^−16^; fraction R 0.3-0.7 vs 0.7-1.0 = 8×10^−5^; NCPR 0.0-0.3 vs 0.3-0.7 = 2×10^−8^; NCPR 0.0-0.3 vs 0.7-1.0 = 1×10^−16^; NCPR 0.3-0.7 vs 0.7-1.0 = 8×10^−5^). Gray bars at the top and bottom of each violin plot show the minimum and maximum values. *f*_*condensates*_ 0-0.3: n=421 sequences; *f*_*condensates*_ 0.3-0.7: n=104 sequences; *f*_*condensates*_ 0.7-1.0: n=160 sequences.

We next sought to systematically test and discover the underlying sequence features that drive nuclear condensate formation, and to assess the consistency of their effects across diverse sequence contexts. To this end, we designed a library of 14,622 synthetic protein sequences, each 66 amino acids long, (“large sequence library”) to characterize with CondenSeq in HeLa cells (Supplementary Data 3). The library contained three subsets of sequences: (**1**) a “natural protein sequence fragment set” (Fig. 2A), (**2**) a “compositional variation sequence set”, and (**3**) a “patterning variation sequence set”, described in more detail below (Extended Data Fig. 2).

**Extended Data Fig. 3:**
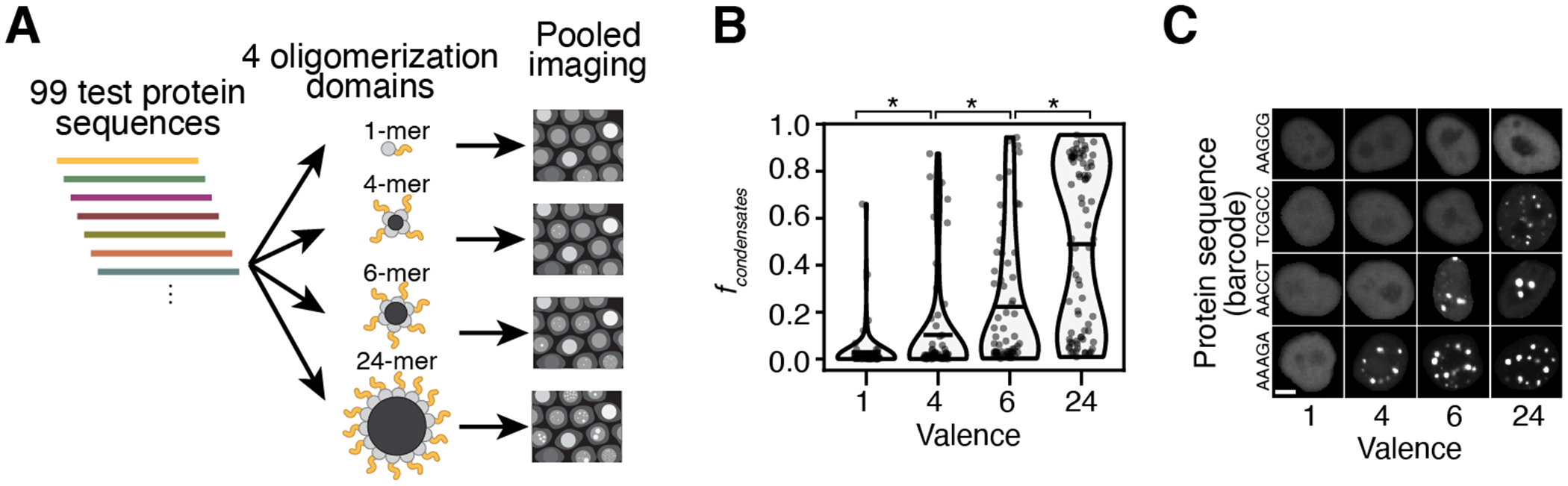
Assessing the impact of valence on condensate formation. (**A**) Schematic of the experiment to test the effect of protein valence on condensate formation. The small sequence library is fused to GFP and four different oligomerization domains resulting in valence 1, 4, 6, or 24, then cells are imaged and barcodes are read out. (**B**) Fraction of cells that contain condensates for the small sequence library fused to GFP and each of the four different oligomerization domains. Each point represents one protein sequence. Black lines show the means. The increases in *f*_*condensates*_ as valence is increased are all statistically significant (valence = 1 vs 4: p = 0.002; 4 vs 6: p = 2×10^−6^; 6 vs 24: p = 6×10^−10^, two-sided paired t-test, after Bonferroni correction, medium test protein concentration bin). (**C**) Example images of cells (masked nuclei) expressing protein sequences (rows) fused to GFP and each oligomerization domain (columns). These example images are representative of the following numbers of cells for which we collected data in our defined concentration bins: 1933 (AAGCG, valence=1), 1629 (AAGCG, valence=4), 1040 (AAGCG, valence=6), 760 (AAGCG, valence=24), 1360 (TCGCC, valence=1), 1730 (TCGCC, valence=4), 1492 (TCGCC, valence=6), 1348 (TCGCC, valence=24), 2775 (AACCT, valence=1), 3722 (AACCT, valence=4), 3445 (AACCT, valence=6), 2238 (AACCT, valence=24), 629 (AAAGA, valence=1), 761 (AAAGA, valence=4), 736 (AAAGA, valence=6), 487 (AAAGA, valence=24). Scale bars denote 5 μm.

We assayed the small sequence library fused to multiple different oligomerization domains and found that the valence = 24 oligomerization domain provided an optimal dynamic range for studying the sequence features that drive nuclear condensate formation (Supplementary Results, Extended Data Fig. 3, Supplementary Figure 5C, Supplementary Figure 6). Because our goal for the large sequence library was to test and discover the underlying sequence features driving nuclear condensate formation, we chose to characterize our entire large sequence library fused to this 24-mer oligomerization domain.

We split the large sequence library into three approximately equal sized sub-libraries for ease of handling, so that each CondenSeq sample assayed at most ~5,000 protein sequences. After imaging, *in situ* sequencing, and analysis, our final dataset included measurements for a median of 220 cells per protein sequence (Supplementary Figure 7A–C). Because condensate formation is concentration-dependent, we separately analyzed cells with test protein expression in narrow concentration bins (Methods; GFP: approximately 0.3-2.2μM (low), 1.0-3.1μM (medium), and 2.2-4.0μM (high)). Results were reproducible between replicates, between sub-libraries, for identical protein sequences with different barcodes, between different fluorescent protein fusions (GFP vs. SNAP-tag), in a second cell type (U2OS cells), and were consistent with results from the small sequence library (Supplementary Results, Fig. 2B, Supplementary Figure 7D–F, Extended Data Table 2, Extended Data Table 3, Supplementary Figure 8).

### Net charge per residue correlates with condensate formation

To systematically assess fundamental protein sequence features that drive condensate formation in the nucleus, we first analyzed results of our natural protein sequence fragment set, which contained 1,678 diverse protein sequence fragments, each 66 amino acids long, from naturally occurring proteins, mostly predicted to be disordered (Fig. 2A, Extended Data Fig. 2, Supplementary Figure 9A, Extended Data Table 4, Supplementary Note 1). We are not using these short protein sequence fragments to make conclusions about the behavior of the full-length proteins from which they are derived, but rather to identify general underlying sequence features that drive nuclear condensate formation. In the medium concentration bin, 38% of sequences formed condensates (*f_condensates_* ≥ 30%).

To assess which sequence features contributed to condensate formation, we calculated the Pearson correlation coefficients between *f_condensates_* for each test protein and each of a set of protein sequence features informed by previous studies^13–35^, including: the fraction of each amino acid out of the protein sequence (*e.g.*, fraction R), fractions of chemically similar groups of amino acids (*e.g.*, fraction RK), several ratios of these fractions (*e.g.*, fraction R/RK), and physicochemical properties (*e.g*., mean hydropathy and net charge per residue (NCPR)) (Methods).

Strikingly, charge-related parameters were the most strongly correlated with condensate formation. Protein sequences that formed condensates tended to have higher NCPR (Fig. 2C–D, Supplementary Figure 9B–D, Supplementary Table 2). NCPR was also higher on average for sequences that formed condensates *vs*. those that did not in our initial small libraries fused to other oligomerization domains (4-mer, 6-mer) (p < 0.008, two-sided t-test), suggesting that this effect is not specific to a particular oligomerization domain (Supplementary Figure 9E–F). Previous *in vitro* measurements have shown that NCPR far from 0 disrupts condensate formation^18, 62^. However, our measurements are performed in cells, where higher NCPR could drive condensate formation via interaction with other (negatively charged) cellular factors^20^. Our analysis of different classes of condensates, below, supports this idea and we investigate this possibility further in the Supplementary Results (Supplementary Figure 10). The importance of charge also raises the possibility that phosphorylation and other post-translational modifications might play important roles in regulating nuclear condensate formation (Supplementary Results, Supplementary Figure 11), consistent with previous observations^63, 64^.

### Mutagenesis shows importance of charge and aromatic residues

**Fig. 3.**
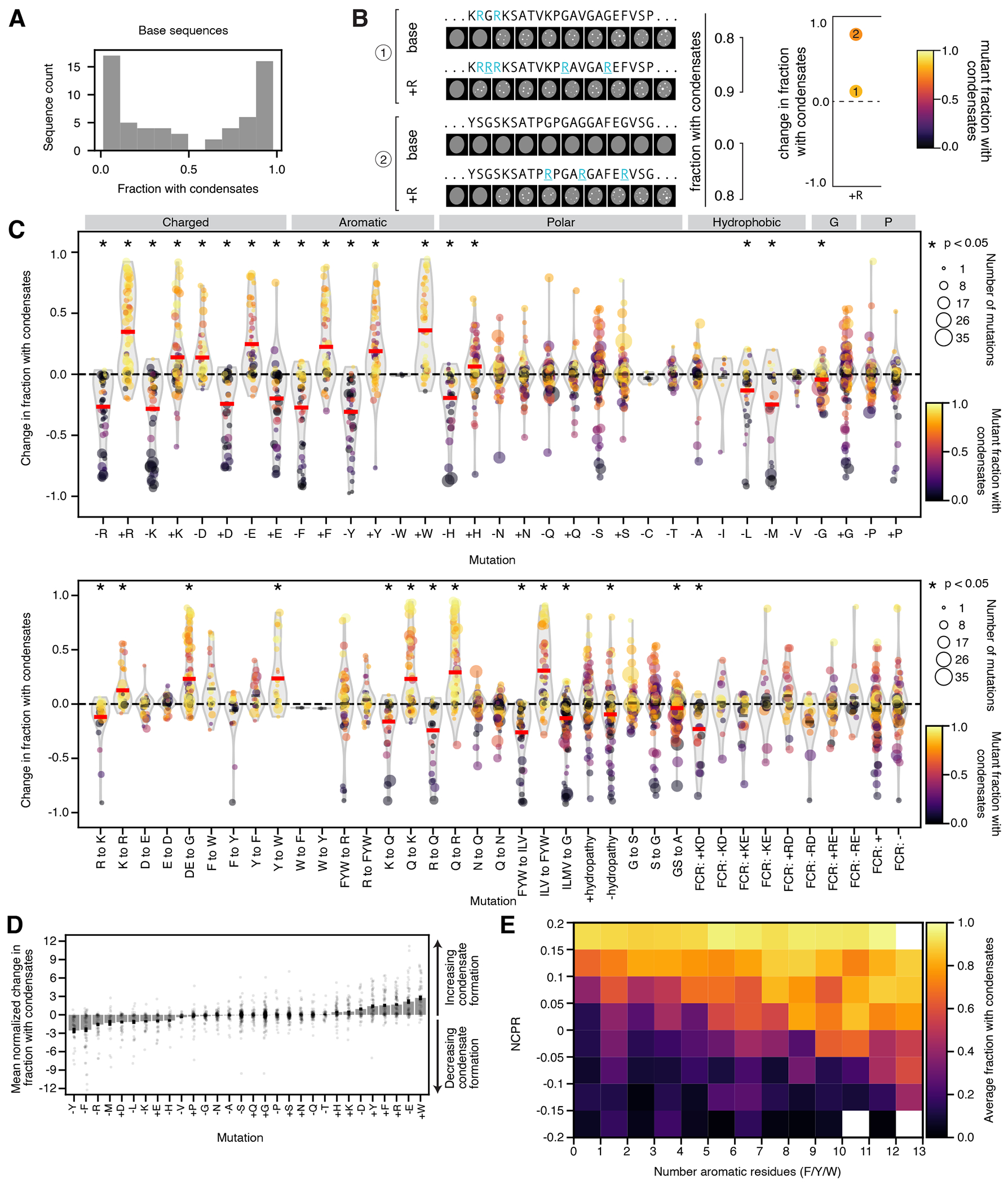
Large-scale mutagenesis to assess effects of amino acid composition on condensate formation. All data presented here is for GFP-fused sequences in the medium concentration bin. (A) *f*_*condensates*_ for base sequences. (B) Schematic showing how changes in *f*_*condensates*_ are plotted in (C) and (D). (C) Changes in *f*_*condensates*_ for mutant vs base sequences. Each dot represents one mutant sequence, its size indicates the number of mutations relative to the base sequence, and its color represents *f*_*condensates*_ for the mutant sequence. Each violin contains sequences designed to test the specified amino acids, with “–” denoting sequences that test removal of the amino acid (mutation to G or A) and “+” denoting sequences that test addition of the amino acid (mutation of G, S, or A to the specified amino acid). “R to K” denotes that arginine is mutated to lysine. “R to FWY” denotes that R is mutated to any of F, W, or Y. + and − hydropathy denote mutations that increase or decrease the average hydropathy index of the sequence (Kyte-Doolittle index). “FCR: +” and “FCR: -” include all mutant sequences that increase or decrease the fraction of charged residues, respectively, while keeping the net charge constant. “FCR: +KD” denotes equal numbers of K and D replacing G, S, or A residues. Asterisks denote groups with a significant change in the *f*_*condensates*_ and red lines show their mean values (p<0.05, two-sided Wilcoxon signed-rank test). p-values are adjusted for multiple comparisons by applying the Bonferroni correction. Black lines denote mean values for other groups. The dashed black line marks a change of 0, as a reference point. (D) Normalized effect on *f*_*condensates*_ per single amino acid mutation (Methods). Error bars represent the standard deviation of the mean. (C, D) Exact p-values and n values (number of sequences) provided in Supplementary Table 3. (E) All sequences from the natural protein sequence fragment set and the compositional variation sequence set binned by NCPR and number of aromatic residues; colors represent average values of *f*_*condensates*_ over all sequences in each bin. White boxes denote bins containing fewer than 10 sequences.

To determine the effects of amino acid composition more systematically, we turned to our compositional variation sequence set of 9,308 rationally designed variants of 79 “base sequences” (Extended Data Fig. 2, Extended Data Table 4). The base sequences span a range of amino acid compositions and propensities to form condensates (Supplementary Figure 12A), including a subset of 33 prion-like protein sequences (Methods), with mean *f_condensates_* per base sequence in the medium concentration bin of 0.48 (Fig. 3A, Supplementary Figure 12B), and mean predicted disordered fraction of 0.86 (Methods).

To test the importance of each amino acid, we substituted each instance of individual amino acids (mutating to G or A) or added instances (by mutating different numbers of G, A, or S residues to the amino acid), computed the change in *f_condensates_* relative to the base sequence (Fig. 3B), and then calculated the mean effect of the mutation type across all base sequences (Fig. 3C, red/black horizontal lines). Mutating charged and aromatic residues had the most consistent impacts on condensate formation, with positively charged (R or K) and aromatic (Y, F, or W) residues promoting condensate formation and negatively charged residues (D or E) reducing condensate formation (Fig. 3C, Supplementary Table 3, Supplementary Figure 12C). The importance of aromatic residues is supported by several previous studies^14, 15, 18, 19, 65^. To compare between different amino acids more directly, while controlling for the differing numbers of mutations per variant, we calculated a normalized effect per single amino acid substitution (Methods, Fig. 3D, Supplementary Figure 12D–F). Again, we found that aromatic and charged residues had the biggest effects on condensate formation. Strikingly, adding tryptophan more strongly promoted condensate formation than any other mutation type. Mutating one amino acid type to another further revealed differences between chemically similar amino acids, consistent with previous experimental and computational results^66, 67^ (Supplementary Results, Fig. 3C, bottom, Supplementary Figure 12C, bottom).

The importance of NCPR and aromatic residues is especially clear when binning all sequences by NCPR and the number of aromatic residues, and computing the average *f_condensates_* per bin (Fig. 3E, Supplementary Figure 12G): within the range of values tested here, higher NCPR and more aromatic residues promoted condensate formation, but lower NCPR could be largely compensated by increased numbers of aromatic residues, and similarly, low numbers of aromatic residues could be compensated by higher NCPR.

### Amino acid patterning contributes to condensate formation

**Extended Data Fig. 4:**
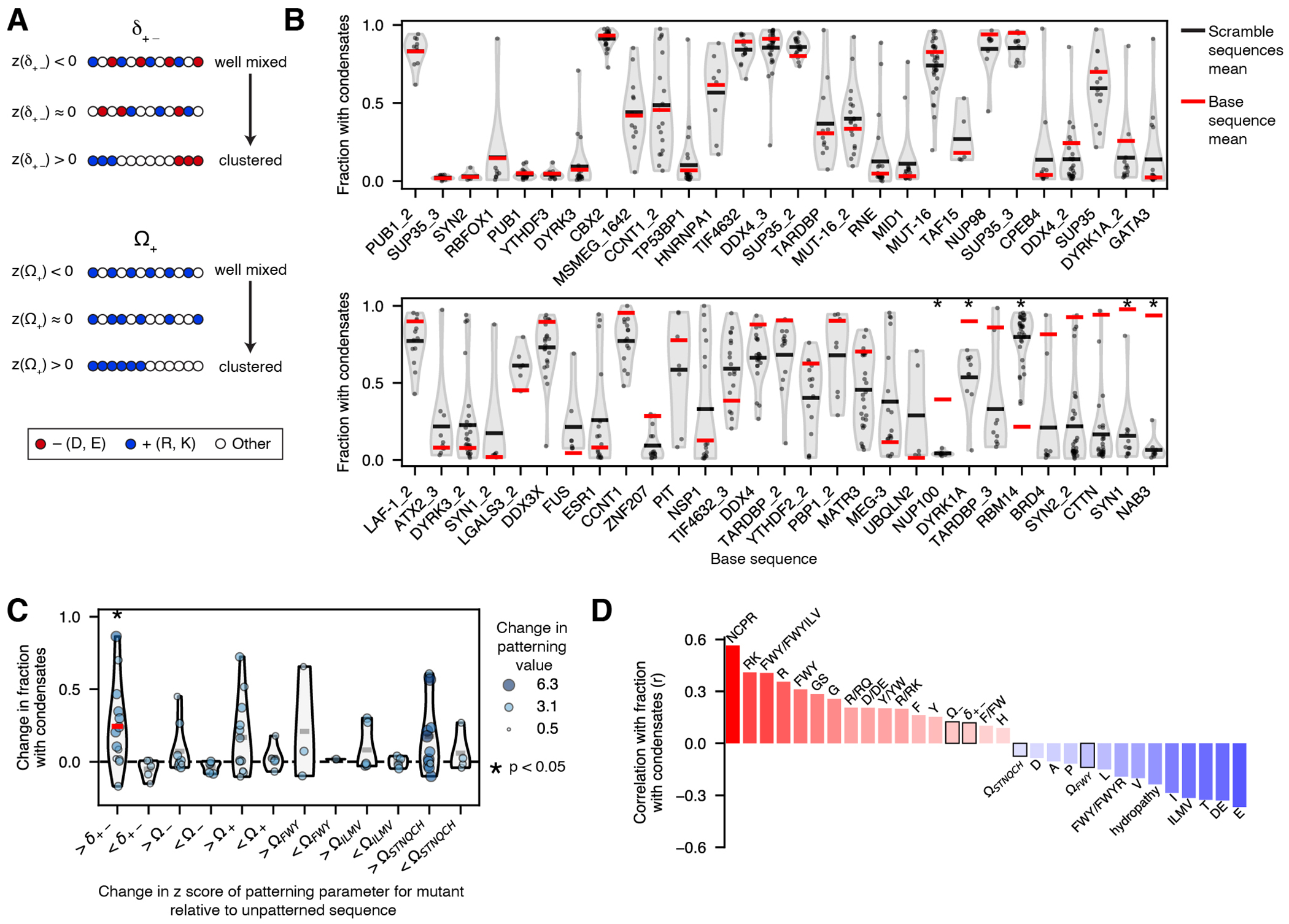
Systematic assessment of the effects of amino acid patterning on condensate formation. All data presented in this figure is for sequences fused to GFP in the medium concentration bin. (**A**) Schematic of patterning parameters. Each string of circles represents a protein sequence, with each circle representing a single amino acid. Negative z-scores for patterning parameters indicate well-mixed amino acids of the specified type, while more positive z-scores indicate higher segregation of the specified amino acids. z-scores are computed with NARDINI^1^. (**B**) Each violin shows *f*_*condensates*_ for all scrambled versions of the specified base sequence. Each black dot represents a single scrambled sequence. Red bars denote the values for the base sequences. Black bars denote the means of the scrambled sequences. Violins are ordered by the difference between the means of the base and scrambled sequences (low to high). * denotes base sequence values (red bars) that are statistically unlikely, given the given the distribution of *f*_*condensates*_ values for all of the scrambled variants of that base sequence (black dots) (smoothed empirical CDF test, see Supplementary Note 1 for detailed description of this test; p values: NUP100 = 0.0003, DYRK1A = 0.006, RBM14 = 0.003, SYN1 = 0.004, NAB3 = 5×10^−13^). (**C**) The change in *f*_*condensates*_ for patterning mutants versus unpatterned sequences that do not form condensates (patterning score near 0, Supplementary Note 1). Each violin contains sequences that test the effect of a different patterning parameter. “>” and “<” indicate mutants that increase or decrease the designated patterning parameter. The mutants shown here have a substantial change only in the designated patterning parameter; for example for δ_+−_ mutants, there is little change in other patterning parameters. The colors and sizes of the dots indicate the change in the patterning value of the mutant sequence relative to the unpatterned sequence. Asterisks denote groups with a significant change in the fraction of cells with condensates and red lines show their mean values (two-sided Wilcoxon signed-rank test; >δ_+−_ p value = 0.04). Gray lines denote the mean values for other groups. p values are adjusted for multiple comparisons by applying the Bonferroni correction. The dashed black line is shown as a reference point marking a change of 0, that is no difference between mutant and base sequences. (**D**) Correlation between sequence features and *f*_*condensates*_ for all large library sequences that contain at least 5% positively charged, negatively charged, aromatic, hydrophobic, and polar amino acids (so that all patterning parameters can be computed for all sequences). The colors of the bars represent the Pearson correlation (r value); bars are only shown if two-sided p values are less than 0.05. p values are adjusted for multiple comparisons by applying the Bonferroni correction. Black outlines denote bars for patterning parameters.

Previous work has suggested that the spacing of particular types of amino acids, especially charged and aromatic residues, within a sequence can impact condensate formation^14, 17, 19, 20, 31, 68^. Indeed, results from our patterning variation sequence set suggested that amino acid patterning, particularly of charged residues, can affect condensate formation (Extended Data Fig. 4, Supplementary Figure 13). We describe these results in detail in the Supplementary Results.

### Consistency of effects across sequence contexts

**Extended Data Fig. 5:**
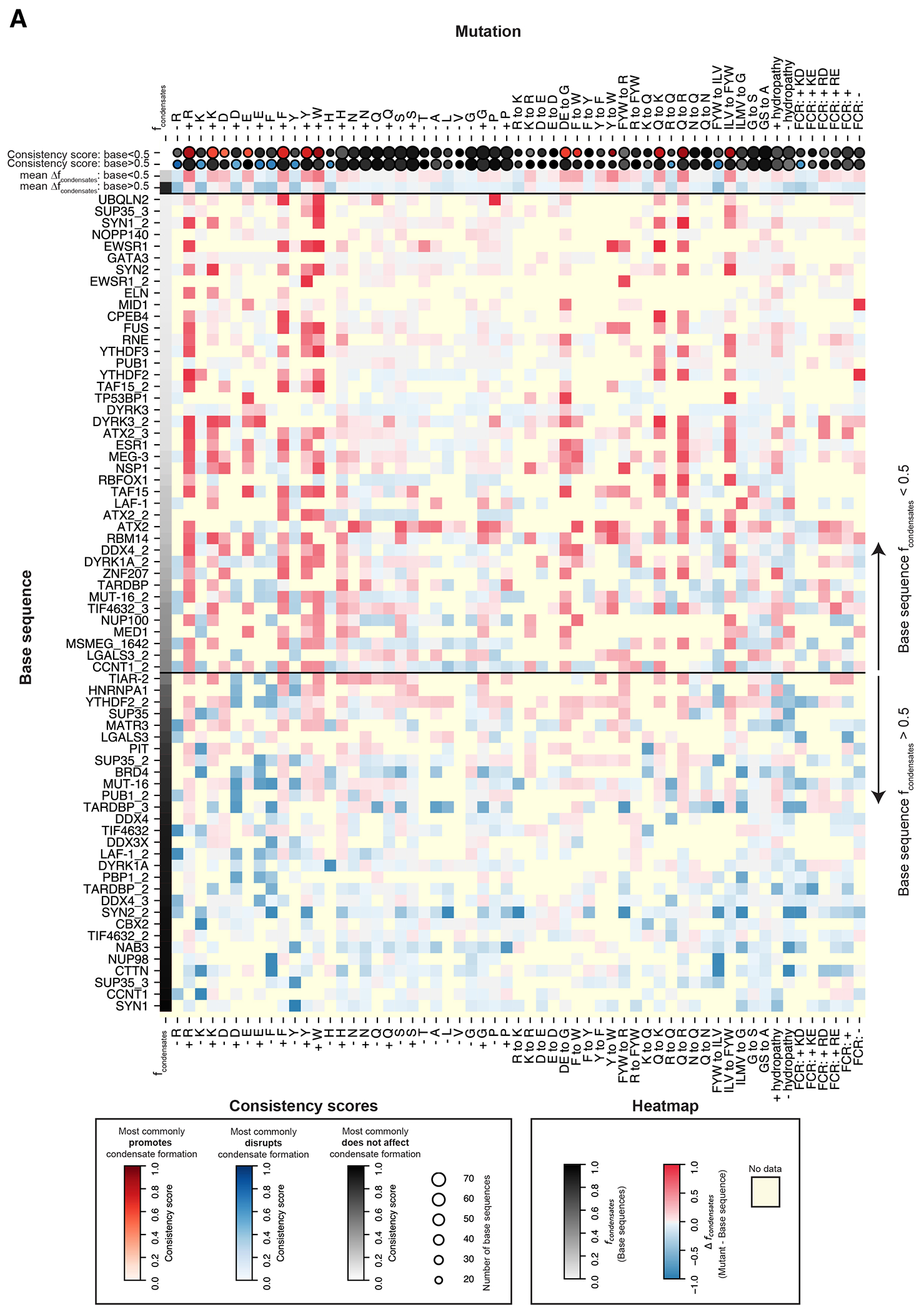
Assessing the impacts of different types of mutations across many sequence contexts. (**A**) The change in the propensities for mutant sequences versus base sequences to form condensates. The values in the heatmap are the mean values of *f*_*condensates*_ for all mutations of the specified type minus *f*_*condensates*_ for the base sequence. All values plotted are for the GFP fusions in the medium concentration bin. There may not be data for a given mutation type (box colored yellow) for one of two reasons: (1) it was not possible to make the mutation type for that sequence (for example, it is not possible to make a–R mutant if the base sequence does not contain any R residues); or (2) the sequence was not expressed within the GFP fusion medium concentration bin. The top two rows show consistency scores over the base sequences for which *f*_*condensates*_ is less than 0.5 or greater than 0.5, respectively. The consistency score indicates the fraction of base sequences over which the sequence feature has the most common effect (1.0 indicates that the sequence feature has the given effect across 100% of the base sequences) (Supplementary Note 1). The dot size indicates the number of base sequences for which there is data for the given sequence feature. The two rows below the consistency scores show the mean Δ *f*_*condensates*_ values for the base sequences for which *f*_*condensates*_ is less than 0.5 or greater than 0.5, respectively.

The unique scale of our CondenSeq experiments enabled us to explore the consistency of the effects of sequences features across different base sequence contexts. We computed a “consistency score” for each sequence feature that we tested (Supplementary Note 1, Supplementary Table 4, Extended Data Fig. 5A), which reflects how often, across different sequence contexts, the sequence feature had the most common effect (promotes, disrupts, or causes no change to condensate formation). For example, removing arginine residues most commonly disrupted condensate formation, with a consistency score of 0.92, meaning that it lowered *f_condensates_* (or caused no change in *f_condensates_* for base sequences with low *f_condensates_* values) for 92% of the tested base sequences.

Mutations adding or removing aromatic and charged (especially arginine) residues had the highest consistency scores, suggesting their impacts are largely independent of base sequence context. Several other mutation types had lower consistency scores including those that alter the fraction of charged residues (without changing overall net charge), sequence hydropathy, or patterning of different groups of amino acids. Only two sequence features had no effect on condensate formation with consistency scores of 1.0, showing that nearly all sequence features affected condensate formation in some base sequence context.

### Features of proteins colocalizing with nucleoli or chromatin

Although our analysis thus far considered all condensate-forming sequences together, condensates exhibit morphological, biophysical, and compositional diversity^9^. We next tested if different sequence features were associated with distinct classes of condensates.

We first tested whether a small subset of sequences (59) colocalized with endogenous nuclear condensates (nuclear speckles, nucleoli, and Cajal bodies) via antibody staining and arrayed imaging (to avoid crosstalk between antibody and SBS channels). Most did not, and for the sequences that did colocalize, the small size of this library precluded identification of features that drove colocalization (Supplementary Results, Supplementary Figure 14, Supplementary Table 5).

**Fig. 4.**
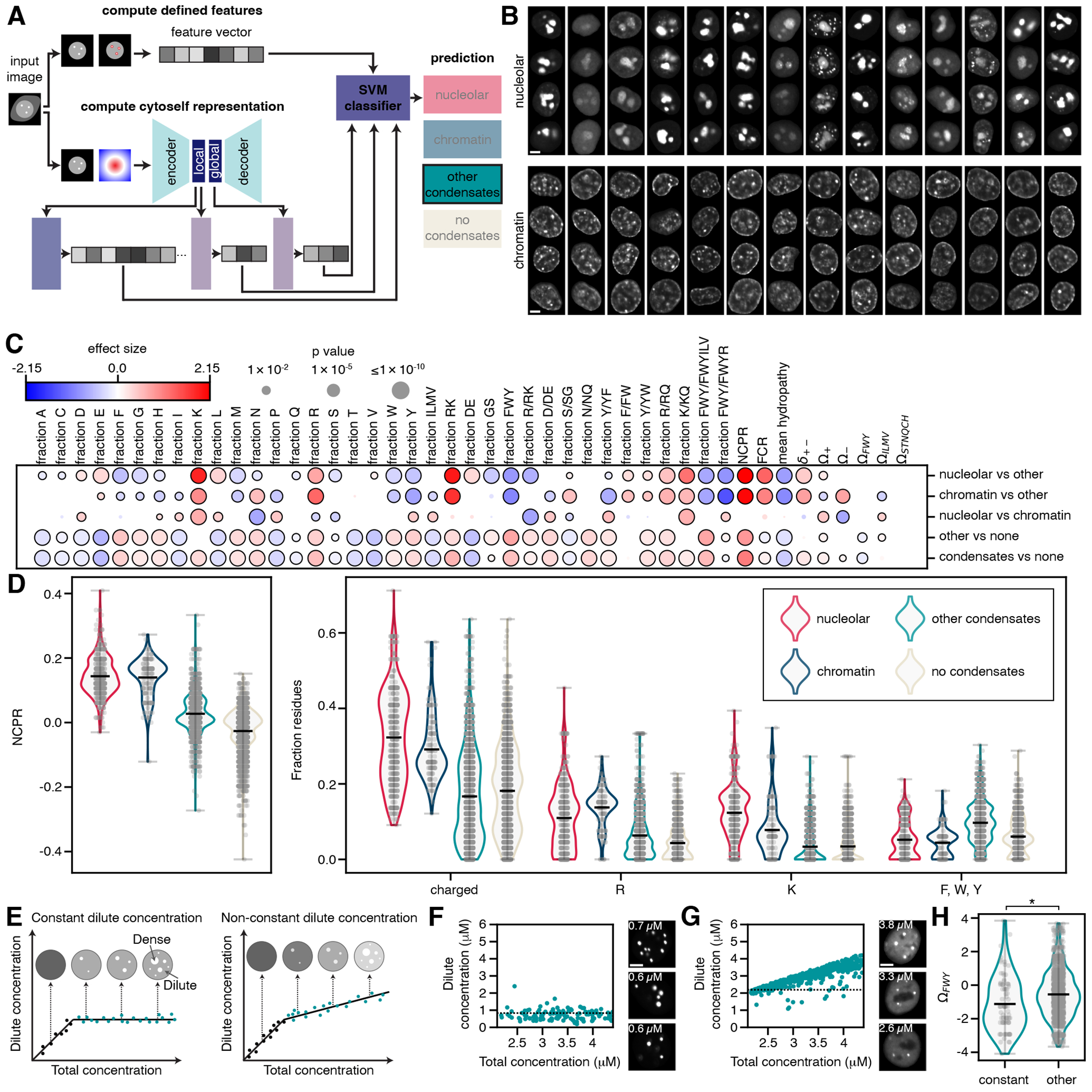
Image-based classification of different types of condensates. All data shown is for GFP fusions. (A) Schematic of the nucleolar and chromatin localization classifier. (B) Example images (masked nuclei) classified as nucleolar- (top) or chromatin-localizing (bottom). Each column contains four images of different cells that express the same protein sequence. Within our defined concentration bins, a total of 74,167 and 29,040 cells were classified as nucleolar- or chromatin-localizing, respectively. (C, D) Data in the medium concentration bin. (C) Comparisons of sequence features across protein classes. Sequences from the large sequence library are classified as nucleolar-localizing (“nucleolar”), chromatin-localizing (“chromatin”), forming other condensates (“other”), or forming none (“none”). Positive effect sizes (Cohen’s d, red) indicate higher values in the first group vs. the second. Black outlines denote Bonferroni-corrected p < 0.05 (two-sided t-test). (D) NCPR, fraction charged, arginine, lysine, and aromatic residues for each group of protein sequences. Each dot represents one protein sequence. Black lines show mean values. n=608 nucleolar, n=162 chromatin, n=3053 other condensates, n=3157 no condensates sequences. (E) Schematic of constant vs non-constant dilute concentration. Each dot represents a cell with condensates (blue) or without (black). Total concentration refers to the protein’s overall nuclear concentration, while dilute concentration is its concentration outside condensates. (F and G) Left: Dilute vs total concentration for example sequences classified as “constant” (F; GFP sub-library 2, barcode TATATCCG) or “other” (G; GFP sub-library 2, barcode CGGGTAAT). Cells without condensates are not shown. The dashed black line marks the average dilute concentration for cells in the lowest 10th percentile of total concentration. Right: Three example images (masked nuclei) for the same sequence shown on the left with total concentration from 3-4 μM (F) or 2.8-4.4 μM (G). Dilute concentration is indicated on images. Out of 2,150 condensate-forming sequences, 105 were classified as “constant”. (H) Fraction aromatic residues and Ω_FWY_ z-scores for “constant” vs. other condensate-forming sequences. Black lines indicate means. p = 0.02 (two-sided t-test, Bonferroni corrected). Scale bars: 5 μm. Gray bars at top and bottom of violin plots (D, H) mark min/max values. n=105 constant sequences, n=2045 other sequences.

We hypothesized that we could increase our power by identifying colocalization with endogenous condensates with highly distinctive morphologies from our GFP-fusion images alone, without antibody staining. To this end, we used cytoself, a recently-developed self-supervised deep learning model^69^, to extract latent representations from each image, and combined this information with defined condensate features calculated directly from the images, to create a “feature vector” for each image (Supplementary Note 1). Using a set of manually labeled images, we trained an SVM classifier to use these features to predict whether a sequence localized to the nucleolus or chromatin (Fig. 4A–B). The classifier achieved 95-96% accuracy on a set of held-out images (Supplementary Figure 15A–B), and accurately identified all sequences from the antibody staining experiment that colocalize with the nucleolus. Additionally, the sequences identified as chromatin-localizing by the classifier had significantly higher correlation with the Hoechst signal than all other sequences (two-sided t-test, p < 0.00001; mean Pearson’s *r* = 0.60 for chromatin-localizing sequences and 0.22 for other sequences; Supplementary Figure 15C). Based on the classifier, up to 12.1% and 3.7% of the large library sequences colocalized with the nucleolus and chromatin, respectively (Supplementary Table 6).

Several sequence features were significantly enriched in nucleolus- or chromatin-localizing sequences compared to other condensate-forming ones (Fig. 4C). Nucleolus- and chromatin-localizing proteins had, on average, higher NCPR than proteins that form other condensates or did not form condensates, in agreement with previous observations that high positive charge drives nucleolar localization^70^, as well as higher fractions of charged residues, arginine, and lysine, and lower fractions of aromatic residues (Fig. 4D, Supplementary Figure 15D). We confirmed that in many cases mutating positively charged residues was sufficient to alter a sequence’s localization pattern (Supplementary Table 7).

### Features associated with concentration-buffering capacity

One possible function of condensates is to buffer intracellular concentration^71^. Particularly, as the total concentration of a protein increases, condensates that form through single component phase separation, driven by self-association, maintain constant dilute phase concentration (concentration outside of the condensates)^38, 72^ (Fig. 4E). Because we collected data over a range of concentrations, we could identify protein sequences for which the dilute phase concentration remained approximately constant within the measured range (Fig. 4F–G, 105 “constant sequences” out of 2,150 total condensate-forming sequences, Methods). Compared to other condensate-forming sequences (“other sequences”), “constant sequences” were significantly enriched in aromatic residues, and had lower Ω_FWY_ values on average, indicating that the aromatic residues tended to be more evenly spaced out (p < 0.05, two-sided t-test, after Bonferroni correction, Fig. 4H, Supplementary Figure 15E). These results are consistent with a model in which aromatic residues can drive self-association, in agreement with several previous studies^14, 15, 18, 19, 65^, and suggest that the spacing of these residues impacts concentration-buffering capacity. Previous work suggests that evenly spaced aromatic residues can also prevent aggregation^15^, a possibility that is not mutually exclusive with our findings. The sequence features enriched in the concentration-buffering sequences were distinct from those driving nucleolus- and chromatin-localization, suggesting that different physicochemical features drive the formation of different types of condensates.

### Assessing the impacts of intermolecular chemical specificity

**Extended Data Fig. 6:**
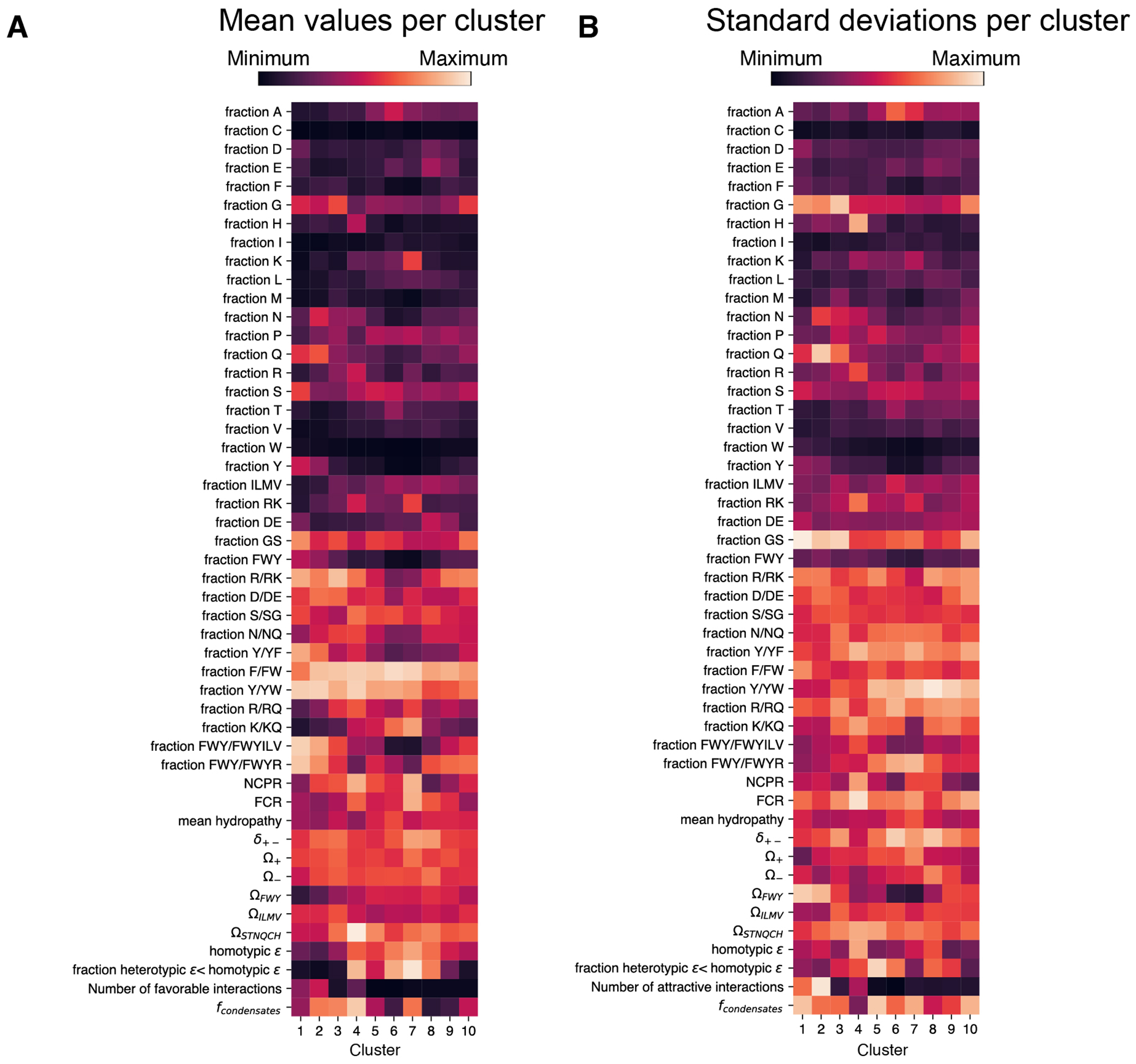
Features of sequences in clusters with distinct intermolecular chemical specificities. (**A**) Mean features of the sequences in each cluster (Fig. 5b) with expression in the medium concentration bin (GFP fusions). Homotypic ε is the FINCHES interaction parameter for the interaction of the test protein sequence with itself. The fraction heterotypic ε < homotypic ε is the fraction of the FINCHES interaction parameters for a test protein sequence with all human IDRs that are less than the homotypic ε value for that test protein sequence. The number of favorable interactions means the number of human IDRs with which a test protein has an attractive FINCHES interaction parameter (less than −3). The minimum and maximum values for the color scale are as follows: 0 to 0.27 for the fraction of each individual amino acid (for example, fraction A); 0 to 0.4 for the fraction of each group of amino acids (for example, fraction ILMV); 0 to 1.0 for the relative fractions of amino acids or amino acid groups (for example, fraction R/RK or fraction FWY//FWYILV); −1.31 to 1.31 for the patterning features (for example, δ_+−_); −0.21 to 0.21 for NCPR; 1 to 6 for mean hydropathy; −12 to 12 for homotypic ε; 0 to 1 for the fraction heterotypic ε < homotypic ε; 0 to 3000 for the number of favorable interactions; 0 to 1 for f_condensates_ (medium concentration bin, GFP fusion). (**B**) Standard deviations of the sequence features for each cluster. Clusters are the same as those shown in (**A**). The minimum and maximum values for the color scale are as follows: 0 to 0.15 for the fraction of each individual amino acid (for example, fraction A); 0 to 0.15 for the fraction of each group of amino acids (for example, fraction ILMV); 0 to 0.44 for the relative fractions of amino acids or amino acid groups (for example, fraction R/RK or fraction FWY//FWYILV); 0 to 2 for the patterning features (for example, δ_+−_); 0 to 0.1 for NCPR; 0 to 1 for mean hydropathy; 0 to 5 for homotypic ε; 0 to 0.3 for the fraction heterotypic ε < homotypic ε; 0 to 900 for the number of favorable interactions; 0 to 0.4 for f_condensates_ (medium concentration bin, GFP fusion).

**Fig. 5.**
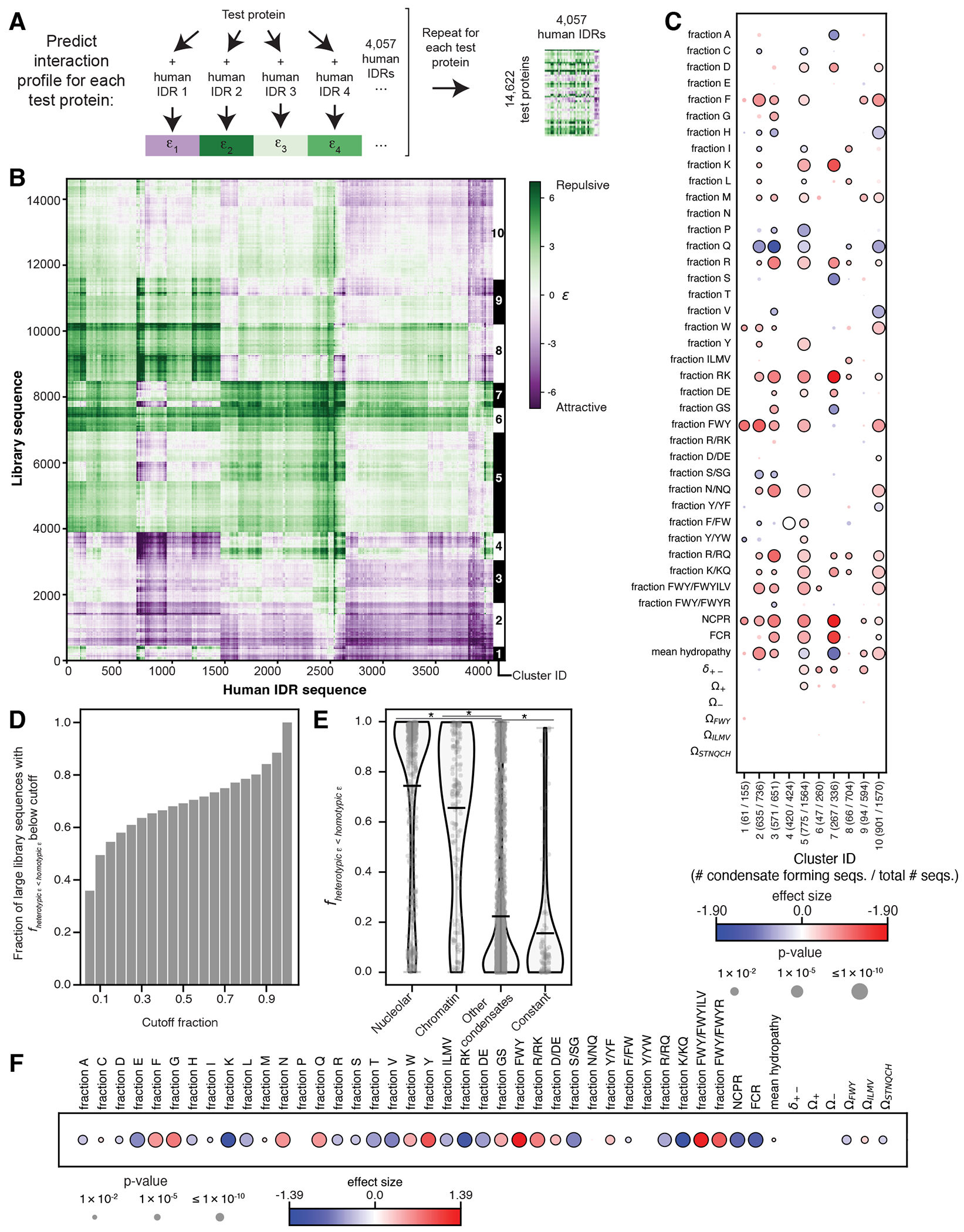
Classifying sequences based on predicted intermolecular chemical specificity. (A) Prediction of intermolecular chemical specificity profiles with FINCHES^45^. (B) Grouping test proteins by their predicted intermolecular chemical specificities. Predicted interaction values (ε, color bar) between human IDRs longer than 100 amino acids (columns) and test proteins from the large sequence library (rows), clustered via hierarchical clustering into 10 clusters. Cluster IDs/labels (1-10) are shown on the right with alternating black and white rectangles. (C) Comparisons of sequence features for test proteins that form condensates versus those that do not in each cluster (GFP fusions, medium concentration bin). Positive (red) / negative (blue) effect sizes (Cohen’s d) indicate higher/lower values for condensate-forming sequences. Black outline: p values < 0.05, Bonferroni-corrected (two-sided t-test). (D) The fraction of the large library sequences (y-axis) that form condensates (GFP fusions, medium concentration bin) with *f*_*heterotypic ε < homotypic ε*_ below different cutoffs (x-axis). (E) The distribution of *f*_*heterotypic ε < homotypic ε*_ (lower/higher fractions: homotypic/heterotypic interactions favored) (y-axis) for different classes of sequences (x-axis). “Nucleolar”: nucleolar-localizing sequences. “Chromatin”: chromatin-localizing sequences. “Other condensates”: sequences that form other condensates (not nucleolar or chromatin localizing). “Constant”: sequences that form condensates with concentration buffering capacity. * denotes statistically significant difference (two-sided t-test; p-values: nucleolar vs other = 9×10^−260^, chromatin vs other = 7×10^−65^, constant vs other = 0.03). Gray bars at the top and bottom of each violin plot show the minimum and maximum values. n=608 nucleolar, n=162 chromatin, n=3053 other condensates, n=105 constant sequences. (F) Differences in sequence features for test proteins that form condensates and have *f*_*heterotypic ε < homotypic ε*_ ≤ 0.1 vs. *f*_*heterotypic ε < homotypic ε*_ > 0.1 (homotypic vs. heterotypic interactions predicted to be favored). Effect sizes (Cohen’s d) and significance (p-value, two-sided t-test, circle size) for each sequence feature (columns). Red/blue: values of the sequence feature are higher for sequences with *f*_*heterotypic ε < homotypic ε*_ ≤ 0.1 (homotypic favored) or for sequences with *f*_*heterotypic ε < homotypic ε*_ > 0.1 (heterotypic favored), respectively. Black outline: p values < 0.05, Bonferroni-corrected.

While CondenSeq measures condensate formation in the biologically relevant nuclear environment, it does not reveal the specific endogenous factors that each test protein interacts with. These interactions are likely heterogeneous — different test proteins may interact with different sets of endogenous factors. To gain more insight into the impacts that these heterogeneous interactions may have on the sequence features that promote nuclear condensate formation, inspired by recent work^45^, we used FINCHES^45^ to predict interactions between our test proteins and all endogenous human IDRs longer than 100 amino acids (4,057 sequences) (Fig. 5A). This resulted in a predicted “interaction profile” for each test protein — a vector of predicted interaction parameters (ɛ values; negative values are attractive, positive values are repulsive) between the test protein and each human IDR (vector length = 4,057). A predicted interaction profile represents the intermolecular chemical specificity of a test protein. Importantly, individual interaction parameters quantify the favorability of particular interactions in isolation, but do not necessarily imply whether the interactions actually occur in cells. We thus do not use these profiles to suggest particular sets of interactions that test proteins are actually making, but rather use them only to identify groups of test proteins with similar intermolecular chemical specificities. To this end, we clustered the interaction profiles for all test proteins into 10 clusters, where each cluster represents a set of test proteins with similar predicted intermolecular chemical specificity (Fig. 5B, Extended Data Fig. 6).

By comparing test proteins that form condensates versus those that do not within each cluster, we identified the sequence features that promote condensate formation for each cluster (Fig. 5C). Very few sequence features displayed opposing trends between different clusters. Mean hydropathy was one such example. This is consistent with the results from our compositional variation sequence set, which also suggested that hydropathy can have varied impacts on condensate formation, depending on the sequence context. The opposing trends for different clusters suggest that differing intermolecular chemical specificity may be responsible for this variability. The vast majority of sequence features do not display opposing trends, but do vary in significance between different clusters. For example, a higher fraction of aromatic residues is associated with condensate formation only for 5 of the 10 clusters, but none show the opposite association. Similarly, higher NCPR is associated with condensate formation only for 7 of the 10 clusters. Although this analysis has limitations — these are *predicted* interaction profiles and they do not include interactions with folded protein domains or with nucleic acids, as tools for such analysis have yet to be developed — it suggests that intermolecular chemical specificity and interactions with endogenous factors can tune the relative impacts of sequence features on nuclear condensate formation.

Finally, we extended this analysis to gain insight into the relative contributions of homotypic versus heterotypic interactions to nuclear condensate formation. We used FINCHES^45^ to predict homotypic interaction (self-interaction) strength (homotypic ε) for each test protein in the large sequence library. We then calculated the fraction of the predicted interaction strengths between that test protein and all human IDRs (“heterotypic ε values”) that are less than the homotypic ε value for that test protein (*f_heterotypic ε<homotypic ε_*). Lower *f_heterotypic ε<homotypic ε_* suggests that homotypic interactions are preferred, while higher *f_heterotypic ε<homotypic ε_* suggests that heterotypic interactions are preferred. 49% of the test proteins that form nuclear condensates have fractions less than 0.1 (Fig. 5D). Nucleolus- and chromatin-localizing test proteins have, on average, significantly higher *f_heterotypic ε<homotypic ε_* than other sequences that form condensates (mean values: nucleolus = 0.74, chromatin = 0.66, other condensates = 0.22; p-values < 0.005; two-sided t-test; Fig. 5E), suggesting that heterotypic interactions drive this co-localization. Test proteins that exhibited concentration-buffering capacity have, on average, significantly lower *f_heterotypic ε<homotypic ε_* compared to other sequences that form condensates, suggesting that homotypic interactions dominate (mean value for concentration-buffering sequences = 0.16; p < 0.05; two-sided t-test) (Fig. 5E). Finally, for condensate-forming test proteins, we asked whether there are significant differences in sequence features between those for which homotypic vs. heterotypic interactions are predicted to be favored. We found many differences (Fig. 5F), including significantly lower NCPR and significantly higher fraction of aromatic residues for sequences in which homotypic interactions are favored. Together with our analysis of nucleolar- and chromatin- localizing sequences, sequences with concentration-buffering capacity, and sequences with distinct intermolecular chemical specificities, these results suggest that different sequence features drive the formation of different types of nuclear condensates.

## DISCUSSION

CondenSeq enables efficient characterization of the propensities of protein sequences to form nuclear condensates. Leveraging this approach to characterize a large library of synthetic sequences, we found that aromatic residues and higher NCPR consistently promote condensate formation across diverse sequence contexts, though likely through different mechanisms. Our analyses of nucleolar- and chromatin-localizing sequences, concentration-buffering sequences, together with our FINCHES-based analysis suggest that higher NCPR may promote condensate formation through heterotypic interactions, while aromatic residues may be more important for homotypic interactions.

CondenSeq is powerful, but has several key limitations. CondenSeq is currently limited to characterizing sequences within the nucleus, does not reveal interactions that a test protein may make with endogenous factors, nor the material properties or dynamics of condensates. Furthermore, cell type and choice of fluorescent protein fusion may affect the sequence determinants of nuclear condensate formation^73^. Our control experiments in HeLa vs. U2OS cells, and with GFP vs. SNAP-tag fusions, did not reveal substantial differences, but it will be interesting to investigate further in future work. We elaborate on the limitations of CondenSeq and approaches that may be required to overcome them in the Supplementary Discussion.

Potential future applications of CondenSeq include characterizing short linear motifs, amino acid extensions, and disease-associated proteins. It could be adapted to study RNA sequences or to learn the rules of condensate miscibility, and could be combined with functional assays and molecular profiling to disentangle the relationships between protein sequences, condensates, and molecular and cellular function. Finally, we envision that the data presented here and our high-throughput approach could provide the foundation for developing and improving models that predict nuclear condensate formation directly from protein sequence.

## Methods

### CondenSeq overview and timing

CondenSeq starts from a library of protein sequences and yields the propensity for each sequence to form nuclear condensates. The entire CondenSeq pipeline, from cloning to final analyzed data, can be completed over a period of approximately 4-5 weeks: 1 week to clone the library, perform quality control steps, and make lentivirus; 1 week to titer the lentivirus; 1 week to transduce cells and perform antibiotic selection; 1-2 weeks to image the cells, read out the barcodes, and analyze the data (this step may take longer depending on the size of the library). Details of each step are provided below.

### Library sequence design

The small and large protein sequence libraries were designed to test previously reported trends as well as to more systematically assess the impacts of amino acid composition and patterning across diverse sequence contexts. Complete details of the library design are provided in Supplementary Note 1.

### Pooled library cloning

DNA sequences were synthesized as 300 nucleotide oligonucleotide pools by Twist Bioscience (all sequences provided in Supplementary Data 1 and Supplementary Data 3). DNA was resuspended in water to a final concentration of 5 ng/μL. The DNA library was first amplified by PCR (0.3 μM forward and reverse primers, 0.04 ng/μL template oligonucleotide pool, 1X KAPA HiFi HotStart Ready Mix (Roche Cat. # KK2601)) with the following settings: 3 minutes denaturation at 95°C; then 14 cycles of 20 seconds denaturation at 98°C, 15 seconds annealing at 63°C, 20 seconds extension at 72°C; and finally, extension at 72°C for 1 minute. All primer sequences are provided in Supplementary Table 8. Reactions were cleaned up with a Qiagen MinElute column (Qiagen Cat. # 28006). Final concentration was measured by Qubit.

The resulting pool was then cloned into a lentiviral backbone plasmid (pGFP_1, pGFP_4, pGFP_6, pGFP_24, pSNAP_1, pSNAP_4, pSNAP_6, pSNAP_24, pCMV_GFP_24, or pCMV_SNAP_24) that contains from 5’ to 3’: a promoter (CMV or TRE), a filler region where the library will be cloned into, a nuclear localization signal, fluorescent protein, and optionally, an oligomerization domain. The plasmids also contain a separate puromycin resistance cassette. First, the backbone plasmid was digested with Esp3I (New England Biolabs Cat. # R0734L) and gel extracted. The library was then cloned into this digested backbone plasmid in a 120 μL (large pools) Golden Gate reaction (12 μL 10X Tango Buffer (Thermo Fisher Scientific Cat. # BY5), 12 μL 10mM DTT, 12 μL 10 mM ATP (New England Biolabs Cat. # P0756S), 2.4 μL Esp3I, 2.4 μL T7 ligase (New England Biolabs Cat. # M0318S), 0.081 pmol digested backbone plasmid, 0.162 pmol insert library). For the small pools, reactions were scaled down to 16 μL and the backbone plasmid to insert ratio was adjusted to 5:1. The reactions were run for 15 cycles of 5 minutes at 37°C and 5 minutes at 20°C, then cleaned up twice, sequentially, with Qiagen MinElute columns (Qiagen Cat. # 28006), and finally eluted in 10 μL of water. For the large pools, 5 μL of this final reaction was then electroporated into 25 μL of Endura electrocompetent cells (Lucigen Cat. # 60242-2), following the manufacturer’s instructions. For the small pools, the full 10 μL of the purified Golden Gate reaction was electroporated into 25 μL of Endura electrocompetent cells. Bacterial cultures were allowed to recover for 1 hour at 37°C, shaking at 250rpm. Serial dilutions were then plated onto LB-ampicillin plates for colony counting, to ensure at least 300x library coverage. The remainder of the culture was plated on a large 10’’x10’’ LB-ampicillin plate, then incubated overnight at 37°C. Bacterial colonies were then scraped off the plates and mini-prepped (ZymoPURE Plasmid MiniPrep kit, Zymo Research Cat. # D4210). To assess the quality of the resulting library, inserts from the resulting plasmid pool were amplified in two sequential PCR reactions to add Illumina sequencing adapters, including indices (PCR 1, 25 μL reaction: 12.5 μL NEBNext 2X PCR mastermix (New England Biolabs Cat. # M0541S), 1.25 μL 10 μM forward primer, 1.25 μL 10 μM reverse primer, 10 ng template plasmid, 9 μL water; PCR 2, 100 μL reaction: 50 μL NEBNext 2X PCR mastermix, 5 μL 10μM forward primer, 5 μL 10μM reverse primer, 1 μL PCR 1, 38 μL water). PCR reactions were run on a thermocycler with 30 seconds initial denaturation, then cycles of 10 seconds denaturation at 98°C, 30 seconds of annealing at 72°C, 30 seconds of extension at 72°C, and finally, extension for 2 minutes at 72°C. PCR 1 was run for 12 cycles and PCR 2 was run for 14 cycles. PCR 2 was then gel extracted. Libraries were sequenced on an Illumina MiSeq using a single forward read of 250 cycles and 8 cycle index reads.

### Arrayed cloning

DNA sequences were synthesized as eBlocks (Integrated DNA Technologies), then cloned individually by Golden Gate assembly, as described above for pooled libraries. All sequences are provided in Supplementary Table 8. All plasmids were verified by full plasmid sequencing.

### Cell culture

All cells were cultured at 37°C and 5% CO_2_ in DMEM, high glucose, supplemented with GlutaMAX (Life Technologies Cat. # 10569044) and 10% FBS (Takara Bio Cat. # 631107) and 100 U/mL penicillin-streptomycin (Thermo Fisher Scientific Cat. # 15140122). All experiments were performed in HeLa cells modified to express TetR and Cas9 (a gift from Paul Blainey’s lab) or U2OS cells (ATCC HTB-96). HEK293FT cells (Thermo Fisher Cat # R70007) were used to produce lentivirus. Cells were not authenticated.

### Lentivirus production

HEK293FT cells were seeded at approximately 60% density, in packaging media (Opti-MEM I reduced serum medium (Thermo Fisher Scientific Cat. # 31985070) supplemented with 5% FBS and 200 μM sodium pyruvate (Thermo Fisher Scientific Cat. # 11360070)). After one day, cells were transfected with 190 ng pMD2.G (Addgene #12259), 375 ng psPAX2 (Addgene #12260), 500 ng of the transfer plasmid, 2.5 μL Lipofectamine 3000 (Thermo Fisher Scientific Cat. #L3000015), 2.2 μL P3000 Enhancer Reagent, and 180 μL Opti-MEM I, per well of a 12-well plate (quantities were scaled linearly with the surface area of the dish for other dish sizes). Media was exchanged after 4 hours. Viral supernatant was collected after 24 hours and again after 48 hours, then filtered through 0.45 μm filters (96-well plate format: VWR Cat. # 97052-126; individual syringe format: VWR Cat. # 28143-312).

### Lentivirus transduction and selection

Cells were transduced by adding viral supernatant supplemented with polybrene (final concentration 8 μg/mL; Sigma-Aldrich Cat. # TR-1003-G). Cells were transduced at less than 10% multiplicity of infection. Sufficient cells numbers were used for at least 100x library coverage. Two to three days after transduction, cells were passaged into media containing puromycin (1 μg/mL; Life Technologies Cat. # A1113803). After two more days, cells were passaged again into media with puromycin and allowed to grow for two days.

### Small and large library CondenSeq experiments

HeLa cells, in sufficient numbers for at least 100x library coverage, were transduced with the appropriate lentiviral library and selected as described above. Approximately 40,000 cells were plated per well of a 24-well glass bottom plate (Cellvis Cat. # P24-1.5H-N) in media containing doxycycline to induce protein expression (final concentration 1μg/mL; Sigma-Aldrich Cat. # D9891-1G) and puromycin (final concentration 0.1 μg/mL; Life Technologies Cat. # A1113803). This specific brand of glass plates was critical for ensuring that the glass bottom did not detach from the plastic upper during subsequent *in situ* SBS. After two days, SNAP-tag libraries were labeled by exchanging media with 200 μL of freshly prepared 1μM labeling solution of SNAP-Cell Oregon Green (New England Biolabs Cat. # S9104S) (in normal culture media), incubating cells at 37°C, 5% CO_2_ for 30 minutes, then washing the cells three times with culture media, then incubating the cells in fresh media for an additional 30 minutes. All cells were then stained with Hoechst by exchanging media with Hoechst labeling solution (2μg/mL Hoechst 33342 (Thermo Fisher Scientific Cat. # H3570) in imaging media (DMEM no phenol (Thermo Fisher Scientific Cat. # 21063045) supplemented with 10% FBS and 100 U/mL penicillin-streptomycin (Thermo Fisher Scientific Cat. # 15140122)), incubating at 37°C, 5% CO_2_ for 5 minutes, then washing twice with imaging media. Finally, all media was exchanged for 500 μL imaging media. Cells were then imaged live, as described below. It took approximately 3.5 hours to image 8 wells of a 24-well plate. Immediately after imaging, cells were fixed and *in situ* SBS was performed, as described below. At least two independent biological replicates were performed for each screen.

### U2OS CondenSeq experiments

Experiments were performed as described above for HeLa cells, except that cells were transduced with libraries expressed from a CMV promoter (pCMV_GFP_24, pCMV_SNAP_24) and selected with puromycin, after transduction, for a total of 8 days. Additionally, because protein expression was constitutive, doxycycline was not added to the imaging media. Two independent biological replicates were performed.

### Live cell time-lapse imaging experiments

Cells were transduced and selected as described as above, then 30,000 cells were plated per well of a 24-well glass bottom plate (Cellvis Cat. # P24-1.5H-N) in media containing puromycin (0.1 μg/mL). After two days, cells were stained with Hoechst as described above, then protein expression was induced by adding imaging media containing 1 μg/mL doxycycline. Approximately 1 hour later, we started imaging. Images were taken at 48 time points, approximately every 35 minutes, over 28 hours. Immediately after imaging, cells were fixed and *in situ* SBS was performed, as described below. Two independent biological replicates were performed.

### *In situ* sequencing by synthesis (SBS)

*In situ* SBS^52, 53^ was performed as follows. All quantities listed here are for one well of a 24-well plate. Immediately following live-cell imaging, cells were fixed with 250 μL 4% paraformaldehyde (Electron Microscopy Sciences Cat. # 15710) in PBS for 30 minutes. Cells were then washed three times with PBS, and permeabilized with 250 μL 70% ethanol for 30 minutes. After permeabilization, ethanol was not immediately removed to avoid drying out the cells; rather, 250 μL of PBS with 0.05% Tween (Teknova Inc. Cat. # T0710) (PBS-T) was successively added and removed at least 5 times before the full volume was removed and cells were washed three times with PBS-T. 300 μL of reverse transcription mix (1X RevertAid RT buffer (Life Technologies Cat. # EP0452), 250μM dNTPs (New England Biolabs Cat. # N0447L), 0.2 mg/mL BSA (New England Biolabs Cat. # N0447L), 1 μM reverse transcription primer, 0.8 U/μL Ribolock RNase inhibitor (Life Technologies Cat. # EO0384), 4.8 U/μL RevertAid H minus (Life Technologies Cat. # EP0452)) was then added and the plate sealed well with an aluminum plate seal and incubated on a flat top heat block at 37°C overnight. The following day, cells were fixed again with 250 μL of 3% paraformaldehyde with 0.1% glutaraldehyde (Electron Microscopy Sciences Cat. # 16120) in PBS for 30 minutes. Cells were then washed 5 times with PBS-T, then 200 μL of gap-fill reaction mix was added (1X ampligase buffer (Biosearch Technologies Cat. # A1905B), 0.4 U/μL RNase H (Qiagen Beverly Cat. # Y9220L or New England Biolabs Cat. # M0297L), 0.2 mg/mL BSA (New England Biolabs Cat. # N0447L), 100 nM padlock primer, 0.02 U/μL TaqIT polymerase (Qiagen Beverly Cat. # P7620L), 0.5 U/μL ampligase (Biosearch Technologies Cat. # A3210K), 50 nM dNTPs (New England Biolabs Cat. # N0447L)). The plate was incubated at 37°C for 5 minutes, then at 45°C for 90 minutes on a flat top heat block. Cells were then washed three times with PBS-T, then 200 μL rolling circle amplification mix was added (1X phi29 buffer (Life Technologies Cat. # EP0094), 250 μM dNTPs (New England Biolabs Cat. # N0447L), 0.2 mg/mL BSA (New England Biolabs Cat. # N0447L), 5% glycerol, 1 U/μL phi29 DNA polymerase (Life Technologies Cat. # EP0094)). The plate was then incubated overnight on a flat top heat block at 30°C. Cells were then washed three times with PBS-T and then 200 μL of sequencing primer (1 μM primer in 2X SSC buffer (Life Technologies Cat. # 15557044)) was added and the plate was incubated at room temperature for 30 minutes. Cells were then washed three times with PBS-T.

For each cycle of SBS, 200 μL of Illumina MiSeq reagent #1 (incorporation mix; Illumina Cat. # MS-103-1001) was added to each well at room temperature (all SBS buffer exchanges were performed in a chemical fume hood), then the plate was moved to a flat-top heat block preheated to 60°C and incubated for 3 minutes. 500 μL of PR2 (Illumina Cat. # MS-103-1001) was then successively added and removed from each well at least 5 times before all liquid was quickly removed and replaced with fresh PR2. It was critical that the cells never dried out with incorporation mix on them. Cells were washed three times with PR2 at room temperature, then washed at least 5 times with PR2, incubating the plate at 60°C for 5 minutes for each wash. At least one long heated wash (10 minutes at 60°C) was performed. Washing for less time or at lower temperatures caused background signal to increase to problematic levels. Washing for very long times (more than 30 minutes), caused glass bottoms of plates to start to separate from the plastic uppers. Wash buffer was then removed and 500 μL of 200 ng/mL DAPI (Sigma-Aldrich Cat. # D9542-1MG) in 2X SSC was added. Cells were then imaged as described below. After imaging, 200 μL MiSeq reagent #4 (cleavage mix) was added and the plate was incubated at 60°C for 6 minutes. Cells were washed three times with PR2 at room temperature, then once at 60°C for one minute, then three more times at room temperature. The next cycle was then started by adding MiSeq reagent #1 (incorporation mix) again. 5 cycles of SBS were performed for the small pools and 8 cycles of SBS for the large pools.

### Fluorescence microscopy

All phenotype imaging was performed on an Opera Phenix High-Content Screening System (PerkinElmer) with a 40x water immersion objective in confocal mode. DAPI images were taken with 405 nm excitation at 60% power, a 435-480nm emission filter, and 40 ms exposure time. GFP/SNAP-tag images were taken with 488 nm excitation at 15% power, a 435-550nm emission filter, and 40 ms exposure time. For live-cell time lapse experiments, we collected images for eight z-slices, spaced 1 μm apart, at each position. For all other screens, we collected images at a single z-plane. The average intensities computed from a single slice were strongly correlated with the average intensities over multiple planes (Pearson’s r = 0.99).

*In situ* SBS imaging was performed on a Nikon Ti2-E inverted microscope with automated stage control, a Photometrics Iris 9 camera, and fluorescence illumination with a Lumencor Celesta light engine coupled with a liquid light guide to a 0.9 mm^2^ illumination at the rear port of the scope. DAPI images were taken with 405 nm laser excitation, 10% power, 70 ms exposure time, with a custom Chroma polychroic (ZT408/473/545/635/750/RPC_UF2), and custom Magnatron penta barrier filter for Celesta (ZET408/473/545/635/750m). MiSeq G images were taken with 518 nm laser excitation, 25% laser power, 200 ms exposure time, with a Chroma T525LPXR dichroic, and Chroma ET560/40x filter. MiSeq T images were taken with 545 nm laser excitation, 22% laser power, 200 ms exposure time, with a custom Chroma polychroic (ZT408/473/545/635/750/RPC_UF2), and custom Magnatron penta barrier filter for Celesta (ZET408/473/545/635/750m). MiSeq A images were taken with 640 nm laser excitation, 19% laser power, 200 ms exposure time, with a custom Chroma polychroic (ZT408/473/545/635/750/RPC_UF2), and custom Magnatron penta barrier filter for Celesta (ZET408/473/545/635/750m). MiSeq C images were taken with 640 nm laser excitation, 30% laser power, 200 ms exposure time, with a Chroma T647ILPXR dichroic, and Chroma ET720/60m filter. For the final SBS cycle, images were also collected in the GFP channel (475 nm laser excitation, 15%/4% (SNAP-tag/GFP) laser power, 300/100 ms (SNAP-tag/GFP) exposure time, green filter) to assist with matching SBS images to phenotype images. All images were collected with 2x2 pixel binning, with a 10x CFI Plan Apo Lambda objective (Nikon MRD00105). Image acquisition was automated with Nikon NIS-Elements AR software (version 5.21.03).

### Arrayed endogenous condensate colocalization experiments

Cells expressing test protein constructs were prepared in an arrayed format, then imaged live and then again after fixation and antibody staining to assess colocalization with several endogenous nuclear condensates. Complete experimental details are provided in Supplementary Note 1.

### GFP concentration calibration

GFP was purified and quantified to create a standard curve for measuring approximate GFP concentration from CondenSeq phenotype images. Complete details are provided in Supplementary Note 1.

### Western blot for FTH1 oligomerization domain

Levels of endogenous FTH1 were compared to library expression levels by western blotting. Complete details are provided in Supplementary Note 1.

### Image analysis

Images were analyzed to read out barcodes, assign barcodes to phenotype images, detect and quantify condensates in phenotype images. Complete details of the image analysis methods are provided in Supplementary Note 1.

### Defining concentration bins

Three concentration bins (low, medium, and high) were defined for analysis. The large pool data was used for calculating the bounds of these bins. For each barcode, the 10^th^, 25^th^, 50^th^, 75^th^, and 90^th^ percentiles of the mean GFP/SNAP-tag intensity values were calculated. The medium bin was defined as the mean of the 25^th^ percentile values to the mean of the 75^th^ percentile values. The low bin was defined as the mean of the 10^th^ percentile values to the median of the mean of the 10^th^ and the mean of the 90^th^ percentile values. The high bin was defined as the median of the mean of the 10^th^ and the mean of the 90^th^ percentile values to the mean of the 90^th^ percentile values. A protein sequence was included for analysis in given a concentration bin if at least 25% of all of the cells expressing that sequence had total concentrations within that particular bin and if there were at least 30 cells within the bin.

### Calculating sequence features

For each test protein sequence, the fraction of each amino acid was defined as the number of instances of that amino acid divided by the total number of amino acids (here, always 66). For ratios of groups of amino acids, for example “fraction R/RK”, was defined as the number of R residues divided by the total number of R and K residues. Sequences that do not contain any R or K values do not have a defined fraction R/RK. The mean hydropathy was computed using the Kyte-Doolittle index with localcider^74^. The “fraction disordered” score was calculated as the fraction of residues with disorder scores above 0.5, as predicted by IUPred2A^75^. The “average disorder” score was calculated as the mean predicted disorder score (IUPred2A) over all positions in the sequence. The NCPR was calculated as the sum of the R and K residues minus the sum of the D and E residues in the test protein, divided by the test protein sequence length. The FCR was calculated as the fraction of R, K, D, and E residues out of the total number of amino acids in the sequence. Patterning parameter values were calculated using NARDINI^76^. The software was modified so that values were calculated only if the sequence contained at least 5% of the patterned amino acid(s) (> 3 amino acids for all sequences here). All sequence features were computed for the test proteins only (not including oligomerization domains or fluorescent proteins).

To quantify correlation between sequence features and condensate formation, we computed Pearson’s correlation coefficients between sequence features and *f_condensates_*. p-values were corrected for multiple comparisons by applying the Bonferroni correction. Correlation coefficients were computed independently for GFP and SNAP-tag sequences.

### Classifying nucleolus- and chromatin-localizing sequences

Two separate classifiers (one for GFP and one for SNAP-tag fused sequences) were trained that take an image as input and predict whether the fluorescently tagged protein sequence in the image localizes to the nucleolus or chromatin, forms other condensates, no condensates, or is expressed poorly (for GFP sequences only). Complete details of these classifiers are described in Supplementary Note 1.

### Identifying sequences with concentration-buffering capacity

Sequences with concentration-buffering capacity (constant sequences) were first identified for GFP and SNAP-tag fusions independently as sequences that formed condensates within the high concentration bin (*f_condensates_* > 0.3), were not nucleolus- or chromatin-localizing (fractions nucleolus- or chromatin-localizing < 0.3), and had constant dilute phase concentration. To assess whether the dilute phase concentration was constant, the high concentration bin was partitioned into three equally spaced concentration bins and p-values were computed for the significance of differences in dilute phase concentrations between the three bins (two-sided t-test). If all three p-values were above 0.01, and if the standard deviation of the dilute intensity over the entire intensity range was less than 120 a.u. (GFP) and 25 a.u. (SNAP-tag), it was concluded that there was no significant change in dilute phase concentration as concentration increased. A sequence was classified with concentration-buffering capacity if, in addition, the total condensate area increased as the protein concentration increased. To check this, for each sequence, the total condensate area values for a given protein sequence was compared within two concentration bins (for GFP: 0.3 – 2.2 μM, 2.2 – 4.0 μM; for SNAP-tag intensity: 150 – 238 a.u., 238 – 325 a.u.). If the condensate area values between these bins increased significantly (two-sided t-test, p-value < 0.05), it was determined that the total condensate area increased as protein concentration increased. The final set of constant sequences contained only sequences that were identified as constant for both GFP and SNAP-tag. The “other sequences” were defined as sequences that were not within the final set of constant sequences, formed condensates (*f_condensates_* > 0.3), and were not nucleolus- or chromatin-localizing (fraction nucleolus-localizing < 0.3, fraction chromatin-localizing < 0.3).

### Classifying prion-like sequences

PLAAC was used to predict how prion-like sequences were^77^, using the webserver (http://plaac.wi.mit.edu/) with the core length set to 66. Prion-like sequences were defined as those with VITmaxrun of greater than 20 amino acids. Non-prion-like sequences were defined as those with VITmaxrun values of less than or equal to 20 amino acids.

### Calculating the normalized effect of single amino acid mutations

A normalized effect per single amino acid substitution was defined as the average change per single amino acid substitution for a given mutation type divided by the absolute value of the average change per single amino acid substitution over all mutation types for both GFP and SNAP-tag fusions. Normalized effects were only computed and plotted for mutation types for which there were mutants for at least 10 distinct base sequences.

### *In vitro* C_sat_ predictions

*In vitro* C_sat_ values for all test protein sequences in the small sequence library were predicted using a recently developed predictor^44^. The nuclear localization sequence was included and each sequence was concatenated 24 times to simulate valence = 24.

### Consistency score calculation

Consistency scores were computed to quantify the consistency of the effects of mutation types on condensate formation across many sequence contexts. Complete details of these scores are provided in Supplementary Note 1.

### Predicting homotypic and heterotypic interactions with FINCHES

All IDRs within the human proteome were identified as described in “Analysis of library composition” in Supplementary Note 1. FINCHES^45^ was used to predict the favorability of interactions between each human IDR longer than 100 amino acids (a total of 4,057 sequences) and each test protein sequence in the large sequence library (14,622 sequences), a total of 59,321,454 possible interactions. The favorability of each interaction is described by a single interaction parameter, ε, for which negative values are attractive, positive values are repulsive. The Mpipi force field was used for all calculations^26^. Ward’s method for hierarchical clustering was used to cluster the interaction profiles for the test protein sequences into 10 clusters (this number of clusters was manually selected, by testing varying numbers and assessing the resulting extent of decomposition). For comparisons of sequences that form condensates versus those that do not, condensate forming sequences were defined as those with *f_condensates_* > 0.3. For each test protein, the favorability of homotypic interaction (the interaction between two copies of the test protein) was also computed. We also used FINCHES to analyze possible cation-π interactions; complete details are provided in Supplementary Note 1.

## Extended Data

**Extended Data Table 1.** Reproducibility and numbers of barcode and protein sequences for small libraries.

Library	r^2^ between replicates (over low/med/high concentration bins))	# barcodes (≥ 20 cells)	Median number of cells	Total number of cells	Barcodes in low concentration bin	Barcodes in medium concentration bin	Barcodes in high concentration bin
GFP, live cell time-lapse, valence 24	0.96	99	224	28,119	99	99	99
GFP, valence 1	0.94	99	1341	151,651	96	66	40
GFP, valence 4	0.91	99	1314	158,214	96	72	44
GFP, valence 6	0.85	99	1313.5	166,347	70	64	44
GFP, valence 24	0.85	99	1132	147,610	85	78	50
SNAP, valence 1	0.93	99	1187	143,660	98	75	51
SNAP, valence 4	0.90	99	1921.5	220,736	76	89	75
SNAP, valence 6	0.88	99	1695.5	191,000	70	86	82
SNAP, valence 24	0.88	99	1392	171,110	48	74	80
U2OS GFP, valence 24	0.95	99	205	48,947	78	54	24
U2OS SNAP, valence 24	0.95	99	114	21,181	35	20	18

**Extended Data Table 2.** Reproducibility between libraries.

Library 1	Library 2	r^2^ (*f_condensates_*)	r^2^ (fraction signal in condensates)	Barcodes/protein sequences in low concentration bin	Barcodes/protein sequences in medium concentration bin	Barcodes/protein sequences in high concentration bin	Total unique protein sequences in low, medium, high bins
GFP sublibrary 1	GFP sublibrary 2	0.95	0.96	49	44	39	73
GFP sublibrary 1	GFP sublibrary 3	0.92	0.96	53	44	37	79
GFP sublibrary 2	GFP sublibrary 3	0.95	0.90	57	53	43	89
SNAP sublibrary 1	SNAP sublibary 2	0.96	0.97	36	58	83	97
SNAP sublibrary 1	SNAP sublibrary 3	0.84	0.93	38	50	64	89
SNAP sublibrary 2	SNAP sublibrary 3	0.88	0.94	34	61	66	92
GFP small pool	GFP sublibrary 3	0.93	0.93	67	60	37	83
GFP small pool	GFP sublibrary 2	0.94	0.91	53	40	28	72
GFP small pool	GFP sublibrary 1	0.93	0.91	42	30	22	57
SNAP small pool	SNAP sublibrary 3	0.89	0.95	35	54	54	78
SNAP small pool	SNAP sublibrary 2	0.93	0.96	32	60	67	84
SNAP small pool	SNAP sublibrary 1	0.92	0.96	31	50	55	70

**Extended Data Table 3.** Reproducibility and numbers of barcodes and protein sequences for large libraries.

Library	r^2^ between replicates: f_condensates_	r^2^ replicates: fraction signal in condensates	# barcodes (>=20 cells)	Median number of cells	Total number of cells	Barcodes (protein sequences) in low concentration bin	Barcodes (protein sequences) in medium concentration bin	Barcodes (protein sequences) in high concentration bin	Total unique barcodes in low, medium, high bins (protein sequences)
GFP sublibrary 1	0.92	0.94	5181	196	1188294	2685 (2684)	2171 (2169)	1955 (1953)	3643 (3640)
GFP sublibrary 2	0.92	0.95	4816	247	1380918	2832 (2829)	2295 (2293)	2010 (2007)	3747 (3741)
GFP sublibrary 3	0.93	0.94	4945	181	1082580	3456 (3410)	2976 (2923)	2156 (2109)	4274 (4206)
SNAP sublibrary 1	0.92	0.94	5188	253	1513203	2209 (2209)	2707 (2705)	3717 (3711)	4677 (4671)
SNAP sublibrary 2	0.93	0.95	4819	273.5	1515508	2278 (2276)	3169 (3166)	3578 (3574)	4565 (4560)
SNAP sublibrary 3	0.91, 0.87, 0.92	0.91, 0.93, 0.95	4939	189	1142342	1797 (1776)	2261 (2228)	2749 (2693)	3928 (3859)

**Extended Data Table 4.** Number of protein sequences in each dataset.

Library	Protein seqs in low concentration bin	Protein seqs in medium concentration bin	Protein seqs in high concentration bin	Total unique protein sequences in low, medium, & high bins
GFP natural protein sequence fragment library	1069	685	504	1280
SNAP natural protein sequence fragment library	755	765	904	1392
GFP compositional variation library	5407	4596	3744	7083
SNAP compositional variation library	3809	5234	6392	8163
GFP patterning variation library	2228	1743	1280	2767
SNAP patterning variation library	1576	1856	2179	3044

## Supplementary Material

Supplementary Data 1

Supplementary Data 2

Supplementary Data 3

Supplementary Tables Combined

Supplementary Results, Discussion, Notes, Figures

## Data Availability

Images have been deposited to the Bioimage Archive^78^ (accession number S-BIAD1738). Processed data for all protein sequences is available in Supplementary Data 1, Supplementary Data 2, and Supplementary Data 3. FINCHES predictions for the CondenSeq large library sequences are available on Zenodo (DOI: 10.5281/zenodo.15098929). Previously published databases that we used for sequence design or analysis are publicly available: MobiDB (https://mobidb.org/), LLPSDB (http://bio-comp.org.cn/llpsdb/home.html), Disprot (https://disprot.org/), Phasepro (https://phasepro.elte.hu/).

## Code availability

Code for analyzing SBS data is available at https://github.com/kkappel1/OpticalPooledScreens2023 (this is a slightly modified version of previously published code^52^). Additional image analysis code, including examples, is available at https://github.com/kkappel1/ops_analysis.
