# Supplementary material for: Characterizing protein sequence determinants of nuclear condensates by high-throughput pooled imaging with CondenSeq
Source: Nat Methods. Author manuscript; Available in PMC 2025 Sep 24. (PMC12458759; doi:10.1038/s41592-025-02726-y)
Supplement: Supplementary Results, Discussion, Notes, Figures [file NIHMS2109557-supplement-Supplementary_Results__Discussion__Notes__Figures.pdf]

Supplementary Results

Applying CondenSeq to a library of longer protein sequences

One of the interesting future applications of CondenSeq will be to decipher the sequence features that affect condensate formation in longer or full-length protein sequences. Therefore, to test whether CondenSeq can be used to characterize longer protein sequences, we performed a preliminary proof-of-principle experiment applying CondenSeq to a library of 69 longer protein sequences, ranging in length from 85 to 296 amino acids ("long sequence library"). The test proteins were fused to GFP or SNAP-tag and to FTH1. This library contains longer disordered regions from 36 proteins, selected in the same manner in which the natural protein sequences in our small sequence library were selected (**Supplementary Note 1**, **Supplementary Data 2**). Many of these sequences are extended fragments from our natural sequence library (e.g., the natural protein sequence library may contain a protein sequence fragment that spans from residue 1 to 66 of full-length protein A, and the long sequence library may then contain a protein sequence fragment that spans from residue 1 to 200 of full-length protein A). The rest of the library contains variants of these longer natural sequence fragments that vary sequence features that we identified in our other libraries to affect condensate formation (especially charged and aromatic residues).

Our experiments were reproducible between independent replicates (**Supplementary Figure 3A**). By comparing analogous short versus long designed sequence variants, we found that sequence variants had similar impacts on the propensities of short and long sequences to form condensates (**Supplementary Figure 3B**). By comparing short sequences to their extended versions from the long sequence library (i.e., the short sequence is a subset of the corresponding long sequence), we found that, in most cases, the longer sequences had greater than or equal propensity to form condensates (**Supplementary Figure 3C**). We also compared multiple different short sequence subsets of our longer protein sequences and found that many long protein sequences with high propensities to form condensates ($f_{condensates} > 0.8$) contained regions (short sequence subsets) with a wide range of propensities to form condensates (**Supplementary Figure 3D**). These results are consistent with the idea that the overall sequence composition and features of full-length proteins govern their propensity to form condensates. We are excited to use CondenSeq to more systematically explore this idea, together with the role that folded domains play in regulating nuclear condensate formation, in future work.

Increasing valence promotes condensate formation

Prior work has demonstrated that increasing valence promotes condensate formation[1-4]. Our initial tests with valence = 1 and valence = 24 followed this trend. To more systematically test if CondenSeq recapitulates this trend across diverse protein sequences in cells, we characterized our small sequence library of 99 test protein sequences fused to two additional oligomerization domains forming 4-mers and 6-mers, in addition to the FTH1 24-mer domain and no oligomerization domain (valence = 1) (**Extended Data Fig. 3A**, **Methods**, **Supplementary Note 1**). To control for the impact of the fluorescent protein fusion, we also repeated all experiments with the libraries fused to SNAP-tag instead of GFP (**Supplementary Figure 5C**). We confirmed that all

oligomerization domain-fluorescent protein fusions lacking a fused test protein do not form condensates (example cell images shown in **Supplementary Figure 4B**). For each library, we imaged live cells two days after protein expression induction, followed immediately by *in situ* SBS (**Methods**). Because condensate formation is concentration-dependent, we separately analyzed cells with test protein expression in specific narrow concentration bins (**Methods**; GFP: 0.3-2.2μM (low), 1.0-3.1μM (medium), and 2.2-4.0μM (high); **Extended Data Table 1**). Results from the valence=24 GFP library agreed well with the live cell time-lapse results (Pearson's r^2 = 0.85 and 0.87 for low and medium bins, respectively).

As valence increased, more test protein sequences formed condensates (GFP fusions: **Extended Data Fig. 3B**; SNAP-tag fusions: **Supplementary Figure 6A**). Each protein sequence exhibited a "critical valence," such that if the protein sequence formed condensates at a given valence, it also formed condensates at all higher valences (**Extended Data Fig. 3C**, **Supplementary Figure 6B**).

It is important to note that $f_{condensates}$ values do not necessarily scale linearly between different valences. For example, the magnitude of the change in $f_{condensates}$ from valence 1 to 2 may not be the same as the change in $f_{condensates}$ from valence 2 to 3. The values also may not scale linearly between different sequences; for example, the change in $f_{condensates}$ from valence 1 to valence 4 for one test protein sequence may be different than that for another test protein sequence. This non-linearity is expected for two main reasons. First, increases in valence can introduce sequence-specific cooperative interactions, with non-linear behavior. Second, because our $f_{condensates}$ measurements are bounded between 0 and 1, we will only detect changes in condensation propensity for sequences with C_{thresh} values that span the measured concentration range. To assess whether the relationship is indeed non-linear, we compared $f_{condensates}$ values between each pair of valences for all test protein sequences. While the values are correlated (Pearson's r between 0.25 and 0.81) and the relationship is roughly monotonously non-negative, it is clearly non-linear (**Supplementary Figure 6C**). This means that we cannot, for example, use the valence = 24 results to quantitatively infer $f_{condensates}$ values for the corresponding test protein sequences at valence = 1. However, we confirmed qualitative agreement between the sequence trends for different valences, which suggests that we can use results from one valence to qualitatively understand sequence features that impact condensate formation at another valence (**Supplementary Figure 6D**-**E**).

CondenSeq reproducibility

Several controls confirm the reproducibility of our large sequence library results. First, we included a small common set of sequences in each sub-library, and confirmed their reproducibility (**Extended Data Table 2**). Second, we tested identical protein sequences with different barcodes and confirmed that the results were consistent (**Supplementary Figure 7D**). Third, we confirmed that the results for sequences shared by the small and large sequence library were consistent (**Extended Data Table 2**). Fourth, to test for the impact of fusing the sequences to GFP, we repeated all of our experiments for the entire large sequence library with the test protein sequences fused to an orthogonal fluorescent protein, SNAP-tag. Test protein sequences fused to SNAP-tag consistently formed

condensates more readily than sequences fused to GFP (**Supplementary Figure 7E**), however, trends in the sequence features that affected condensate formation were highly consistent between the GFP and SNAP-tag fusions (including all trends described in the main text results). Indeed, we compared the impacts that sequence features had on condensate formation (Cohen's d) for sequences fused to GFP versus SNAP-tag, and found that they were highly correlated (Pearson's $r = 0.94$, **Supplementary Figure 7F**). Finally, all results were highly reproducible between independent biological replicates (**Fig. 2B**, **Extended Data Table 3**).

Phenotypes are consistent in a second cell type

To test the impact of cell type on condensate formation, we tested our small sequence library with valence of 24, fused to either GFP or SNAP-tag in U2OS cells (human bone osteosarcoma epithelial cells). We collected data for a total of 48,947 and 21,181 cells, with median cell counts per test protein of 205 and 114 cells, for GFP and SNAP-tag fusions, respectively (**Extended Data Table 1**). Results were reproducible with r^2 values between independent biological replicates of 0.95 and 0.95 for $f_{condensates}$ per protein sequence for GFP and SNAP-tag fusions. We observed strong agreement between the phenotypes in U2OS and HeLa cells, with $r^2 = 0.88$ and 0.95 for the fraction of the test protein found in condensates, for GFP and SNAP-tag fusions, respectively (**Supplementary Figure 8A-C**). This suggests that our results generalize beyond HeLa cells. We note, however, that this does not mean that this trend will hold for *all* cell types and in future work, it will be interesting to more comprehensively test the sequence-dependence of condensate formation across cell types.

Analysis of condensate-forming proteins with high NCPR

Proteins with higher NCPR are likely forming nuclear condensates through interactions with other molecules in the nucleus. To test this idea further, we used FINCHES[5] to predict interactions between our test proteins and all endogenous human IDRs longer than 100 amino acids (4,057 sequences). We then counted the total number of predicted possible attractive interactions for each test protein sequence with high NCPR (> 0.05), and found that the sequences that form condensates have, on average, a significantly higher number than those that do not form condensates (mean for sequences that do not form condensates = 123.7, mean for sequences that do form condensates = 411.4; $p < 0.005$; **Supplementary Figure 10A**). This supports the idea that heterotypic interactions are important for condensate formation for sequences with high NCPR.

We also hypothesized that favorable cation-π interactions may contribute to the propensity of proteins with higher NCPR to form condensates. To test this idea, we used FINCHES[5] to predict maps of homotypic interactions within each test protein sequence ("homotypic intermaps"). This enables identification of sub-regions within each test protein sequence that could interact favorably. We performed the analysis with a sliding window size of 11 residues, for every 11-residue window within each sequence. We then defined cation-π interactions as those between a window with a cation at the center (arginine or lysine) and a window with an aromatic residue at the center (phenylalanine, tyrosine, or tryptophan). For each test protein sequence, we counted the total number of possible attractive cation-π interactions (interaction parameter $\varepsilon < 0$). For test protein

sequences with high NCPR (> 0.05), we found that test protein sequences that form condensates have, on average, significantly more possible attractive cation-π interactions than test protein sequences that do not form condensates (mean for sequences that do not form condensates = 18.0, mean for sequences that do form condensates = 42.1; $p < 0.005$; **Supplementary Figure 10B**). We note that these analyses have limitations: FINCHES predicts the favorability of all possible interactions, and does not imply that all of these interactions are actually occurring. Still, these trends across large groups of protein sequences suggest that cation-π interactions may indeed promote condensate formation, consistent with several previous studies[2, 6].

Phosphorylation may impact nuclear condensate formation

Our observation that charge is a critical parameter impacting nuclear condensate formation suggests that phosphorylation and other post-translational modifications may play important roles in regulating nuclear condensates. We first asked whether higher NCPR drives condensate formation for sequences that do not contain commonly phosphorylatable residues. Indeed, we observed a significant positive correlation between NCPR and $f_{condensates}$ (Pearson's r = 0.71, p<0.005) (**Supplementary Figure 11A**), suggesting that higher positive NCPR drives condensate formation, even for sequences that cannot be phosphorylated. To get more insight into the impact that phosphorylation may have on condensate formation, we examined a set of phosphomimetic mutations within our library. Many, but not all, of the mutations decreased condensate formation (**Supplementary Figure 11B**). This suggests that phosphorylation can influence condensate formation, and that it would be worth investigating these effects further, as well as the roles that other types of post-translational modifications may play, in future work.

Direct mutations reveal differences between chemically similar amino acids

To directly assess the relative impacts of different amino acid types, we made a series of mutations in which one amino acid type is mutated to another. First, we tested arginine to lysine and lysine to arginine mutations and found that arginine more strongly promotes condensate formation than lysine, suggesting that the unique chemical characteristics of arginine, beyond simple positive charge, are contributing to condensate formation (**Fig. 3C, Supplementary Figure 12C**). In contrast, mutating glutamate into aspartate or aspartate into glutamate had no significant impact. We tested the relative impacts of all aromatic amino acids and found that tryptophan more strongly promotes condensate formation than phenylalanine and tyrosine. Lysine and arginine both consistently promote condensate formation more strongly than glutamine, again emphasizing the importance of net charge. Mutations that increase or decrease the overall hydropathy of a sequence have a wide range of positive and negative effects on condensate formation, with an average effect close to zero, suggesting that hydropathy is not a consistent driver of condensate formation. Looking more specifically into different types of hydrophobic amino acids, by mutating I/L/V residues to aromatic residues and vice versa, we found that aromatic residues promote condensate formation more strongly than I/L/V. Several other mutation types had little average impact, suggesting roughly equivalent impacts on condensate formation: N to Q, Q to N, aromatic to R, R to aromatic, G to S, and S to G.

We then asked how changing the fraction of charged residues (FCR), without changing the NCPR, affects condensates, by adding or removing equal numbers of positively and negatively charged residues. Overall, increasing or decreasing the FCR does not cause a consistent change in condensate formation (**Fig. 3C, Supplementary Figure 12C**). However, adding lysine together with aspartate and glutamate tended to slightly decrease condensate formation while removing lysine together with aspartate or glutamate tended to cause a slight increase in condensate formation on average. There is no significant or consistent trend for adding or removing arginine together with glutamate or aspartate, in agreement with the observation that arginine, aspartate, and glutamate tend to have similar impacts on condensate formation (**Fig. 3D**).

Amino acid patterning contributes to condensate formation
We designed our patterning variation sequence set to specifically test for the contribution of amino acid patterning. We described and quantified the amino acid patterning within these sequences using patterning z-scores, calculated with NARDINI[7], where higher positive z-scores indicate more clustering of the particular amino acid type(s) within the linear protein sequence, and lower negative z-scores indicate well-mixed sequences (**Extended Data Fig. 4A**). This synthetic sequence set contained 2,174 scrambled versions of the base sequences above, and 1,422 scrambled versions of sequence variants, that specifically increase (minimum NARDINI z-score = 2.9), decrease (maximum NARDINI z-score = -1.6), or maintain the patterning of positively charged (Ω_{+}), negatively charged (Ω_{-}), positively *vs.* negatively charged (δ_{+-}), aromatic (Ω_{FWY}), hydrophobic (Ω_{ILMV}), or polar (Ω_{STNQCH}) amino acids (**Extended Data Fig. 2, Methods**, **Supplementary Note 1**). We obtained high quality data in at least one of our three defined concentration bins for 2,767 sequences (**Extended Data Table 4**).

Scrambling the base sequences (fragments of natural proteins) caused changes in condensate formation, indicating that amino acid patterning does play a role (**Extended Data Fig. 4B**). If a specific amino acid sequence within a base sequence is important for condensate formation (e.g., a specific amino acid motif), we would expect scrambled sequences that disrupt this motif to have systematically different $f_{condensates}$ values than the base sequence. To quantify differences between base sequences and scrambled variants, we computed the probability of observing the $f_{condensates}$ value for each base sequence, given the distribution of $f_{condensates}$ values for all of the scrambled variants of that base sequence (**Extended Data Fig. 4B**: red lines denote $f_{condensates}$ values for base sequences, black dots represent $f_{condensates}$ values for scrambled variants). A low probability would indicate that the $f_{condensates}$ value for the base sequence is unlikely to have come from the distribution of $f_{condensates}$ values for the scrambled variants — i.e., the base sequence is significantly different from the scrambled variants, suggesting that something about the specific amino acid sequence within the base sequence is important for condensate formation. Out of 57 base sequences for which we had data for at least five scrambled variants in the medium GFP concentration bin, five base sequences had probabilities less than 1% (smoothed empirical cumulative distribution function (CDF) test; **Supplementary Note 1**). One (RBM14) had an $f_{condensates}$ value less than the $f_{condensates}$ values for all of the scrambled variants, suggesting that the specific ordering of amino acids within this base sequence may be important for lowered propensity to form

condensates. The remaining four had $f_{condensates}$ values above all of the respective scrambled variants — scrambling these base sequences disrupted condensate formation. Each of these four proteins contained a specific sequence motif: DYRK1A (poly-histidine tract), SYN1 (poly-proline tract), NAB3 (poly-proline and poly-glutamine tracts), and NUP100 (asparagine-serine repeats tract). The $f_{condensates}$ values for the remaining 52 base sequences were not significantly different than the values for their scrambled variants (p > 0.01, smoothed empirical CDF test). Thus, for these sequences, the exact amino acid ordering within the base sequence is not a major driver of condensate formation. We observed similar trends for other concentration bins and for SNAP-tag fusions (**Supplementary Figure 13A**).

To test the impact of each patterning parameter individually, we compared scrambled sequences with high (z-score $>$ 2.0) or low (z-score $<$ -2.0) patterning parameters to designed "unpatterned sequences," compositionally identical variants with patterning parameters near 0 (z-scores between -0.5 and 0.5), when varying only one patterning parameter at a time (**Supplementary Note 1**). When specifically considering unpatterned sequences that did not form condensates ($f_{condensates} < 0.3$), there was a statistically significant increase in condensate formation when the charge patterning (δ_{+-}) was increased (**Extended Data Fig. 4C**, **Supplementary Figure 13B**). We observed little change in condensate formation for decreasing clustering.

Across the entire large sequence library, there was a statistically significant, positive correlation between $f_{condensates}$ and both clustering of oppositely charged amino acids (δ_{+-}) (r=0.12) and clustering of negatively charged residues (Ω_{-}) (r=0.12), though the correlation is weaker than that between, for example, $f_{condensates}$ and NCPR (r=0.56) (**Extended Data Fig. 4D**, **Supplementary Figure 13C**, $p < 0.001$, Fisher r to z transformation, two-tailed z-test). We note that all of the proteins in our libraries are 66 amino acids long, and that patterning may be even more relevant for longer proteins. Altogether, these results suggested that amino acid patterning, particularly patterning of charged residues, contributes to condensate formation, but that it is not the sole determinant.

Assessing colocalization with endogenous nuclear condensates

We selected 59 sequences from our libraries, including 45 of the base sequences, and imaged cells expressing them fused to the 24-mer oligomerization domain and GFP or SNAP-tag in an arrayed format (to avoid crosstalk between antibody and SBS channels) after staining with antibodies for markers of nuclear speckles, nucleoli, and Cajal bodies, and with Hoechst to visualize chromatin. We also imaged cells prior to fixation and antibody staining to confirm that fixation did not impact the properties of any condensates formed by our fluorescently tagged sequences (**Supplementary Figure 14A**). We confirmed that the experiments were reproducible (**Supplementary Figure 14B**). Most condensate-forming sequences did not colocalize with any of the endogenous nuclear condensates (82% of sequences; example images shown in **Supplementary Figure 14C**; **Supplementary Note 1**); the remaining sequences colocalized with nucleolar, chromatin, or nuclear speckle markers (example images shown in **Supplementary**

Figure 14D and **Supplementary Figure 14E**; all colocalization data in **Supplementary Table 5**).

NCPR and aromatic residues impact condensate properties

For the condensate-forming sequences that neither localized to chromatin nor to nucleoli, we sought to determine whether any sequence features were correlated with three condensate properties: eccentricity, size, and number per cell. Several of the features that affect the propensity of a sequence to form condensates also impacted these condensate properties (**Supplementary Figure 15F**). Notably, NCPR impacted all three. Higher NCPR was associated with higher eccentricity and with a greater number of condensates per cell, while lower NCPR was associated with smaller average size per condensate. Additionally, higher fractions of aromatic residues were correlated with a higher number of condensates per cell. These results suggest that sequence features affect both the propensity to form condensates as well as the properties of those condensates.

Supplementary Discussion

Many previous studies have focused on discovering the protein sequence features that drive condensate formation[6, 8-26]. Each of these studies has typically focused on one model protein or one family of proteins and generally characterized just a handful of protein sequences, up to 166 protein sequences in one recent study[25]. Altogether, these studies have provided key insights into the roles that sequence features can play in condensate formation, but it was unclear to what extent these findings would generalize to new sequence contexts. Many of the sequence trends that we report here have previously been observed in other model systems — indeed, nearly every amino acid type has previously been shown to promote condensate formation in some context[26] (**Supplementary Table 4**). The power of our work is in revealing the consistency with which these sequence features promote condensate formation across many diverse sequence contexts. The unique scale of our CondenSeq assay enabled us to test nearly all previously reported trends with ~14,000 designed sequences, within a single study.

Still, CondenSeq has several important limitations:

CondenSeq is not an *in vitro* method and does not provide information about specific interactions with cellular factors

CondenSeq measurements should not be interpreted as measurements of *in vitro* condensate formation propensity. CondenSeq measurements are performed in the nuclear environment and capture the combined effects from the intrinsic biophysics of protein sequences and their heterogeneous interactions with cellular factors. These measurements do not provide information about specific interactions that test proteins may make with endogenous cellular factors nor do they reveal the other molecules that may be present in the condensates. We have begun to disentangle the impacts of this heterogeneity through our intermolecular chemical specificity analysis. Intriguingly, we find evidence to support the hypothesis that intermolecular chemical specificity can tune the sequence features that promote condensate formation. However, significant further work is needed to elucidate the actual interactions that each test protein makes within the cellular environment. This will require coupling CondenSeq with other approaches, likely including mass spectrometry-based methods[27, 28].

CondenSeq does not reveal the material properties nor dynamics of condensates

Deciphering the sequence-dependence of these relevant properties will require coupling CondenSeq with other approaches.

CondenSeq does not explicitly provide information about the role that post-translational modifications may play in condensate formation

These effects can be explored through sequence mutations with CondenSeq, but a detailed understanding of these relationships will also likely benefit from coupling this method with mass spectrometry-based approaches, and chemical and genetic perturbations.

CondenSeq is limited to studying *nuclear* condensate formation
CondenSeq is currently limited to studying nuclear condensate formation and the findings we report here are specific to the nuclear environment. Although it would be interesting to compare the sequence features that drive condensate formation in the nucleus versus the cytoplasm, adapting CondenSeq to study condensate formation in the cytoplasm would require additional steps to distinguish condensates from localization to cytoplasmic membrane-bound organelles. Simple immunostaining approaches may not be appropriate given the susceptibility of condensates to fixation artifacts[29]. Thus, additional live cell imaging strategies would need to be employed, which go beyond the scope of this study.

Cell type may impact the sequence determinants of condensate formation
Nearly all of our measurements here were performed in HeLa cells. For the small sequence library, we observed agreement between the results in HeLa cells and results in a second cell type, U2OS cells. However, this does not mean that this trend will hold for *all* cell types and in future work, it will be interesting to more comprehensively test the sequence-dependence of condensate formation across cell types.

Fluorescent protein fusions may impact condensate formation
CondenSeq measures condensate formation in live cells to avoid fixation artifacts, though it is important to note that fluorescent proteins can also affect condensate formation[30]. Here, we repeated all measurements with two distinct fluorescent protein fusions and confirmed that the trends we describe are consistent between the two, but it is possible that that other fluorescent protein fusions (or fusions to other protein sequences) may have different effects.

CondenSeq makes a tradeoff between throughput and measurement detail
CondenSeq experiments make a tradeoff between the number of sequences that can be characterized per sample and the richness of information per sequence. Live-cell time-lapse imaging can provide precise threshold concentrations, but requires long imaging times, thus limiting the numbers of cells and sequences that can be characterized. Similarly, the single- or few-plane imaging performed in most of our experiments, as opposed to time-consuming fine-scaled z-stacks, enables rapid characterization of large sequence libraries, but prohibits precise quantification of dense phase concentrations. Additionally, measuring condensate formation for sequences fused to multiple different oligomerization domains with different valences would enable full phase diagrams to be mapped, but is not yet practical for libraries of the scale studied here.

Supplementary Notes

Supplementary Note 1. Additional methodological information.

Library design

The small protein sequence library consisted of 45 fragments of natural protein sequences and 54 mutated variants of these sequences, for a total of 99 protein sequences. All protein sequences in the library were 66 amino acids in length. This length was chosen so that the full DNA sequence, including primer binding sites and sequences for cloning, would fit within the 300-nucleotide length limit for oligonucleotide pools that existed at the time our libraries were synthesized. The 45 fragments of natural proteins were manually selected from the subset of proteins in PhasePro[31] that have regions of less than 300 amino acids that are annotated to drive phase separation. Sequences that are annotated as partner dependent were excluded. 66 amino acid fragments were selected by finding the subsequence with the amino acid composition that most strongly correlated with the amino acid composition of the full region. The 54 mutated variants of these sequences were designed to test hypotheses about the sequence features that contribute to condensate formation based on previous studies[8, 9, 32-35].

The large protein sequence library consisted of the natural protein sequence fragment set, compositional variation sequence set, and patterning variation sequence set (**Extended Data Fig. 2A**). First, a set of 79 “base sequences” was selected for extensive mutagenesis. These sequences include 43 of the fragments of natural protein sequences and one designed variant from the small sequence library, for a total of 44 “Class 1 base sequences.” Another 35 fragments of natural protein sequences (“Class 2 base sequences”) were further selected from PhasePro, excluding protein sequences that were annotated as partner dependent. To this end, all protein regions annotated to drive phase separation were examined, and all possible 66 amino acid fragments with amino acid composition and dipeptide composition similarity (Pearson correlation coefficient (r^2)) to the Class 1 base sequences and to each other of less than 0.6 (“Class 3 sequences”) were identified. 35 of these sequences, including at most one sequence fragment per protein and prioritizing sequence fragments with the highest amino acid composition similarity to the full protein region as well as regions that were predicted to be more disordered.

The natural protein sequence fragment set includes: (1) all base sequences, (2) all remaining Class 3 sequences (283 sequences), (3) Class 4A sequences (519 sequences): fragments from disordered (as annotated by MobiDB[36]) sequences from LLPSDB[37] that were annotated either as phase separating or not phase separating with maximum amino acid composition and dipeptide correlation of 0.8 to each other and to all base sequences and all Class 3 sequences; and (4) Class 4B sequences (798 sequences): disordered regions (as annotated by MobiDB) from Disprot[38] (release 2022_03) with maximum amino acid composition and dipeptide correlation of 0.6 to each other and to all base sequences, Class 3 sequences, and Class 4A sequences.

The compositional variant library includes variants of the base sequences in which specific amino acids or groups of amino acids were mutated, including: 2,245 sequences

in which the numbers of specific amino acids (Y, F, G, S, P, N, Q, W, or H) were individually increased (by mutating G, A, or S to the amino acid; 2 variants per base sequence) or decreased (by mutating the amino acid to G or A; 2 variants per base sequence); 1,706 sequences in which the ratios of amino acid content (R vs K, D vs E, S vs G, N vs Q, Y vs F, F vs W, Y vs W, R vs Q, K vs Q) are varied by mutating one amino acid to another (3 variants per base sequence) and vice versa (3 variants per base sequence); 1,880 sequences in which the total numbers of groups of amino acids (I/L/M/V, R/K, D/E, G/S, Y/F/W) are increased (3 variants per base sequence) or decreased (3 variants per base sequence); 497 sequences in which ratios of groups of amino acids (I/L/M/V vs F/W/Y, F/W/Y vs R) are varied by mutating members of one group to the members of the other (3 variants) and vice versa (3 variants); 799 sequences in which the numbers of charged residues are increased (add 4 R, 4 K, 8 R, 8 K, 4D, 4E, 8D, 8E) or decreased (remove all R, all K, all E, or all D); 433 sequences in which the number of charged residues is increased (4 variants) or decreased (4 variants) without modifying the total charge; 474 sequences in which the predicted disorder, from IUPred2A[39], of the sequence is increased (3 variants) or decreased (3 variants); 474 sequences in which the hydrophobicity (Kyte-Doolittle index[40]) of the sequence is increased (3 variants) or decreased (3 variants); 947 sequences in which all instances of each amino acid are mutated individually to A or G.

The patterning variation sequence set includes variants of the base sequences and some compositional variants (add Y/F/W, add I/L/M/V, add equal numbers of R and E variants) in which the order of the amino acids is changed without modifying the composition. These variants include: 3,596 scrambled sequences in which the patterning of single groups of amino acids (E/D, R/K, F/W/Y, I/L/M/V, S/T/N/Q/C/H) or groups of amino acids relative to each other (R/K vs D/E) is increased, decreased, or kept similar. Patterning values were calculated with NARDINI[7].

The large protein sequence library was partitioned into three sub-libraries by splitting each class of sequences described above roughly into thirds. A small set of common sequences (125) was included in each sub-library.

DNA barcodes were designed using the dna-barcodes python package (https://github.com/feldman4/dna-barcodes). Barcodes were 8 nucleotides for the large sequence library and 5 nucleotides for the small sequence library. The barcodes were designed to have a minimum Levenshtein distance of 2 to all other barcodes, minimum GC content of 15%, maximum GC content of 85%, maximum homopolymer length of 3 nucleotides, and not to include “ATG”, “CGTCTC” (Esp3I 5' site), or “GAGACG” (Esp3I 3' site). DNA sequences for all protein sequences were designed using DNAchisel[41], enforcing a minimum GC content of 0.3 and maximum GC content of 0.7 within a window size of 60 nucleotides, with codon optimization for *Homo sapiens* (method = “use_best_codon”), avoiding rare codons with a threshold of 0.1, avoiding Esp3I cut sites, or sequences complementary to the ends of the primers used for library amplification.

Each DNA oligonucleotide sequence encoding one of the test proteins in the library was 300 nucleotides long. It included, from 5’ to 3’: a 5’ primer binding site, an Esp3I cut site, a DNA barcode (padded to a length of 12 nucleotides for all sequences), sequences for

binding the padlock oligonucleotide and an RT primer, the Kozak sequence, the DNA sequence encoding the test protein, an Esp3I cut site, and a 3' primer binding site (**Supplementary Figure 1B**). Libraries were cloned as described in the **Methods** into vectors containing an inducible promoter, fluorescent protein (GFP or SNAP-tag), and optionally, an oligomerization domain (**Supplementary Figure 1B**). Previously characterized *de novo* designed coiled coils were used for the valence 4 and 6 oligomerization domains ("HOTag7" and "HOTag3", respectively[42-45]), and FTH1 was used for the valence 24 oligomerization domain, which has previously been used to increase protein valence in condensate formation assays[1, 46].

Analysis of library composition

For each protein sequence in the large protein sequence library, all sequences in the human proteome, and all disordered regions within the human proteome, we computed the amino acid composition (a vector of length 20 containing the fraction of the sequence that is composed of each amino acid). We then computed the similarity between all of the amino acid composition vectors (cosine similarity matrix) and performed principal component analysis (for **Extended Data Fig. 2B**). **Extended Data Fig. 2B** shows projections onto the top two principal components. We repeated this analysis with dipeptide composition (for each protein sequence we computed a vector of length 400 containing the fraction of the sequence that is composed of each dipeptide). Disordered regions in the human proteome were identified from MobiDB[36], based on Mobid-lite predictions[47], downloaded with the following command:

```
curl -X 'GET'  'https://mobidb.org/api/download?format=tsv&proteome=UP000005640&prediction-disorder-mobidb_lite=exists&projection=acc,prediction-disorder-mobidb_lite,sequence'  -H 'accept: text/plain' > mobidb_disorder_preds_and_seqs.tsv
```

Hoechst staining control experiments

Individual test protein constructs were cloned into pGFP_24 and pSNAP_24 lentiviral vectors in an arrayed format, as described in the **Methods**. Lentivirus preparation and transduction was performed in an arrayed format in 96-well plates. Two days before imaging, protein expression was induced with 1 μg/mL doxycycline (final concentration). Cells expressing SNAP-tag fusion constructs were then stained with SNAP-tag as described in the **Methods**. For the Hoechst staining condition, cells were then stained with Hoechst as described in the **Methods**. Cells in both conditions were then imaged live.

Arrayed endogenous condensate colocalization experiments

Cells expressing test protein constructs were prepared in an arrayed format as described above for Hoechst staining control experiments. Because antibody staining requires fixation, which can cause artifacts[29], cells were imaged live, after Hoechst staining as described above, then immediately fixed, stained, and imaged again. This enabled comparison between phenotypes from live and fixed cells. Fixation and antibody staining was performed as follows. Cells were fixed with 4% paraformaldehyde in PBS for 30 minutes, then washed three times with PBS at room temperature. Cells were permeabilized with 0.1% Triton X-100 (Sigma-Aldrich Cat. # T8787-50ML) in PBS for 15 minutes at room temperature, then washed three times with PBS with 0.05% Tween. Cells

were then incubated in 3% BSA (Avantor Cat. # 0332-25G) in PBS at room temperature for 30 minutes, then stained with primary antibodies (antibody solution 1: mouse anti-PML 1:100 (Santa Cruz Biotechnology Cat. # sc-966), rabbit anti-SRRM2 1:1000 (Sigma-Aldrich Cat. # HPA041411-100UL); antibody solution 2: mouse anti-B23 (NPM) 1:600 (Sigma-Aldrich Cat. # B0556-100UL), rabbit anti-coilin 1:600 (Cell Signaling Technology Cat. # 14168S)) diluted in 1% BSA in PBS for 45 minutes. Cells were then washed with PBS with 0.05% Tween three times, then incubated with secondary antibodies (1:1000 donkey anti-mouse Alexa-Fluor 568 secondary antibody (Thermo Fisher Scientific Cat. # A10037) and 1:1000 donkey anti-rabbit Alexa-Fluro 647 secondary antibody (Thermo Fisher Scientific Cat. # A-31573)) diluted in 1% BSA in PBS for 45 minutes at room temperature. The antibody staining solution was then removed and replaced with imaging solution (200 ng/mL DAPI in 2X SSC). Cells were then imaged as described in the **Methods**, with additional images taken in the Alexa 568 (561 nm excitation, 570-639 nm emission filter) and Alexa 647 (640 nm excitation, 650-760 nm emission filter) channels.

GFP concentration calibration

To purify GFP, the DNA sequence encoding GFP, exactly matching the sequence used for all other experiments, was cloned into the pET-15b vector (Novagen). The plasmid, encoding the N-terminally His-tagged GFP, was transformed into *E. coli* BL21 Star (DE3) pLysS competent cells (Invitrogen). *E. coli* cells were cultured at 37°C until OD600 reached 1.8, when 0.5 mM isopropyl 1-thio-D-galactopyranoside (Gold Biotechnology) was added to induce protein expression. *E. coli* cells were then allowed to grow further at 16°C overnight. All subsequent steps were carried out at 4°C. *E. coli* cells were harvested by centrifugation at 6,100 x g for 10 min at 4°C (Beckman Coulter Avanti J-E, rotor JLA-8.1000). Cell paste was resuspended in 5 volumes of buffer A (50 mM Tris-HCl, pH 8.0, 400 mM NaCl, 5% [vol/vol] glycerol) containing cOmplete ULTRA Tablets (Millipore Sigma 6538282001) and disrupted using a LM20 microfluidizer (Microfluidics). Cell debris was removed by centrifugation at 16,800 x g for 1 hour at 4°C (Beckman Coulter Avanti J-E, rotor JLA-10.500). Supernatant was loaded onto a HisTrap FF crude column (Cytiva) pre-equilibrated with buffer A. The column was washed with 10 column volumes of buffer A containing 10 mM imidazole to remove nonspecifically bound *E. coli* proteins and then eluted with a linear gradient of 10 column volumes of buffer A containing 500 mM imidazole. Fractions containing GFP were pooled and exchanged to buffer B (50 mM Tris-HCl, pH 8.0, 3 M NaCl, 5% [vol/vol] glycerol). The protein was then loaded on a HiPrep 16/10 Phenyl HP (Cytiva) preequilibrated in buffer B. The column was washed with 10 column volumes of buffer B and then eluted with a linear gradient of 10 column volumes of buffer C (20 mM Tris-HCl, 5% [vol/vol] glycerol). Fractions containing GFP were pooled, concentrated, and then loaded on a Superdex 200 Increase 10/300 GL (Cytiva) pre-equilibrated in buffer A. The purified proteins were concentrated, aliquoted, frozen in liquid nitrogen, and stored at -80°C.

GFP concentration was quantified by preparing a dilution series in PBS, then measuring absorbance at 280nm (A280) on a nanodrop 2000 device and comparing to a standard of BSA (Thermo Fisher Scientific, Cat. #23208). Concentration was independently measured using the Pierce 660 chromogenic assay (Thermo Fisher Scientific, Cat. #22662) by combining 90μl of assay solution with 10μl of sample before measuring the

absorbance at 660nm on a Nanodrop 200 device. Final protein concentration was calculated as the average of the concentration values obtained with both methods. After quantification, GFP concentration standards were prepared in imaging media and transferred to a 96-well glass bottom plate for imaging (CellVis, Cat. # P96-1.5H-N). 18 images were acquired per well with an Opera Phenix High-Content Screening System, with the same settings as used for all phenotype imaging (described above). Mean intensity was calculated for each image and for each GFP concentration. Data were fit to a linear model (concentration = intensity $\times$ slope), which was then used to compute concentrations from phenotype images. We could robustly measure GFP concentrations as low as 0.06 μM.

Western blot for FTH1 oligomerization domain

The western blot was performed as follows. 500,000 cells were seeded in a 75 cm^2 dish. The next day, media was exchanged and supplemented with doxycycline (final concentration 1 μg/mL). Cells were incubated for 48 hours before they were harvested and used for protein extraction. Nuclei were extracted using a Nuclei EZ Prep kit (Sigma-Aldrich, Cat. #NUC101-1KT) according to the manufacturer's instructions. Nuclei were then lysed in M-PER (Thermo Fisher Scientific, Cat. #78501; supplemented with Protease Inhibitors and EDTA; Thermo Fisher Scientific, Cat. #87786) for 30 minutes on ice. Lysates were vortexted and the insoluble fraction was removed by centrifugation at full speed for 30 minutes at 4°C in a tabletop centrifuge. Finally, protein content was measured based on sample absorbance at 280 nm on a Nanodrop 2000 device, and 20μg of protein were boiled for 5 minutes at 95°C with 4XBOLT (Life Technologies, Cat. #B0007) supplemented with 10X Reducing Reagent (Thermo Fisher Scientific, Cat. #B0009). As a positive control, we also loaded 10 ng or 100 ng of purified FTH1 (ProSpec, Cat. #PRO-658). Samples were separated on 4-12% Bis-Tris gradient gels (Thermo Fisher Scientific, Cat. #NW04125BOX) and transferred onto a PVDF membrane using an iBlot2 device according to the manufacturer's instructions (20V, 7 minutes). Next, the membrane was blocked using Blocking Buffer (LiCOr, Cat. #927-60001) for at least 30 minutes, before probing the membrane with primary antibody (mouse anti-FTH1 1:1000 (Sigma Aldrich, Cat. # MABC602) and rabbit anti-PCNA 1:1000 (Thermo Fisher Scientific, Cat. # SY12-07)) overnight at 4°C. The next day, the membrane was washed with TBS-T (Thermo Fisher Scientific, Cat. #28360) before incubating it with secondary antibody (anti-mouse IRDye-680RD, 1:10.000; LiCOr, Cat. #926-68072) for 45 minutes. Finally, the blot was washed 3 times for 5 minutes in TBS-T before imaging on a ChemiDoc imager. Relative quantification of protein content was performed in Fiji (https://imagej.net/software/fiji/downloads). A region devoid of signal was used to measure the background, which was subtracted from all measurements before comparing the relative amounts of endogenous FTH1 and the library constructs. All measurements were made relative to the PCNA loading control.

Image analysis: detecting condensates in phenotype images

A flat field correction was applied to each image by dividing the intensity value at each pixel by the mean value at that pixel over all images within the well, smoothed with a Gaussian filter with a width 1/10 of the image dimension. Nuclei were segmented in the Hoechst channel with Cellpose[48] using the "nuclei" model, with a diameter of 87. Nuclei

that touched the edge of the image were discarded. For each nucleus, the total nucleus area, mean Hoechst intensity, mean GFP/SNAP-tag intensity, standard deviation of GFP intensity, and the correlation between the Hoechst and GFP/SNAP-tag signal were calculated. To identify condensates, for each nucleus, regions with little to no signal ("holes") were first identified in the GFP/SNAP-tag channel (often, there was little signal in the nucleolus). If holes comprised >75% of the total pixels in the nucleus, then the holes were discarded. The threshold intensity for identifying condensates was computed as the mean of the pixel intensity values, excluding holes, plus 3x the standard deviation of the pixel intensity values, excluding holes. If this value was below a cutoff intensity (300 a.u.), then the threshold intensity was set to the cutoff intensity. Pixels above this threshold intensity were assigned as condensate pixels. Next, holes in the condensates were filled, and then any isolated regions of less than four pixels were removed. Individual condensates were assigned using watershed-based segmentation. Condensates that touched the edge of the cell were discarded.

To compute the average dilute phase intensity, condensates were first expanded by 8 pixels, then the intensities of the remaining nuclear pixels minus holes were averaged. The mean and standard deviation of pixel intensities were computed over all condensates. The total, mean and standard deviation of the condensate area, eccentricity, and intensity were computed, as well as the total number of condensates. The fraction of the signal in the condensates was computed as the total intensity in the condensates divided by the total intensity in the nucleus. The fraction of cells with condensates was calculated as the fraction of all cells expressing a particular protein sequence that contained at least one condensate, identified as described above.

Image analysis: *in situ* SBS
In situ SBS data were analyzed using a slightly modified version of the previously published software[49] (https://github.com/kkappel1/OpticalPooledScreens2023). Nuclei and cells were segmented using Cellpose in the DAPI and MiSeq-C channels, respectively, with a diameter of 21. The value of THRESHOLD_READS was set to 200 and MAXED_WIDTH was set to 3. Cells with reads corresponding to more than one barcode were discarded. Reads that did not exactly map to a barcode in our libraries were discarded.

Image analysis: mapping phenotype to SBS images
To map phenotype images to the SBS images, a small subset of the phenotype images (typically 30) was first taken and rescaled, such that each pixel corresponded to the same distance as in the SBS images. Next, a brute force cross correlation scan was performed, calculating Pearson's correlation coefficient between the phenotype and SBS images, when scanning the position of the phenotypic image across all of positions of the SBS image. The position with the best correlation for each image was noted. Images with correlation above 0.35 were used to fit a linear model using RANSAC regression to relate xy coordinates of the phenotype images to coordinates of the SBS images. Using this model, coordinates in the SBS image space were obtained for all of the phenotype images, and a set of SBS image tiles was identified for mapping each phenotype image to. Next, each phenotype image was scanned across the SBS images and the correlation

was calculated at each position. The image and position with the best correlation for each was noted. If the maximum correlation between the phenotype and SBS image was less than 0.3, it was omitted from further analysis.

To match the cells in a phenotype image to the cells in an SBS image, nuclei centroid positions were used, then matches were filtered based on mean GFP/SNAP-tag intensity and gray level cooccurrence matrix (GLCM) dissimilarity in the GFP/SNAP-tag channels. Nuclei were matched if their centroids were within 15.0 µm and if the ratio between the distance to nearest nucleus and the distance to the next closest nucleus was less than 0.9. For each well, a linear model was fit using RANSAC regression between the GFP/SNAP-tag intensity and GLCM dissimilarity values for the SBS versus phenotype images. Outliers, defined as noted below, were discarded:

Outliers based on GLCM dissimilarity: | GLCM_pred – GLCM_ph | > (0.08*GLCM_SBS + 1)
Outliers based on intensity: | int_pred – int_ph | > (0.08*int_SBS)

where GLCM_pred is the GLCM dissimilarity value predicted for the SBS image from the GLCM dissimilarity value for the phenotype image (GLCM_ph) using the linear model. int_pred is the intensity value predicted for the SBS image from the intensity value for the phenotype image (int_ph) using the linear model. GLCM_SBS and int_SBS are the GLCM dissimilarity and intensity values for the SBS image, respectively.

Image analysis: SNAP-tag intensity normalization
To normalize for differences in SNAP-tag intensity between independent experiments performed at different times, cells expressing the same proteins in each experiment were used to compute a correction factor. Mean intensity over all cells expressing the same protein in each experiment was computed, and linear regression was used to compute a slope and offset value for the mean intensity values per protein between the different experiments. There were more than 3000 corresponding proteins in the experiments that were normalized against each other. The calculated slope and offset were used to normalize the intensity values for each individual cell. After applying this normalization, histograms of intensity values for all SNAP-tag experiments were inspected to confirm that they matched closely.

Image analysis: live-cell time-lapse experiments
Phenotype image analysis was performed for each frame as described above. Cells were tracked between subsequent frames, by matching nuclei with the smallest distance between centroids. Any nucleus that was matched to multiple nuclei in the previous or subsequent frames was omitted. Only nuclei that were tracked in every frame were retained for further analysis. The final frame was then matched to SBS images, as described above.

To compute threshold concentrations, for each tracked cell, the first frame in which there was at least one condensate was identified and total protein concentration was computed from the mean nuclear intensity, using the GFP concentration calibration described

above. If a cell did not form condensates in any of the frames, it was omitted from this calculation. If more than 30% of cells expressing a given test protein did not form condensates in any of the frames, a threshold concentration was not computed for that test protein. For each test protein, the median value of these concentrations was taken as the threshold concentration.

Classifying nucleolus- and chromatin-localizing sequences

Two separate classifiers (one for GFP and one for SNAP-tag fused sequences) were trained that take an image as input and predict whether the fluorescently tagged protein sequence in the image localizes to the nucleolus or chromatin, forms other condensates, no condensates, or is expressed poorly (for GFP sequences only). From the input image, a set of pre-defined features were computed: the fraction of the total nuclear area occupied by condensates, the mean area per condensate, the standard deviation of the condensate area, the mean condensate eccentricity, the standard deviation of the condensate eccentricity, the GLCM dissimilarity, and the number of condensates. The image was then preprocessed to yield a resized, nuclear masked image (100 x 100 pixels) and a nuclear distance matrix (pixels were assigned values equal to the minimum distance to the nuclear boundary, rescaled to be between 0 and 1, with positive values for pixels inside the nucleus (0 to 1) and negative values for pixels outside the nucleus (-1 to 0)), which was then provided as input to the pretrained cytoself model[50], from which the local latent representation was extracted and converted to a feature spectrum (vector of length 2,048), and separately subjected to UMAP dimensionality reduction to yield 3 components[51]. The global latent representation was additionally extracted and subjected to UMAP dimensionality reduction to yield 3 components. An SVM classifier with a linear kernel (implemented in python scikit-learn[52]) was then trained using a manually labeled set of images from the large pooled experiments containing, for the GFP sequences, 3,600 images of protein sequences that form condensates for 360 distinct barcodes (10 images per barcode) and 310 images of protein sequences that do not form condensates for 31 barcodes (10 images per barcode). For the SNAP-tag sequences, the manually labeled set of images contained 10,780 images of protein sequences that form condensates for 1,078 distinct barcodes (10 images per barcode) and 310 images of protein sequences that do not form condensates for 31 distinct barcodes (10 images per barcode). For the GFP sequences, the manually labeled set of images included 499 labeled images of nucleolus-localizing sequences and 151 labeled images of chromatin-localizing sequences; for the SNAP-tag sequences, there were 957 labeled images of nucleolus-localizing sequences and 506 labeled images of chromatin-localizing sequences. The accuracy of the classifier was assessed by 5-fold cross validation, keeping images from sequences with the same barcode together in either the train or test group. The final models were trained using all the labeled images.

For each protein sequence in our libraries, we had images of many cells (median = 220 cells). Nucleolar and chromatin sequences were defined as sequences for which at least 30% of these cells were classified as nucleolar or chromatin localizing, respectively, by our classifier. Sequences for which fewer than 30% of the cells contained condensates were classified as not forming condensates (“none”). For analyses in **Fig. 4** and **Fig. 5**, nucleolar and chromatin localizing sequences were defined subject to the additional

criteria that fewer than 30% of cells were classified as chromatin or nucleolar localizing, respectively (sequences with >30% nucleolar cells *and* >30% chromatin cells were omitted). Throughout our analyses, when comparing sequences that formed condensates versus those that did not, sequences with $f_{condensates}$ < 0.3 were defined as not forming condensates, while sequences with $f_{condensates} \geq 0.3$ were defined as forming condensates.

Evaluating sequence patterning hypotheses

To assess the impact of multiple patterning parameters (δ_{+-} , Ω_{STNQCH} , Ω_{FWY} , Ω_{ILMV} , Ω_{-}, Ω_{+}) on condensate formation, a set of "unpatterned" sequences was first identified for each patterning parameter. For each set of scrambled sequences with the same amino acid composition, excluding wild type (natural) base sequences (in case these sequences contain a specific sequence motif that could be disrupted by scrambling), the sequence with the lowest absolute value of the patterning parameter of interest were defined as "unpatterned" sequences. Unpatterned sequences with an absolute patterning value greater than 0.5 were omitted from further analysis. Alternatively, unpatterned sequences containing less than 10% of patterned group of amino acids, or for δ_{+-}, 10% positively charged residues and 10% negatively charged residues, were also omitted from further analysis. To specifically assess whether changing amino acid patterning can increase condensate formation, any unpatterned sequences for which the fraction of cells with condensates was greater than 0.3 were filtered out. Next, all scrambled variants of the unpatterned sequence in which other patterning parameters, besides the specific one of interest, had changes relative to the unpatterned sequences less than -2.0 or greater than 2.0 were filtered out. For δ_{+-} patterning changes in Ω_{-} or Ω_{+} were not checked. For Ω_{-} and Ω_{+} patterning changes in δ_{+-} were not checked. Of the remaining sequences, those with changes in the patterning parameter of interest of greater than 2.0 relative to the unpatterned sequence were assigned to be "increased" patterning sequences and those with changes of less than -2.0 were assigned to be "decreased" patterning sequences. The change in the propensity to form condensates was then computed for each increased or decreased patterning sequence relative to the unpatterned sequence as $f_{condensates}$ for the patterned sequence minus $f_{condensates}$ for the unpatterned sequence. For each group of sequences (e.g., increase $\Omega_{FWY,}$ decrease $\Omega_{FWY,}$ increase $\Omega_{ILMV,}$ decrease $\Omega_{ILMV,}$ etc.) significant deviation in $f_{condenastes}$ from 0 was tested by a two-sided Wilcoxon signed rank test.

Smoothed empirical CDF test for wild type base sequences versus scrambled variants

To compute the probability of observing $f_{condensates}$ for a wild type base sequence given the distribution of $f_{condensates}$ values for all of the scrambled variants of that base sequence, we performed a smoothed empirical CDF test. Each scrambled variant $f_{condensates}$ value was treated as a Gaussian distribution centered at its observed value, with a standard deviation equal to 0.1, to account for noise in the experimental measurements. For a given wild type base sequence $f_{condensates}$ value, the cumulative probability was calculated by integrating the contributions of all scrambled data points up to the wild type value using the cumulative distribution function of the Gaussian. The overall probability of the wild type $f_{condensates}$ point being greater than the scrambled data points was then computed as 1 – CDF. The overall probability of the wild type $f_{condensates}$ point being less than the

scrambled data points was equal to the CDF. The probability of the wild type value coming from the distribution of scrambled values was computed as the minimum of 1 – CDF and CDF.

Consistency score calculation

For **Extended Data Fig. 5A** (heatmap), consistency scores were computed as follows. For each mutation type, $\Delta f_{condensates}$ (mutant – base sequence) was computed. Mutations that promoted, disrupted, or caused no change to condensate formation were defined as those with $\Delta f_{condensates}$ values ≥ 0.2, ≤ -0.2, or between -0.2 and 0.2, respectively. For each mutation type, the most common effect was found (promoted, disrupted, or caused no change to condensate formation for most base sequences), and the consistency score was defined as the fraction of the base sequences for which the mutation type had that effect. The scores range from 0 to 1, with 1 indicating that the mutant has the majority effect for 100% of the base sequences. Consistency scores were separately computed for base sequences with $f_{condensates} < 0.5$ and $f_{condensates} \geq 0.5$.

For **Supplementary Table 4**, this calculation was modified slightly to compute a single consistency score for each mutation type, over all base sequences. First, for each base sequence, each mutation type was assigned as either promoting, disrupting, or causing no change to condensate formation, as described above. For base sequences with $f_{condensates} < 0.5$ ("base<0.5") and separately for base sequences with $f_{condensates} \geq 0.5$ ("base>0.5"), the fraction of mutants that promoted, disrupted, or caused no change to condensate formation was calculated. The overall effect of a mutation type was defined as promoting condensate formation if the fraction of "base<0.5" mutants that promoted condensates was > 0.5 and if the fraction of "base>0.5" mutants that promoted condensates plus those that caused no change was > 0.5. We defined the overall effect of a mutation type as disrupting condensate formation if the fraction of "base>0.5" mutants that disrupted condensates was > 0.5 and if the fraction of "base<0.5" mutants that disrupted condensates plus those that caused no change was > 0.5. Other mutations were defined as having no effect on condensate formation. If the overall effect of a mutation type was to promote condensate formation, the consistency score was defined as the fraction of mutants for all base sequences that promoted condensates plus the "base>0.5" mutants that caused no change. If the overall effect of a mutation was to disrupt condensate formation, the consistency score was defined as the fraction of mutants for all base sequences that disrupted condensate formation plus the "base<0.5" mutants that caused no change. If the overall effect of a mutation was to cause no change to condensate formation, the consistency score was defined as the fraction of mutants (for all base sequences) that caused no change to condensate formation. Mutation types were included in **Supplementary Table 4** if there was data for at least 6 base sequences in the GFP fusion medium concentration bin.

Analyzing possible cation-π interactions with FINCHES

FINCHES[5] was used to predict a homotypic interaction map for each test protein sequence in the large sequence library, with the Mpipi force field[53] with a sliding window size of 11 residues. Next, cation-π interactions were defined as those between a window with a cation at the center (arginine or lysine) and a window with an aromatic residue at

the center (phenylalanine, tyrosine, or tryptophan). For each test protein sequence, the total number of possible attractive cation-π interactions was counted (interaction parameter $\varepsilon < 0$).

Longer protein sequence experiments

The long sequence library was constructed, containing 69 protein sequences ranging in length from 85 to 296 amino acids. 36 of the protein sequences are fragments of natural protein sequences, selected in the same manner in which the natural protein sequences in the small sequence library were selected. The remaining sequences are designed variants of these sequences in which several sequence features, especially charged and aromatic residues, were mutated. Experiments were performed as described in the **Methods**, with the following exceptions. DNA sequences were synthesized as eBlocks (Integrated DNA Technologies) and were cloned individually in an arrayed format. DNA sequences were mixed prior to lentivirus production. For these preliminary, proof-of-concept experiments, phenotype imaging was performed after fixation, followed by eight rounds of *in situ* SBS to read out the barcodes. All protein sequences, DNA sequences, and barcodes are listed in **Supplementary Data 2**.

Supplementary Figures

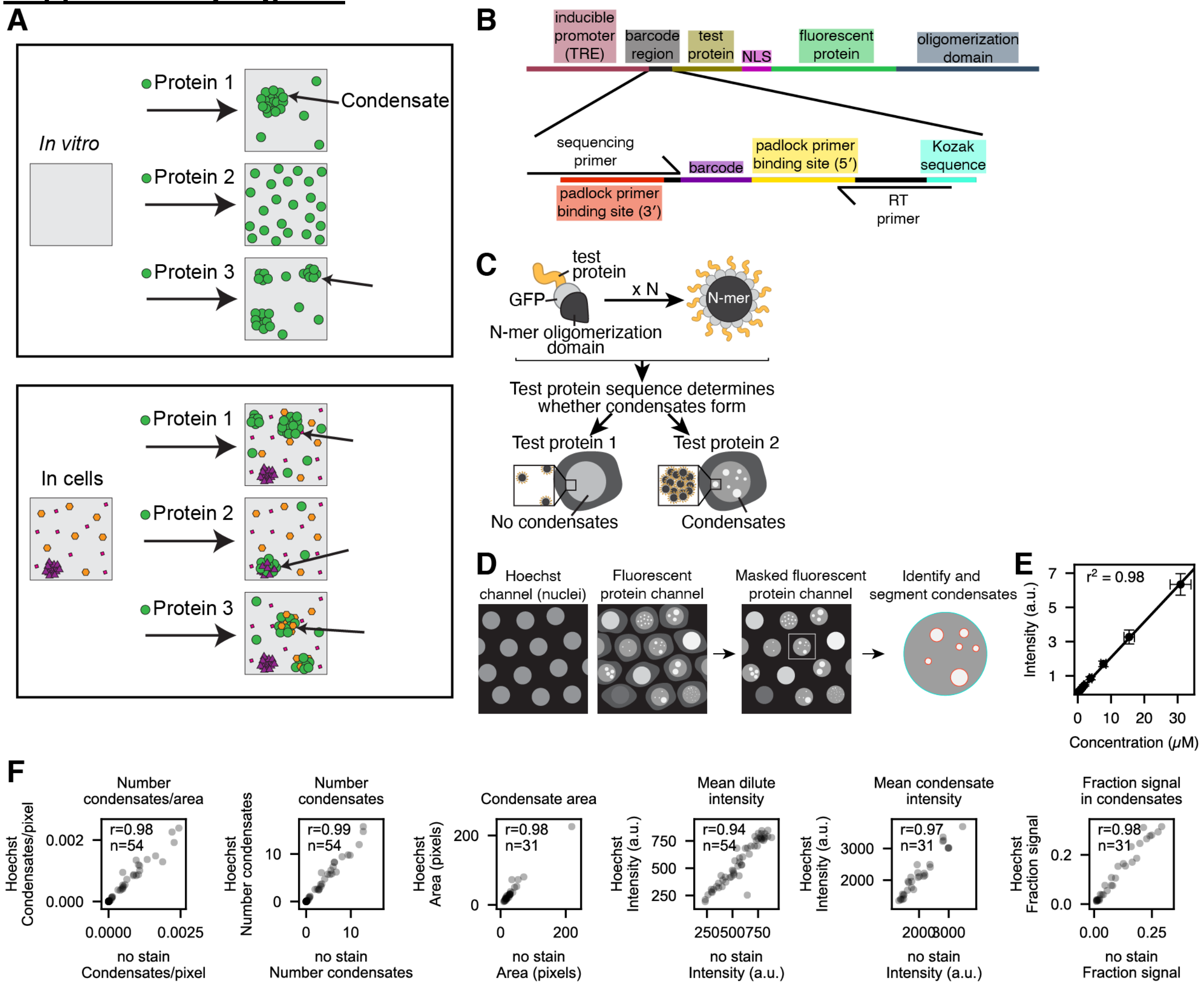

Supplementary Figure 1. Additional details about CondenSeq.

(A) Schematic highlighting the difference between assessing the propensity of a protein sequence to form condensates *in vitro* (top) vs in cells (bottom). The boxes on the left show the *in vitro* (top) or cellular environment (bottom) before adding a protein of interest (green circles). The boxes on the right show the *in vitro* or cellular environment after the addition of the protein of interest. The cellular environment contains many endogenous factors (proteins, nucleic acids, etc.; denoted with orange hexagons, purple triangles, and pink squares), which can affect the propensity of a protein to form condensates. The arrows in the boxes on the right point to examples of condensates.

(B) Expression vector schematic (result of protein sequence library cloned into pGFP_1, pGFP_4, pGFP_6, pGFP_24, pSNAP_1, pSNAP_4, pSNAP_6, or pSNAP_24). NLS = nuclear localization signal.

(C) Test proteins are fused to GFP or SNAP-tag and an oligomerization domain to manipulate valence. The test protein sequence determines whether or not condensates form.

(D) Schematic overview of condensate detection. Nuclei are segmented in the Hoechst channel, then condensates are detected and segmented (right, red outlines) within the nuclei (right, blue outline) in the fluorescent protein channel (**Supplementary Note 1**).

(E) GFP intensity versus concentration calibration curve. Error bars for intensity represent the standard deviation in intensity over multiple images. Error bars for the concentration represent the standard deviation of the quantification of the GFP standard concentration. (F) Comparison between phenotypes for cells that were stained with Hoechst or not. Each dot represents the mean value over all cells expressing a specific test protein. Pearson correlation coefficients are noted on the plots.

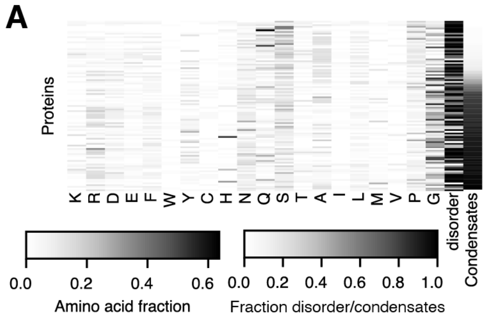

Supplementary Figure 2. Small sequence library composition.
(A) Single letters indicate specific amino acids. Grayscale indicates the fraction of the total sequence composed of a particular amino acid, or the fraction of the sequence that is predicted to be disordered or the fraction of cells expressing the sequence that contain condensates within the medium concentration bin. The mean predicted disorder for the library is 80%.

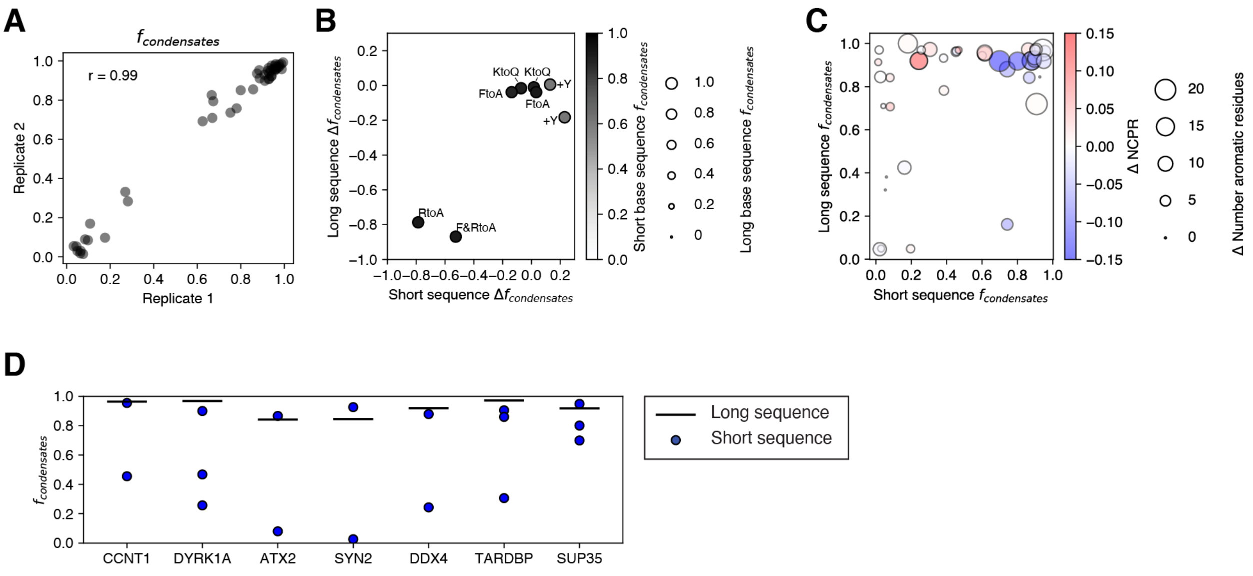

Supplementary Figure 3. Proof-of-concept tests applying CondenSeq to longer protein sequences.

(A) Reproducibility between replicates for the long sequence library. Each point represents $f_{condensates}$ for one protein sequence within the medium concentration bin. Data is shown for GFP fusions.

(B) Comparison of $\Delta f_{condensates}$ for analogous short versus long designed sequence variants (i.e., the short and long base sequences are fragments of the same full-length protein and the designed variants of each modify the same sequence features). Each point represents the difference in $f_{condensates}$ for a designed sequence relative to its base sequence. The points are labeled with descriptions of the mutation types. For example, “RtoA” means that arginine residues are mutated to alanine residues. The points are colored (gray scale) by the value of $f_{condensates}$ for the short base sequence. The size of the points represents the value of $f_{condensates}$ for the long base sequence. Data is shown for GFP fusions.

(C) Comparison of $f_{condensates}$ for short sequences versus extended versions from the long sequence library. Each short sequence is a subset of the corresponding long sequence. Each point represents one pair of short and long sequences. The points are colored by the change in the NCPR for the long sequence relative to the short sequence ($NCPR_{long}$ – $NCPR_{short}$). The size of the points represents the change in the number of aromatic residues (F, Y, and W) for the long sequence relative to the short sequence (Number $aromatic_{long}$ – Number $aromatic_{short}$). Data is shown for GFP fusions.

(D) Comparison of $f_{condensates}$ for short sequence subsets of longer protein sequences. Black lines represent $f_{condensates}$ values for long protein sequence fragments from the proteins listed on the x-axis. The blue points represent $f_{condensates}$ values for short protein sequence subsets (66 amino acids) of the long protein sequences. All protein sequences are listed in **Supplementary Data 2**. Data is shown for GFP fusions.

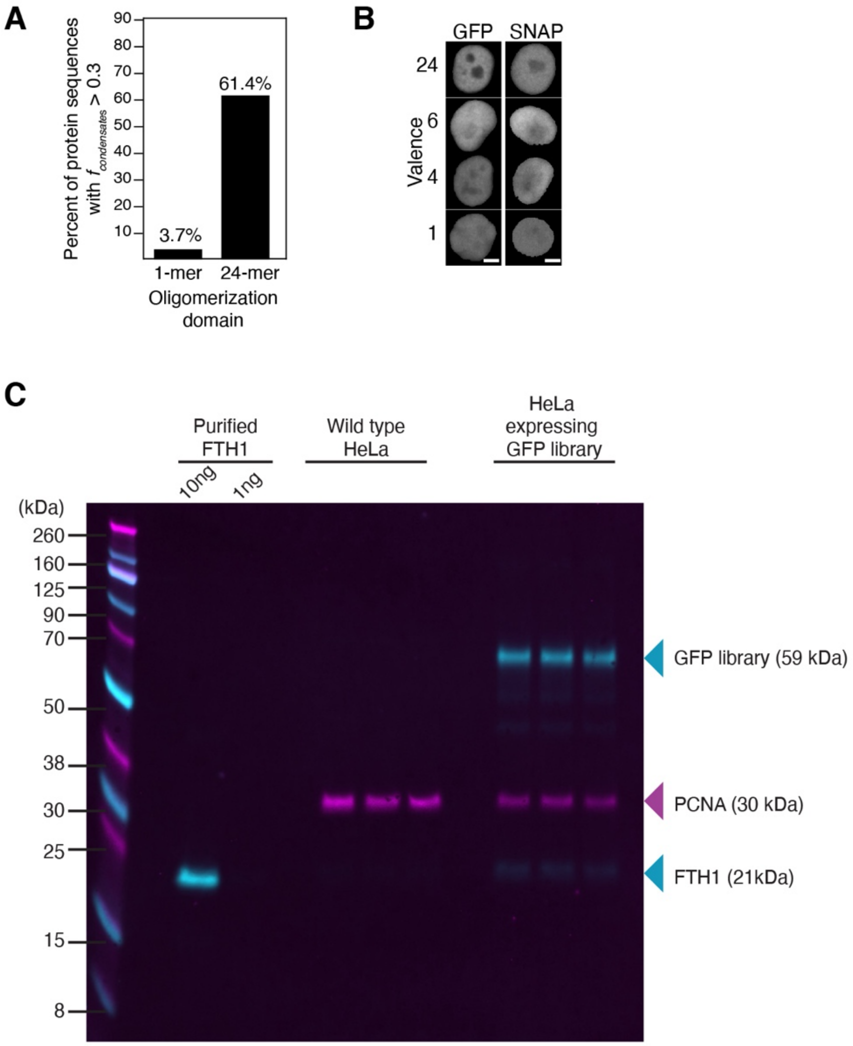

Supplementary Figure 4. Details of the small sequence library.

(A) The percent of protein sequences in the small sequence library that formed condensates in more than 30% of cells when fused to the FTH1 24-mer oligomerization domain (valence = 24) or to no oligomerization domain (1-mer, valence = 1). All sequences are also fused to GFP. Data is shown for the medium concentration bin.

(B) Representative images of nuclei (masked) expressing GFP and SNAP-tag without fused test proteins for each oligomerization domain. Scale bar = 5 μm. Two independent biological replicates of this experiment were performed with the same results.

(C) Western blot for FTH1, the 24-mer oligomerization domain (24-mer), in the nuclei of HeLa cells transduced with nothing (“wild type HeLa”) or with the GFP library fused to the 24-mer oligomerization domain (“HeLa expressing GFP library”). The three lanes for wild type HeLa and HeLa expressing GFP library represent independent biological replicates. Purified FTH1 was loaded in the left-most lanes as a positive control. To quantify the relative expression level of the GFP library versus endogenous FTH1, in the HeLa expressing GFP library samples, we compared the bands for the GFP library fused to FTH1 (59 kDa) to the bands for FTH1 only (21 kDa), normalizing for the amount of sample loaded using the PCNA bands. Libraries are expressed at ~12-16 times higher levels than endogenous FTH1.

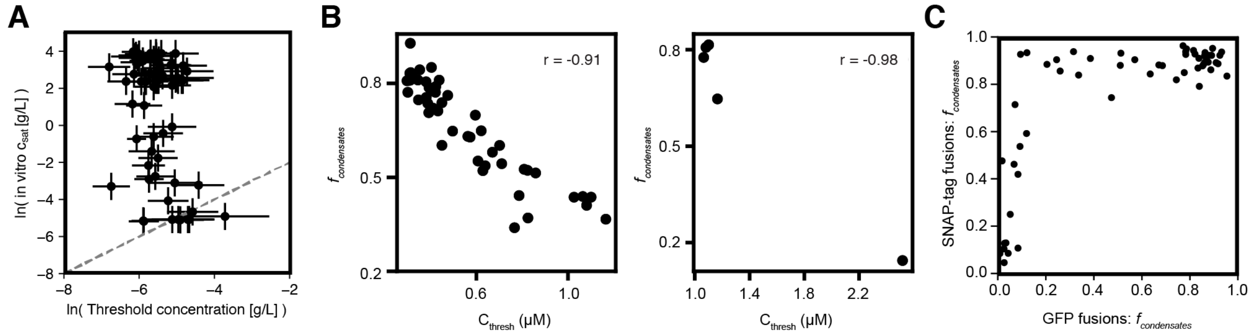

Supplementary Figure 5. Additional details and controls for CondenSeq measurements. (A) Threshold concentrations for test protein sequences in the small sequence library measured with CondenSeq via live-cell timelapse imaging compared to *in vitro* C_{sat} values obtained from a recently developed predictor[54]. Each point represents a single test protein sequence. The dashed gray line marks y=x. Error bars for the *in vitro* C_{sat} values represent the previously reported prediction uncertainty[54]. Error bars for C_{thresh} represent standard deviations over all cells expressing the given protein sequence.

(B) Agreement between threshold concentrations and the fraction of cells with condensates ($f_{condensates}$) in the low (left) and medium (right) concentration bins. Each dot represents a single protein sequence. Pearson's correlations (r) are noted on the plots.

(C) Comparison between the fraction of cells with condensates for test proteins fused to GFP versus SNAP-tag in the medium intensity bin. Each point represents a single test protein sequence. Test proteins tend to form condensates more readily when fused to SNAP-tag compared to GFP, but sequence feature trends are consistent for both fluorescent fusion proteins (**Supplementary Figure 7F**).

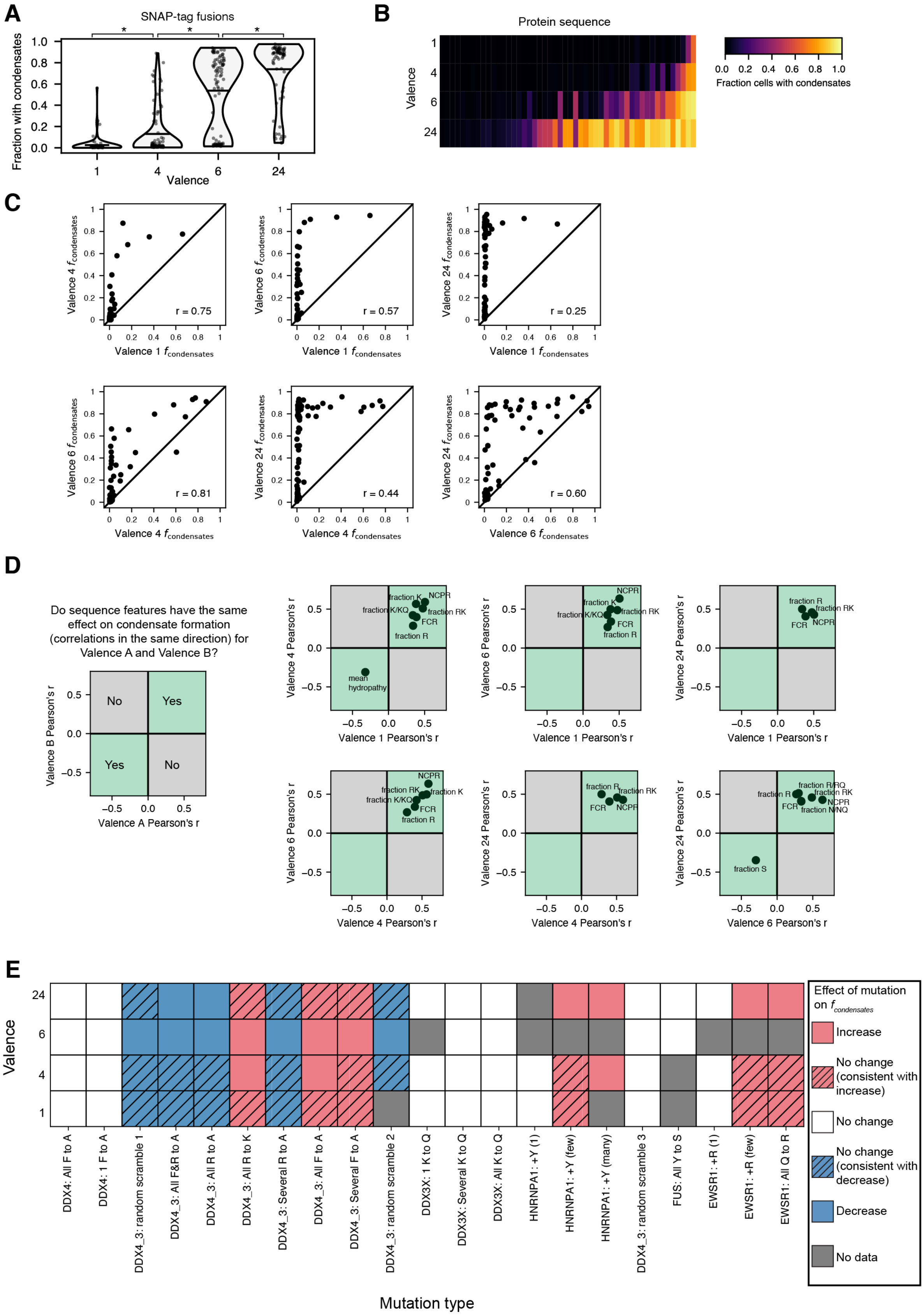
A
SNAP-tag fusions
Fraction with condensates
Valence
B
Protein sequence
Valence
Fraction cells with condensates
C
Valence 4 $f_{condensates}$
Valence 1 $f_{condensates}$
r = 0.75
Valence 6 $f_{condensates}$
r = 0.57
Valence 24 $f_{condensates}$
r = 0.25
Valence 4 $f_{condensates}$
r = 0.81
r = 0.44
Valence 6 $f_{condensates}$
r = 0.60
D
Do sequence features have the same effect on condensate formation (correlations in the same direction) for Valence A and Valence B?
Valence B Pearson's r
Valence A Pearson's r
No
Yes
Yes
No
Valence 4 Pearson's r
Valence 1 Pearson's r
fraction K
NCPR
fraction K/KQ
fraction RK
FCR
fraction R
mean hydropathy
Valence 6 Pearson's r
Valence 24 Pearson's r
Valence 4 Pearson's r
Valence 6 Pearson's r
fraction R/RQ
fraction N/NQ
fraction S
E
Valence
Mutation type
DDX4: All F to A
DDX4: 1 F to A
DDX4_3: random scramble 1
DDX4_3: All F&R to A
DDX4_3: All R to A
DDX4_3: All R to K
DDX4_3: Several R to A
DDX4_3: All F to A
DDX4_3: Several F to A
DDX4_3: random scramble 2
DDX3X: 1 K to Q
DDX3X: Several K to Q
DDX3X: All K to Q
HNRNPA1: +Y (1)
HNRNPA1: +Y (few)
HNRNPA1: +Y (many)
DDX4_3: random scramble 3
FUS: All Y to S
EWSR1: +R (1)
EWSR1: +R (few)
EWSR1: All Q to R
Effect of mutation on $f_{condensates}$
Increase
No change (consistent with increase)
No change
No change (consistent with decrease)
Decrease
No data

Supplementary Figure 6. Assessing the impact of valence on condensate formation.
(A) Fraction of cells that contain condensates for the SNAP-tag small sequence library fused to the four different oligomerization domains. Each point represents one protein sequence. Black lines show the means. The increases in $f_{condensates}$ as valence is increased are all statistically significant (valence = 1 vs 4: $p = 3\times10^{-5}$; 4 vs 6: $p = 6\times10^{-18}$; 6 vs 24: $p = 4\times10^{-9}$; two-sided paired t-test, p-values adjusted for multiple comparisons with Bonferroni correction, medium test protein concentration bin).
(B) The fraction of cells with condensates ($f_{condensates}$) for test proteins with different valences (fused to different oligomerization domains, with valence 1, 4, 6, or 24). Each row represents a single protein sequence.
(C) $f_{condensates}$ for test proteins fused to GFP and to different oligomerization domains, with valence 1, 4, 6, or 24. Each point represents a single test protein sequence. Points are only shown for test protein sequences with expression within the medium concentration bin. Pearson's r values are noted on the plots. All test protein sequences and $f_{condensates}$ values are provided in **Supplementary Data 1**.
(D) Pearson's r values for sequence features of small library sequences (medium concentration bin) versus $f_{condensates}$ for valence 1, 4, 6, and 24. Each dot represents one sequence feature (the features are labeled in the plots). In each plot, dots are only shown for sequence features with statistically significant Pearson's r values for both valences ($p < 0.05$). Note that due to the small library size (99 test protein sequences), our statistical power to detect significant correlations is limited. Positive r values mean that the values of the sequence features are higher for sequences with higher $f_{condensates}$. All sequence features with statistically significant correlations with $f_{condensates}$ are correlated in the same direction (the r values have the same sign) between different valences (for example, the Pearson's r value for NCPR is positive for valence 1 and also positive for valence 24, meaning that higher NCPR promotes condensate formation for both valence 1 and valence 24).
(E) The impacts of mutations on condensate formation for test protein sequences fused to GFP and to different oligomerization domains, with valence 1, 4, 6, or 24. Each box represents the change in $f_{condensates}$ for a single mutant test protein sequence relative to its base sequence. Each row contains test protein sequences fused to a particular oligomerization domain. Each column contains a different mutant test protein sequence. Boxes are colored gray if there is no data for the mutant or base sequence within the medium concentration bin. Boxes are colored blue if the mutant $f_{condensates}$ value is less than the base sequence $f_{condensates}$ value (mutant $f_{condensates}$ - base sequence $f_{condensates} < -0.2$). Boxes are colored red if the mutant $f_{condensates}$ value is greater than the base sequence $f_{condensates}$ value (mutant $f_{condensates}$ - base sequence $f_{condensates} > 0.2$). Note that when $f_{condensates}$ values are near 0 or 1, they are outside the dynamic range of our measurements and it may not be possible to observe changes in propensity to form condensates. For example, if a base sequence has $f_{condensates}$ of 1, then it is not possible to observe an increase in $f_{condensates}$, because $f_{condensates}$ values are bounded between 0 and 1. Boxes contain diagonal hashes if the base sequence $f_{condensates}$ value is outside the dynamic range (base sequence $f_{condensates} < 0.2$ or > 0.8) and if there is no change in the mutant $f_{condensates}$ value relative to the base sequence $f_{condensates}$ value ($|$mutant $f_{condensates}$ - base sequence $f_{condensates}| < 0.2$). Boxes with diagonal hashes are colored according to the most common impact for that particular mutant test protein across the different

valences (red if the mutation decreases $f_{condensates}$, blue if the mutation increases $f_{condensates}$). Simple descriptions of the mutations are shown below the x-axis, for example "DDX4: All F to A" means that all phenylalanine residues are mutated to alanine residues within the DDX4 base sequence; "EWSR1: All Q to R" means that all glutamine residues are mutated to arginine within the EWSR1 base sequence. All test protein sequences and $f_{condensates}$ values are provided in **Supplementary Data 1**.

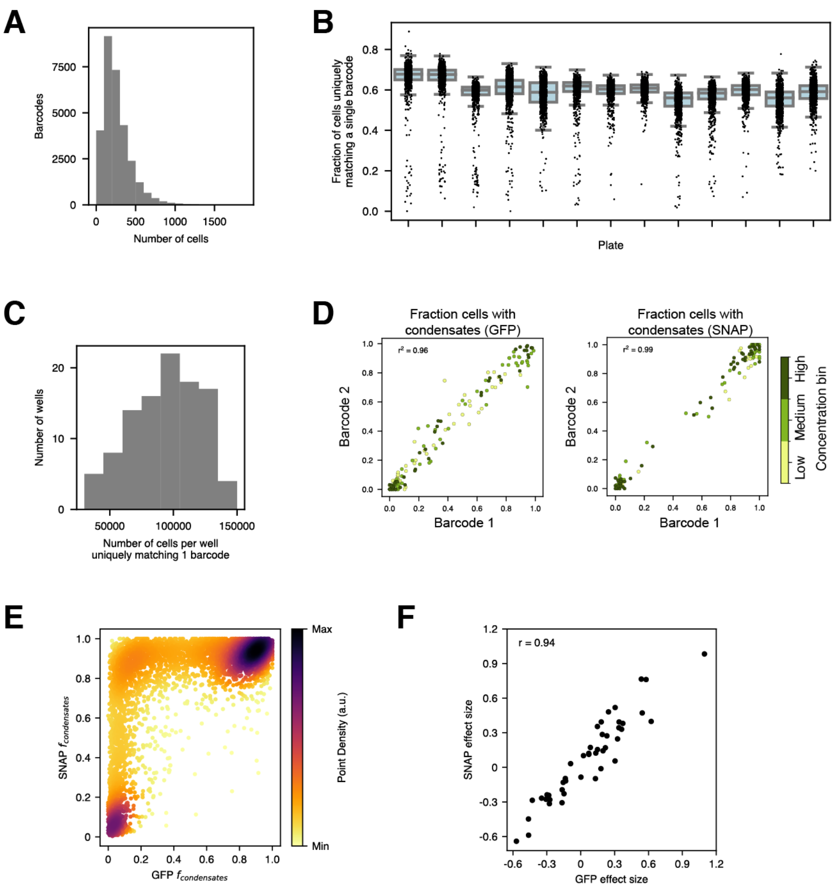

Supplementary Figure 7. Large sequence library quality control.

(A) The number of cells per barcode in the large sequence library (GFP and SNAP-tag fusions) (median = 220 cells/barcode).

(B) Fraction of cells within each image (10x magnification) with a detected barcode that exactly matches a single barcode within the designed library. Each point represents a single image. Each box represents one plate.

(C) The number of cells per well (of a 24-well plate) uniquely matching a single barcode within the designed library (median = 101,408 cells/well).

(D) Assessing the effects of the barcode sequence on condensate formation for test protein sequences fused to GFP (left) and SNAP-tag (right). Each point represents a single test protein sequence. The fraction of cells with condensates was measured for each protein sequence with two distinct barcodes, independently (identical protein sequences with different barcode sequences, measured as part of the large sequence library, **Supplementary Note 1**). Colors indicate the concentration bin for which the measurement of $f_{condensates}$ was made.

(E) $f_{condensates}$ for protein sequences in the large sequence library fused to GFP versus SNAP-tag. Each point represents one test protein sequence. Points are colored by the local density of points in the plot, calculated using a Gaussian kernel density estimation. All measurements are from the medium concentration bin.

(F) Effect sizes (Cohen's d) for sequence features of large library sequences (medium concentration bin) that form condensate versus those that do not, for GFP and SNAP-tag fusions. Each dot represents one sequence feature (fractions of individual amino acids,

fraction ILMV, fraction RK, fraction DE, fraction GS, fraction YFW, fraction R/RK, fraction D/DE, fraction S/SG, fraction N/NQ, fraction Y/YF, fraction F/FW, fraction Y/YW, fraction R/RQ, fraction K/KQ, fraction FYW/FYWILV, fraction FYW/FYWR, NCPR, FCR, mean hydropathy, δ_{+-}, Ω_{+}, Ω_{-}, Ω_{FWY}, Ω_{ILMV}, and Ω_{STNQCH}). Positive effect sizes mean that values of the sequence feature are higher for sequences that form condensates. For example, the effect size (Cohen's d) for the NCPR of test protein sequences that formed condensates versus those that do not was 1.1 for GFP fusions and 0.98 for SNAP-tag fusions, suggesting that higher NCPR promotes condensate formation for both GFP and SNAP-tag fusions (there is a single point in this plot representing NCPR, plotted at x=1.1, y=0.98). On the other hand, the effect size (Cohen's d) for the fraction of negatively charged amino acids ("fraction DE") of test protein sequences that formed condensates versus those that do not was -0.46 for GFP fusions and -0.59 for SNAP-tag fusions, suggesting that lower fractions of negatively charged amino acids promote condensate formation for both GFP and SNAP-tag fusions (there is a single point in this plot representing "fraction DE", plotted at x = -0.46, y = -0.59). Pearson's r value for GFP versus SNAP-tag fusion effect sizes is shown on the plot.

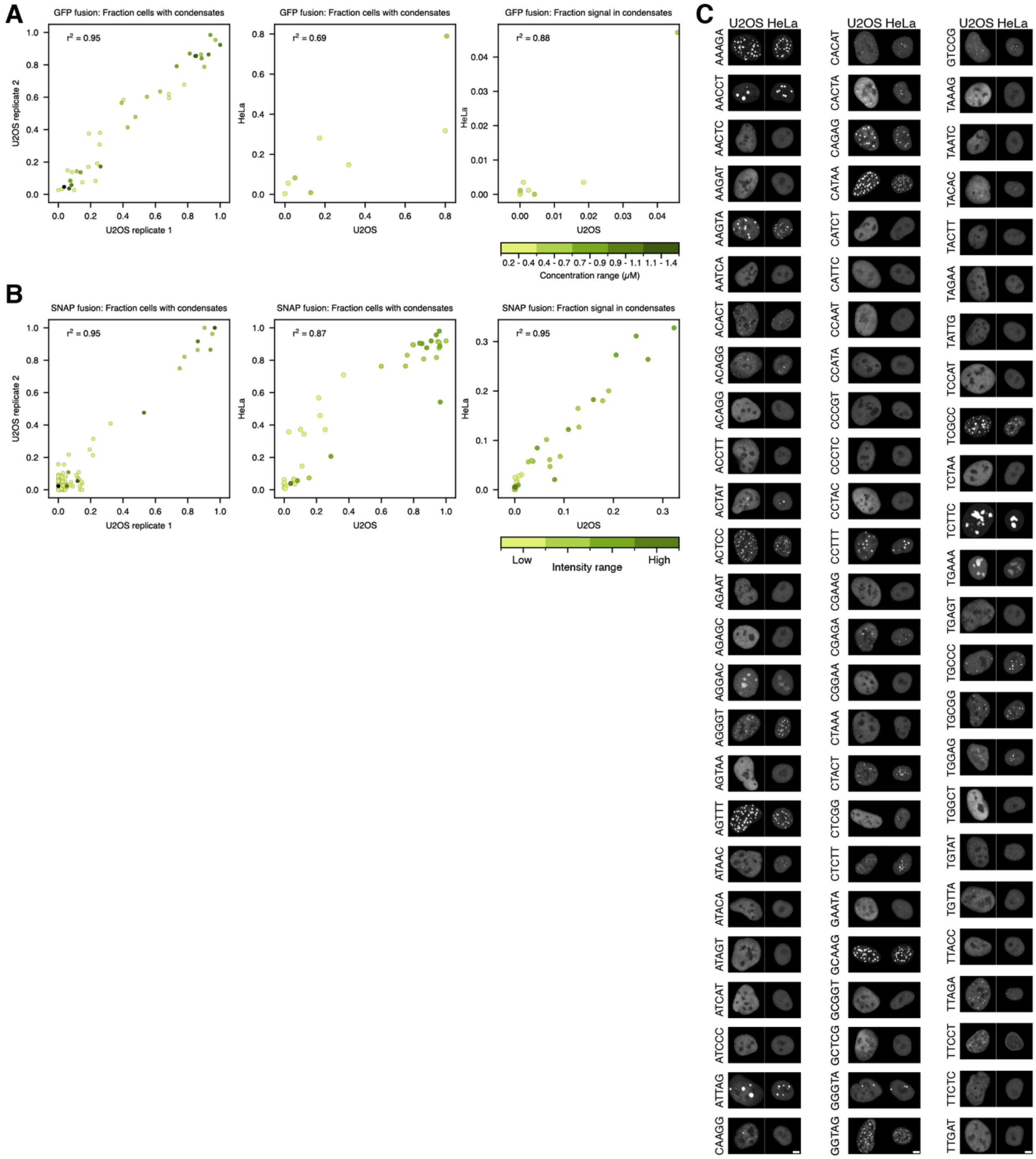

Supplementary Figure 8. CondenSeq experiments in U2OS cells.
(A and B) Comparison between condensate formation in HeLa versus U2OS cells for (A) GFP fusions and (B) SNAP-tag fusions. (Left) Reproducibility of U2OS data; each point represents one protein sequence. (Middle) The fraction of cells with condensates and (Right) the mean fraction of the fluorescent protein signal in condensates for test proteins expressed in HeLa versus U2OS cells. Note that values for protein sequences are only compared between replicates or between cell lines when they are expressed at the same level (in the same concentration bin) in each replicate or in each cell line.

(C) Representative images of test proteins expressed in HeLa and U2OS nuclei (masked). Barcodes are indicated on the left of each pair of images. All images shown are for GFP fusions. Scale bars denote 5 μm. Two independent biological replicates of this experiment were performed with the same results.

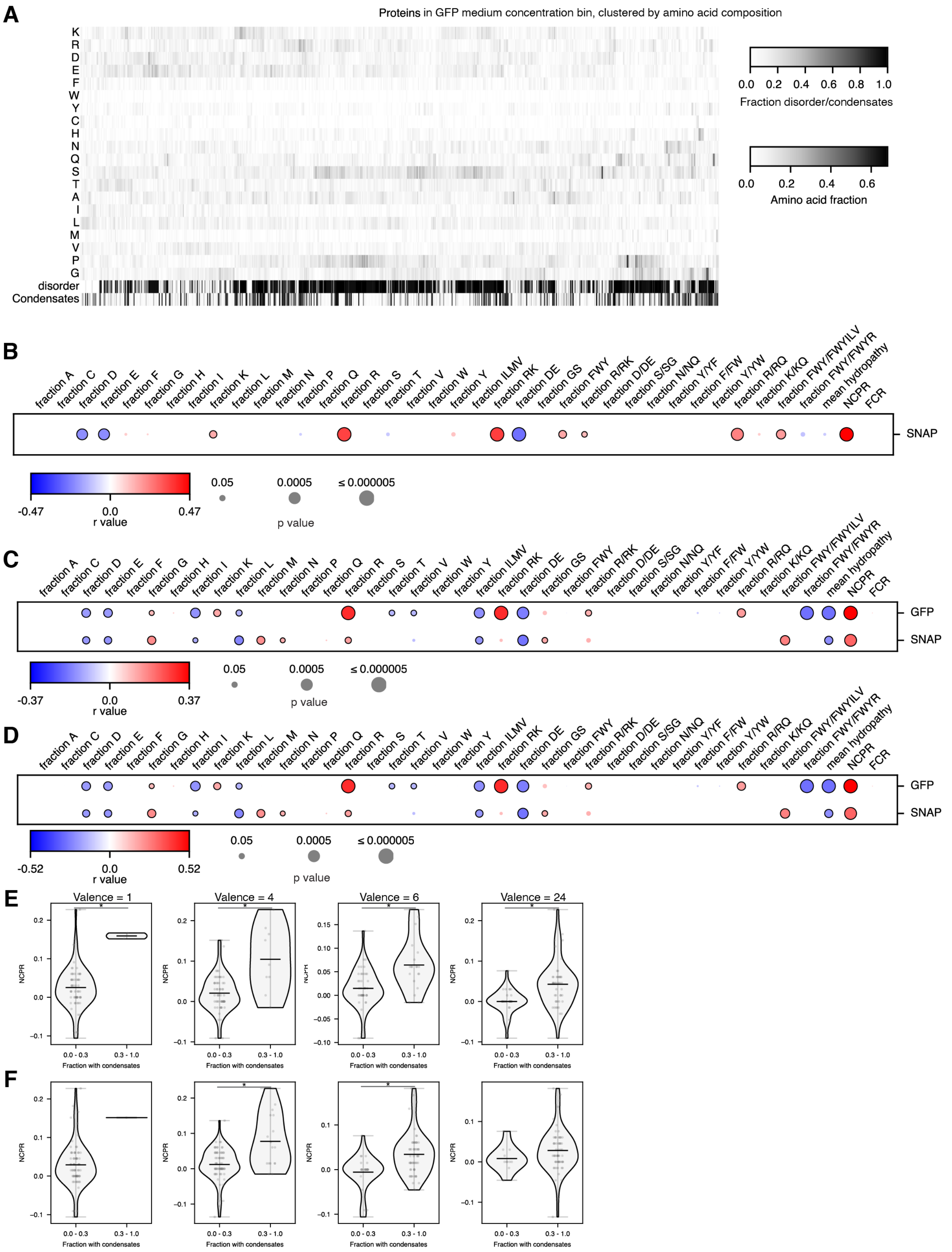

Supplementary Figure 9. Characterizing and validating trends in the natural protein sequence fragment set.

(A) Sequences from the natural protein sequence fragment library within the medium GFP concentration bin, clustered by amino acid composition. Each column represents one protein sequence. Single letters indicate specific amino acids. Grayscale indicates the fraction of the total sequence composed of a particular amino acid, the fraction of the sequence that is predicted to be disordered, or the fraction of cells expressing the sequence that contain condensates within the medium concentration bin.
(B, C, and D) Correlation between sequence features in the diverse library of protein sequences and $f_{condensates}$ for the medium (B, SNAP-tag fusions; see **Fig. 2C** for GFP fusions), low (C), and high (D) concentration bins. The colors of the dots represent the Pearson correlation (r value), and the sizes of the dots indicate the associated p value. Dots for sequence features with p values of less than 0.05 are outlined in black. p values are adjusted for multiple comparisons by applying the Bonferroni correction.
(E and F) The NCPR for protein sequences in the small sequence library that do or do not form condensates for four different oligomerization domains for GFP (E) and SNAP-tag fusions (F). Each point represents a single protein sequence. Differences in NCPR values for sequences that form condensates ($f_{condensates} \geq 0.3$) vs. those that do not form condensates ($f_{condensates} < 0.3$) assessed with a two-sided t-test for $f_{condensates} < 0.3$ vs ≥ 0.3; GFP p-values: 1mer=0.0008, 4mer=1×10^{-5}, 6mer=0.0001, 24mer=0.0003; SNAP-tag p-values: 4mer=9×10^{-6}, 6mer=0.0003, 24mer=0.23. Additionally, there is a positive correlation between NCPR and $f_{condensates}$ with Pearson correlation coefficients ranging from 0.42 to 0.63. GFP 1mer $f_{condensates} < 0.3$ n=64 sequences; GFP 1mer $f_{condensates} \geq 0.3$ n=2 sequences; GFP 4mer $f_{condensates} < 0.3$ n=63 sequences; GFP 4mer $f_{condensates} \geq 0.3$ n=9 sequences; GFP 6mer $f_{condensates} < 0.3$ n=44 sequences; GFP 6mer $f_{condensates} \geq 0.3$ n=20 sequences; GFP 24mer $f_{condensates} < 0.3$ n=31 sequences; GFP 24mer $f_{condensates} \geq 0.3$ n=47 sequences; SNAP 1mer $f_{condensates} < 0.3$ n=74 sequences; SNAP 1mer $f_{condensates} \geq 0.3$ n=1 sequences; SNAP 4mer $f_{condensates} < 0.3$ n=72 sequences; SNAP 4mer $f_{condensates} \geq 0.3$ n=17 sequences; SNAP 6mer $f_{condensates} < 0.3$ n=27 sequences; SNAP 6mer $f_{condensates} \geq 0.3$ n=59 sequences; SNAP 24mer $f_{condensates} < 0.3$ n=11 sequences; SNAP 24mer $f_{condensates} \geq 0.3$ n=63 sequences.

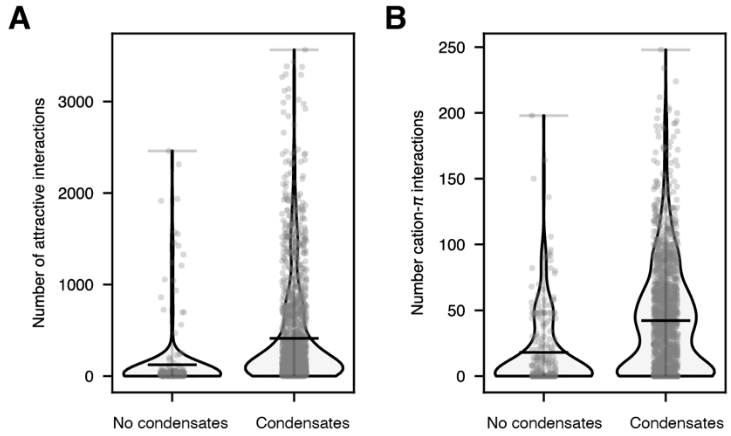

Supplementary Figure 10. Analysis of condensate-forming proteins with high NCPR.
(A) The number of possible predicted attractive interactions ($\varepsilon < -3$) between the large sequence library test proteins with NCPR > 0.05 that do or do not form condensates (GFP fusions, medium concentration bin) and all endogenous human IDRs longer than 100 amino acids (4,057 sequences). Each dot represents one protein sequence. No condensates: n=259 sequences; condensates: n=1703 sequences. The difference between the two groups is statistically significant (two-sided t-test, $p = 4\times10^{-29}$).
(B) The number of possible predicted attractive cation-π interactions (interaction parameter $\varepsilon < 0$), calculated from FINCHES homotypic intermaps (**Supplementary Note 1**), for large library test protein sequences with NCPR > 0.05 that do or do not form condensates (GFP fusions, medium concentration bin). Each dot represents one protein sequence. No condensates: n=259 sequences; condensates: n=1703 sequences. The difference between the two groups is statistically significant (two-sided t-test, $p = 5\times10^{-19}$).

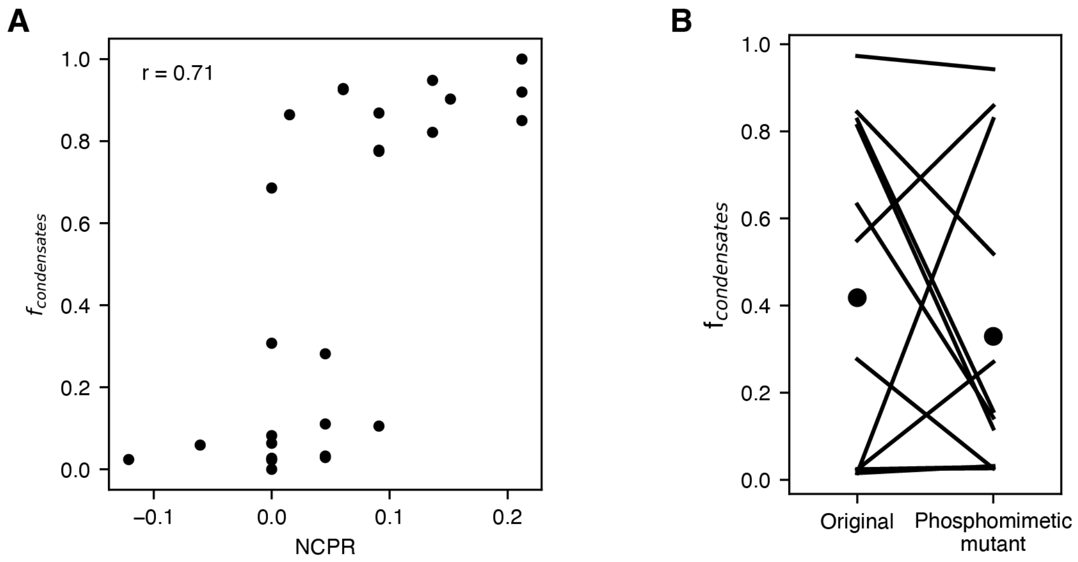

Supplementary Figure 11. Assessing potential impacts of phosphorylation.
(A) $f_{condensates}$ versus NCPR for test protein sequences that do not contain commonly phosphorylatable residues (serine, threonine, and tyrosine). Each point represents one protein sequence. Pearson's r value is shown on the plot.
(B) $f_{condensates}$ for test protein sequences ("Original") and phosphomimetic mutants (S or Y mutated to E or D). Lines connect the phosphomimetic mutants with the corresponding original sequences. Dots represent the mean $f_{condensates}$ values for the original and phosphomimetic sequences. The difference between the original and phosphomimetic sequences is not statistically significant (two-sided paired t-test, $p>0.05$).

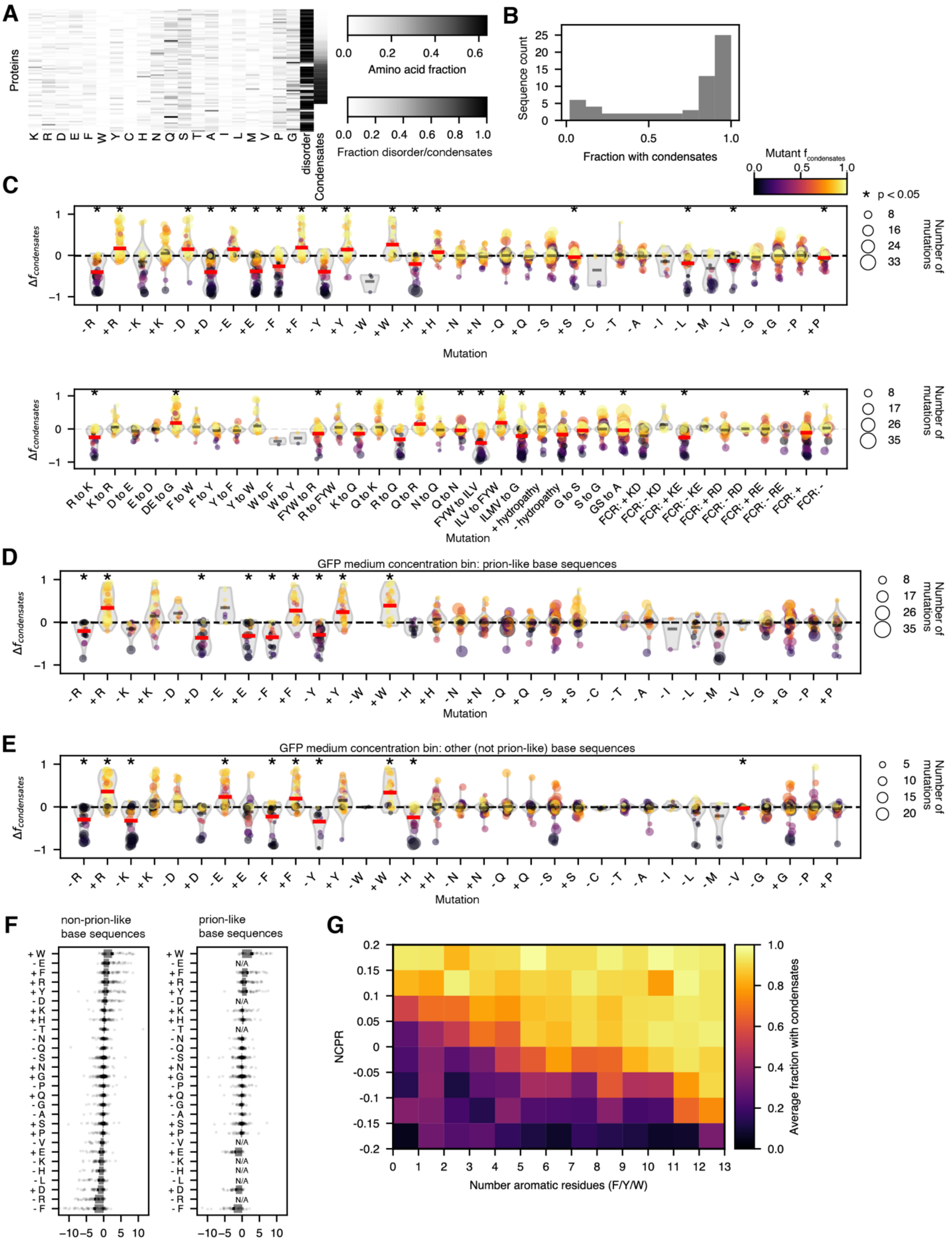

Supplementary Figure 12. Characterizing the effects of amino acid compositional variation on condensate formation.

(A) Base sequence amino acid composition, predicted disorder, and propensity to form condensates. Single letters indicate specific amino acids. Colors indicate the fraction of the total sequence composed of a particular amino acid, or the fraction of the sequence that is predicted to be disordered or the fraction of cells expressing the sequence that contain condensates within the medium concentration bin. Sequences are sorted by the fraction of cells with condensates.
(B) Histogram of the fractions of cells with condensates for the base sequences.
(C) The change in the fraction of cells with condensates for mutant sequences versus base sequences for SNAP-tag fusions. Plotted as in **Fig. 3C**.
(D and E) The change in the fraction of cells with condensates for mutant sequences relative to base sequences, as plotted in **Fig. 3C**, for prion-like base sequences (D) and non-prion-like base sequences (E) in the GFP medium concentration bin.
(F) The normalized average change in the fraction of cells with condensates for single amino acid mutations (**Methods**), for non-prion-like (left) and prion-like base sequences (right) in the medium concentration bin. Values are only computed and plotted for mutation types with data for at least 10 different base sequences for both GFP and SNAP-tag fusions. Values for prion-like base sequences are plotted in the same order as for the non-prion-like base sequences. White bars indicate values that were not computed for the non-prion-like base sequences. Values for individual sequences are overlaid as dots. Error bars represent the standard deviation of the mean.
(G) All SNAP-tag fusion sequences from the natural protein sequence fragment and compositional variation libraries binned by NCPR and number of aromatic residues; colors represent the average values of the fraction of cells with condensates over all sequences in each bin.
(C-F) Exact p-values and n values (number of sequences) provided in **Supplementary Table 9**.

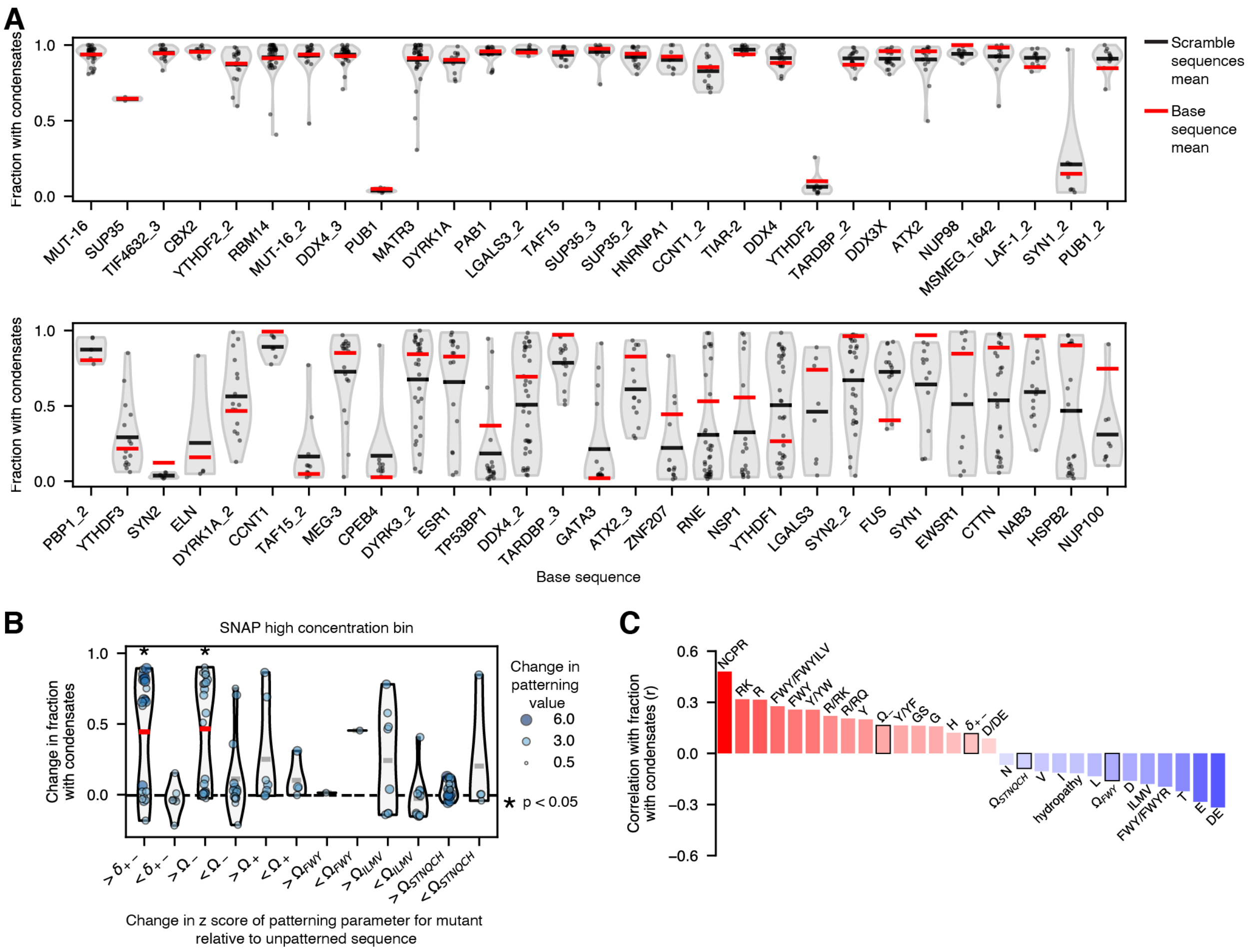

Supplementary Figure 13. Determining the effects of amino acid patterning on condensate formation for SNAP-tag fusion sequences.

(A) Each violin shows the fractions of cells with condensates for all scrambled versions of a base sequence for the SNAP-tag medium concentration bin. Red bars denote the mean values for the base sequences. Black bars denote the means of the scrambled sequences. Each black dot represents a single scrambled protein sequence. Violins are ordered by the difference between the means of the base and scrambled sequences (low to high). * denotes base sequence values (red bars) that are statistically unlikely, given the given the distribution of $f_{condensates}$ values for all of the scrambled variants of that base sequence (black dots) ($p < 0.01$, smoothed empirical CDF test, see **Supplementary Note 1**). (There are no *s present in this panel, indicating that there are no base sequence values that are statistically unlikely.)

(B) The change in the fraction of cells with condensates for patterning mutants versus unpatterned sequences, for the SNAP-tag high concentration bin, plotted as in **Extended Data Fig. 4C**. Asterisks denote groups with a significant change in the fraction of cells with condensates and red lines show their mean values (two-sided Wilcoxon signed-rank test; $>\delta_{+-}$ p-value = 0.02, $>\Omega_{-}$ p-value = 0.0004). Gray lines denote the mean values for other groups. p values are adjusted for multiple comparisons by applying the Bonferroni correction. The dashed black line is shown as a reference point marking a change of 0, i.e. no difference between mutant and base sequences.

(C) The correlation between sequence features and the fraction of cells with condensates for all sequences from the large sequence library that contain at least 5% positively charged, negatively charged, aromatic, hydrophobic, and polar amino acids (so that all patterning parameters can be computed for all sequences) in the SNAP-tag medium concentration bin. Colors indicate the Pearson correlation. Bars are only shown if p values are less than 0.05. Black borders denote the patterning parameters.

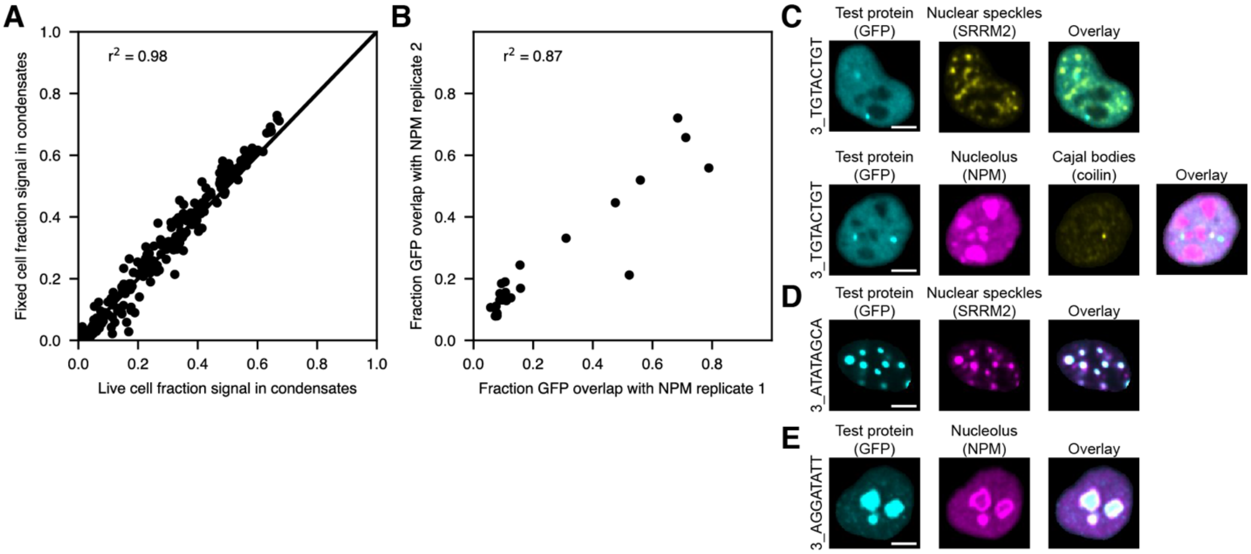

Supplementary Figure 14. Assessing colocalization with endogenous condensates.
(A) The fraction of the fluorescent protein (GFP or SNAP-tag) signal in condensates for live cell images and subsequent fixed cells images. Each point represents a single protein sequence fused to either GFP or SNAP-tag.
(B) Reproducibility for the fraction of the GFP condensates that overlap with NPM (nucleolus marker) between independent biological replicates. r^2 values for overlap with endogenous condensates ranged from 0.78 to 0.92.
(C, D, E) Representative example images of nuclei (masked) expressing test proteins that do not co-localize with SRRM2, NPM, or coilin (C) or that co-localize with SRRM2 (D), or NPM (E). All images are of fixed cells. Scale bars denote 5 μm. Two independent biological replicates of this experiment were performed with the same results.

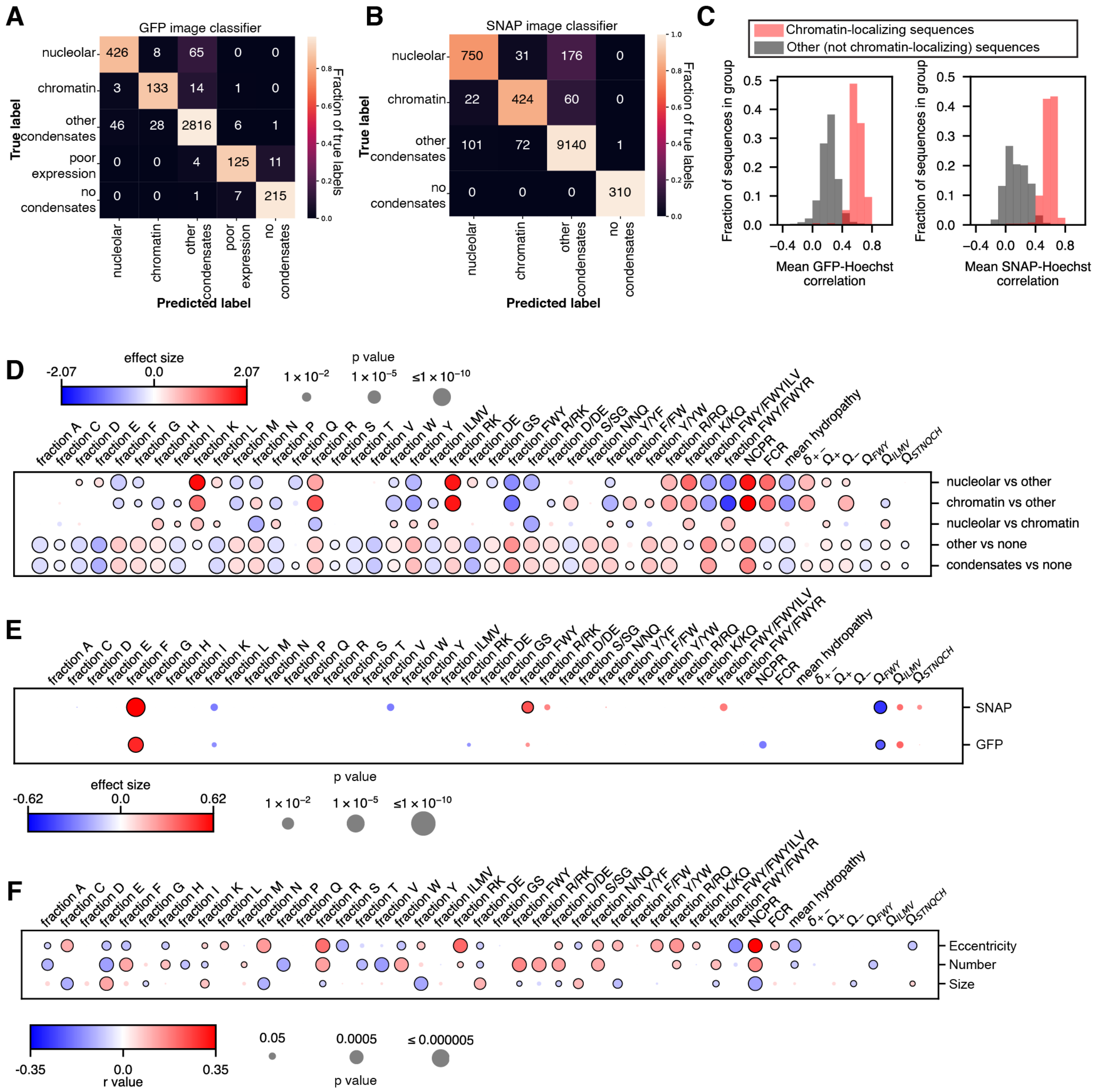

Supplementary Figure 15. Classifying different types of condensates.

(A and B) Confusion matrices for the nucleolus- and chromatin-localization classifiers for GFP (A) and SNAP-tag (B) sequences, calculated with 5-fold cross-validation on manually labeled images (**Supplementary Note 1**).

(C) Mean correlation between the GFP or SNAP-tag signal and the Hoechst signal over all images of cells expressing a given test protein sequence for sequences classified as chromatin-localizing (red) or not (gray).

(D) Comparisons of sequence features for test protein sequences classified as nucleolus-localizing, chromatin-localizing, forming other condensates, or not forming condensates for SNAP-tag sequences in the medium concentration bin. Groups are defined as in **Fig. 4C**. The colors of the dots denote the effect sizes (Cohen's d) for the comparisons between the groups indicated. Positive effect sizes (red) mean that the value of the

sequence feature tends to be higher in the first listed group as compared to the second listed group.

(E) Comparisons of sequence features for sequences exhibiting constant saturation concentration versus other sequences for GFP and SNAP-tag fusions. The colors of the dots denote the effect sizes (Cohen’s d) for the comparisons between the groups indicated. Positive effect sizes (red) mean that the value of the sequence feature tends to be higher in the constant saturation concentration sequences compared to the other sequences.

(F) Correlation between sequence features for test protein sequences in the large sequence library (GFP fusions, medium concentration bin) and average condensate eccentricity, average number of condensates per cell, and average condensate size.

(D, E, F) The sizes of the dots indicate the p values (two-sided t-test), corrected for multiple comparisons by applying the Bonferroni correction. Dots for sequence features with p values of less than 0.05 are outlined in black.

Supplementary Table 1. Experimental approaches that have been used to measure the propensities of protein variants to form condensates in cells, as well as the properties of those condensates*

	Approach	Number of protein variants per sample **	Measured in live cells?	Measures condensate morphology?	Single cell resolution?	Comprehensive, unbiased measurement of condensate composition?	Measures molecular diffusion?	Measures internal condensate architecture?	Representative references
	Electron microscopy	1	No	Yes	Yes	No	No	Yes	[55, 56]
	Cryo-electron tomography	1	No	Yes	Yes	No	No	Yes	[57]
Mass spectrometry-based	Affinity purification and mass spectrometry	1	No	No	No	Yes	No	No	[58, 59]
	Cell fractionation and mass spectrometry	1	No	No	No	Yes	No	No	[60, 61]
	Proximity labeling and mass spectrometry	1	Yes	No	No	Yes	No	No	[27, 28]
Microscopy-based	Microrheology	1	Yes	Yes	Yes	No	Yes	No	[62, 63]
	FRAP	1	Yes	Yes	Yes	No	Yes	No	[8, 64, 65]
	Single particle tracking	1	Yes	Yes	Yes	No	Yes	No	[66, 67]
	Fluorescence correlation spectroscopy	1	Yes	Yes	Yes	No	Yes	No	[68, 69]
	Super-resolution microscopy	1	Live or fixed	Yes	Yes	No	No	Yes	[59, 70]
	Arrayed immunofluorescence imaging	1	No	Yes	Yes	No	No	No	[71-73]
	Arrayed live-cell fluorescent imaging	1	Yes	Yes	Yes	No	No	No	[6, 8, 12, 25, 71]
	CondenSeq	~5000	Yes	Yes	Yes	No	No	No	This work

* Refer to recent reviews for a more in-depth analysis of approaches: [74, 75]

** The number of protein variants that can be assayed per sample, where each sequence variant is measured individually (i.e., multiple sequences of interest are not co-expressed in the same cell; if multiple sequences are co-expressed in the same cell, they may influence/interact with each other, which would not yield a sequence-specific measurement).

Supplementary Table 2. NCPR values for sequences that form condensates versus those that do not.

Sequences	Mean NCPR, test protein	Mean NCPR, full protein (including fluorescent protein and oligomerization domain)
Natural protein sequence fragment library, GFP, $f_{condensates} < 0.3$	-0.041	-0.031
Natural protein sequence fragment library, GFP, $f_{condensates} >= 0.3$	0.035	-0.022
Natural protein sequence fragment library, SNAP, $f_{condensates} < 0.3$	-0.054	-0.033
Natural protein sequence fragment library, SNAP, $f_{condensates} >= 0.3$	0.017	-0.023
Small library, GFP, valence=1, $f_{condensates} < 0.3$	0.025	-0.0040
Small library, GFP, valence=1, $f_{condensates} >= 0.3$	0.159	0.023
Small library, GFP, valence=4, $f_{condensates} < 0.3$	0.021	-0.0017
Small library, GFP, valence=4, $f_{condensates} >= 0.3$	0.104	0.013
Small library, GFP, valence=6, $f_{condensates} < 0.3$	0.015	-0.011
Small library, GFP, valence=6, $f_{condensates} >= 0.3$	0.064	-0.0020
Small library, GFP, valence=24, $f_{condensates} < 0.3$	0.000	-0.026
Small library, GFP, valence=24, $f_{condensates} >= 0.3$	0.043	-0.021
Small library, SNAP, valence=1, $f_{condensates} < 0.3$	0.029	0.0034
Small library, SNAP, valence=1, $f_{condensates} >= 0.3$	0.152	0.033
Small library, SNAP, valence=4, $f_{condensates} < 0.3$	0.012	0.0025
Small library, SNAP, valence=4, $f_{condensates} >= 0.3$	0.078	0.016
Small library, SNAP, valence=6, $f_{condensates} < 0.3$	-0.0056	-0.011
Small library, SNAP, valence=6, $f_{condensates} >= 0.3$	0.034	-0.0023
Small library, SNAP, valence=24, $f_{condensates} < 0.3$	0.0083	-0.024
Small library, SNAP, valence=24, $f_{condensates} >= 0.3$	0.028	-0.021

Supplementary Table 3. p-values and n values for **Fig. 3**.
(separate file)

Supplementary Table 4. Consistency scores for CondenSeq measurements and previously reported observations for all mutation types. Scores are computed with data from the GFP fusion medium concentration bin. The most common effects from the CondenSeq experiments were classified as disrupting condensate formation ("DISRUPT") if the mutations tended to reduce $f_{condensates}$, promoting condensate formation ("PROMOTE") if the mutations tended to increase $f_{condensates}$, or causing no change to condensate formation ("NOCHANGE") if the mutations tended to have little effect on $f_{condensates}$ (see **Supplementary Note 1**). The predicted effects of the mutation types based on previous observations were grouped in the same way, with an additional category "UNCLEAR", denoting mutation types for which their effect on condensate formation was unclear based on previous observations. The consistency scores are colored with shades of green, ranging from the darkest green color for the highest consistency score (1.0) to white for the lowest consistency score (0.53).

**Mutation type**	**Previous observations**	**References**	**Predicted effect of mutation type based on previous observations**	**Most common effect (CondenSeq)**	**Consistency score**
- R	R promotes condensate formation (can act as a "sticker," depending on the sequence context); Total charge far from 0 disrupts condensate formation *in vitro*; Net positive charge can promote condensate formation in cells	[11, 15-17]	DISRUPT	DISRUPT	0.92
- H	H can promote condensate formation	[76, 77]	DISRUPT	DISRUPT	0.89
FYW to ILV	See notes for hydropathy and aromatic residues; Higher aromatic:aliphatic ratio can lower saturation concentration	[20]	DISRUPT	DISRUPT	0.87
+ E	Adding negatively charged residues can decrease condensate formation; Total charge far from 0 disrupts condensate formation *in vitro*; Net positive charge can promote condensate formation in cells	[13, 15, 18-20]	DISRUPT	DISRUPT	0.84
- F	F promotes condensate formation (can act as a "sticker")	[11, 13]	DISRUPT	DISRUPT	0.83
- Y	Y promotes condensate formation (can act as a "sticker")	[11-13]	DISRUPT	DISRUPT	0.82
- K	Total charge far from 0 disrupts condensate formation *in vitro*; Net positive charge can promote condensate formation in cells; K weakens condensate formation *in vitro*	[11, 13, 15, 18-20]	DISRUPT	DISRUPT	0.81

FCR: + KD	High FCR may disrupt condensate formation (promotes solvation of IDRs); see also notes for K and D	2, 78, 79	DISRUPT	DISRUPT	0.80
+ D	Adding negatively charged residues can decrease condensate formation, but removing D can also decrease condensate formation; Total charge far from 0 disrupts condensate formation *in vitro*; Net positive charge can promote condensate formation in cells	11, 13, 15, 18-20	DISRUPT	DISRUPT	0.76
R to Q	See notes for R and Q		DISRUPT	DISRUPT	0.76
Low δ_{+-}	Patterning of charged residues can promote condensate formation	10, 12, 13, 15, 21, 22, 80-85	DISRUPT	DISRUPT	0.71
Low Ω_{RK}	Patterning of charged residues can promote condensate formation	10, 12, 13, 15, 21, 22, 80-85	DISRUPT	DISRUPT	0.69
High Ω_{ILMV}	Patterning of hydrophobic residues can promote condensate formation	21, 86, 87	PROMOTE	DISRUPT	0.66
- V	Hydrophobic residues can promote/stabilize condensate formation, can also affect material properties	13, 18, 19, 23, 24	DISRUPT	NOCHANGE	1.00
E to D	D versus E can change intracellular localization/interactions; E may more strongly promote condensate formation	15	DISRUPT	NOCHANGE	1.00
F to Y	Y more strongly promotes condensate formation than F	11	PROMOTE	NOCHANGE	0.96
Q to N	Q and N can act as “spacers” (little impact on propensity to form condensates, but can impact other properties, e.g., fluidity); see also notes for Q	6, 11, 13	NOCHANGE unless polyQ is disrupted, then DISRUPT	NOCHANGE	0.95
- Q	Q can act as a “spacer” (little impact on propensity to form condensates, but can impact other properties, e.g., fluidity); polyQ can promote condensate formation	6, 88	NOCHANGE unless polyQ is disrupted, then DISRUPT	NOCHANGE	0.94
GS to A	G, S, and A are non-equivalent “spacers” that can affect condensate fluidity	6, 11, 21	NOCHANGE	NOCHANGE	0.94
- T	IDRs that form condensates are typically rich in polar residues	19, 21	UNCLEAR	NOCHANGE	0.93
- G	G acts as a “spacer” and affect the fluidity of condensates	6, 11, 21	NOCHANGE	NOCHANGE	0.92

+ S	S acts as a “spacer” and affect the fluidity of condensates	6, 11, 21	NOCHANGE	NOCHANGE	0.92
N to Q	Q and N can act as “spacers” (little impact on propensity to form condensates, but can impact other properties, e.g., fluidity); see notes for Q	6, 11, 13	NOCHANGE	NOCHANGE	0.92
D to E	D versus E can change intracellular localization/interactions; E may more strongly promote condensate formation	15	PROMOTE	NOCHANGE	0.91
R to FYW	See notes for R, Y, W, and F; Aromatic residues more consistently promote condensate formation than R	11	PROMOTE	NOCHANGE	0.90
- P	Proline promotes disorder and so may be important for condensate formation; Proline-rich motifs promote condensate formation	21, 88	DISRUPT if a proline-rich motif is disrupted; otherwise, UNCLEAR	NOCHANGE	0.90
G to S	G and S are non-equivalent “spacers” that can affect condensate fluidity	6, 11, 21	NOCHANGE	NOCHANGE	0.89
+ P	Proline promotes disorder and so may be important for condensate formation; Proline-rich motifs promote condensate formation	21, 88	UNCLEAR	NOCHANGE	0.87
+ N	N can act as a “spacer” (little impact on propensity to form condensates, but can impact other properties, e.g., fluidity)	6, 11, 13	NOCHANGE	NOCHANGE	0.87
+ Q	Q can act as a “spacer” (little impact on propensity to form condensates, but can impact other properties, e.g., fluidity); polyQ can promote condensate formation	6, 88	NOCHANGE	NOCHANGE	0.87
- N	N can act as a “spacer” (little impact on propensity to form condensates, but can impact other properties, e.g., fluidity)	6, 11, 13	NOCHANGE	NOCHANGE	0.87
+ G	G acts as a “spacer” and affect the fluidity of condensates	6, 11, 21	NOCHANGE	NOCHANGE	0.87
- S	S acts as a “spacer” and affect the fluidity of condensates	6, 11, 21	NOCHANGE	NOCHANGE	0.86
R to K	R more strongly promotes condensate formation than K; R	11, 12, 15	DISRUPT	NOCHANGE	0.82

	versus K can change intracellular localization/interactions				
Y to F	Y more strongly promotes condensate formation than F	[11]	DISRUPT	NOCHANGE	0.81
- A	See notes for hydrophobic residues		DISRUPT	NOCHANGE	0.79
FCR: -	High FCR may disrupt condensate formation (promotes solvation of IDRs); see also notes for R, K, D, and E	[2, 78, 79]	DISRUPT, unless removing R, then UNCLEAR (higher FCR may DISRUPT, R may PROMOTE)	NOCHANGE	0.78
K to Q	See notes for K and Q; Total charge far from 0 disrupts condensate formation *in vitro*; Net positive charge can promote condensate formation in cells	[11, 13, 15, 18-20]	DISRUPT	NOCHANGE	0.78
FCR: + KE	High FCR may disrupt condensate formation (promotes solvation of IDRs); see also notes for K and E	[2, 78, 79]	DISRUPT	NOCHANGE	0.77
Near Ω_{ED}	Patterning of charged residues can promote condensate formation	[10, 12, 13, 15, 21, 22, 80-85]	NOCHANGE	NOCHANGE	0.77
FCR: + RE	High FCR may disrupt condensate formation (promotes solvation of IDRs); see also notes for R and E	[2, 78, 79]	UNCLEAR (higher FCR may DISRUPT, R may PROMOTE)	NOCHANGE	0.76
+ H	H can promote condensate formation	[76, 77]	PROMOTE	NOCHANGE	0.75
K to R	R more strongly promotes condensate formation than K; R versus K can change intracellular localization/interactions	[11, 12, 15]	PROMOTE	NOCHANGE	0.74
FCR: +	High FCR may disrupt condensate formation (promotes solvation of IDRs); see also notes for R, K, D, and E	[2, 78, 79]	DISRUPT, unless adding R, then UNCLEAR (higher FCR may DISRUPT, R may PROMOTE)	NOCHANGE	0.73
Near δ_{+-}	Patterning of charged residues can promote condensate formation	[10, 12, 13, 15, 21, 22, 80-85]	NOCHANGE	NOCHANGE	0.73
- L	See notes for hydrophobic residues; Leucine-rich motifs can promote condensate formation	[88]	DISRUPT	NOCHANGE	0.72
Near Ω_{FWY}	Even patterning of aromatic residues promotes liquid-liquid phase separation and inhibits aggregation;	[9, 13, 89]	NOCHANGE	NOCHANGE	0.71

	stronger “stickers” can promote condensate formation				
Near Ω_{ILMV}	Patterning of hydrophobic residues can promote condensate formation	21, 86, 87	NOCHANGE	NOCHANGE	0.71
ILMV to G	See notes for hydrophobic residues and G		DISRUPT	NOCHANGE	0.70
+ hydropathy	Hydrophobic residues can promote/stabilize condensate formation, can also affect material properties	13, 18, 19, 23, 24	PROMOTE	NOCHANGE	0.69
FCR: + RD	High FCR may disrupt condensate formation (promotes solvation of IDRs)	2, 78, 79	UNCLEAR (higher FCR may DISRUPT, R may PROMOTE)	NOCHANGE	0.69
- hydropathy	Hydrophobic residues can promote/stabilize condensate formation, can also affect material properties	13, 18, 19, 23, 24	DISRUPT	NOCHANGE	0.68
- D	Adding negatively charged residues can decrease condensate formation, but removing D can also decrease condensate formation; Total charge far from 0 disrupts condensate formation *in vitro*; Net positive charge can promote condensate formation in cells	11, 13, 15, 18-20	PROMOTE	NOCHANGE	0.67
Low Ω_{STNQCH}			UNCLEAR	NOCHANGE	0.66
Low Ω_{ED}	Patterning of charged residues can promote condensate formation	10, 12, 13, 15, 21, 22, 80-85	DISRUPT	NOCHANGE	0.64
High Ω_{ED}	Patterning of charged residues can promote condensate formation	10, 12, 13, 15, 21, 22, 80-85	PROMOTE	NOCHANGE	0.64
FYW to R	See notes for R, Y, W, and F; Aromatic residues more consistently promote condensate formation than R	11	DISRUPT	NOCHANGE	0.63
High Ω_{RK}	Patterning of charged residues can promote condensate formation	10, 12, 13, 15, 21, 22, 80-85	PROMOTE	NOCHANGE	0.62
Near Ω_{STNQCH}			UNCLEAR	NOCHANGE	0.62
High Ω_{STNQCH}			UNCLEAR	NOCHANGE	0.61
High Ω_{FWY}	Even patterning of aromatic residues promotes liquid-liquid phase separation and inhibits aggregation; stronger “stickers” can promote condensate formation	9, 13, 89	UNCLEAR	NOCHANGE	0.60
Low Ω_{FWY}	Even patterning of aromatic residues promotes liquid-liquid	9, 13, 89	UNCLEAR	NOCHANGE	0.58

	phase separation and inhibits aggregation; stronger “stickers” can promote condensate formation				
Low Ω_{ILMV}	Patterning of hydrophobic residues can promote condensate formation	21, 86, 87	DISRUPT	NOCHANGE	0.56
Near Ω_{RK}	Patterning of charged residues can promote condensate formation	10, 12, 13, 15, 21, 22, 80-85	NOCHANGE	NOCHANGE	0.54
High δ_{+-}	Patterning of charged residues can promote condensate formation	10, 12, 13, 15, 21, 22, 80-85	PROMOTE	NOCHANGE	0.53
Y to W	See notes for Y and W; W makes stronger cation-π interactions with R than Y or F; W promotes condensate formation more than Y or F in prion-like domains	20, 90	PROMOTE	PROMOTE	0.95
+ W	Aromatic residues promote condensate formation (can act as a “sticker”)	11-13	PROMOTE	PROMOTE	0.93
Q to R	See notes for R and Q		PROMOTE unless polyQ is disrupted, then UNCLEAR	PROMOTE	0.91
+ R	R promotes condensate formation (can act as a “sticker,” depending on the sequence context); Total charge far from 0 disrupts condensate formation *in vitro*; Net positive charge can promote condensate formation in cells	11, 15-17	PROMOTE	PROMOTE	0.91
ILV to FYW	See notes for hydropathy and aromatic residues; Higher aromatic:aliphatic ratio can lower saturation concentration	20	PROMOTE	PROMOTE	0.90
Q to K	See notes for K and Q; Total charge far from 0 disrupts condensate formation *in vitro*; Net positive charge can promote condensate formation in cells	11, 13, 15, 18-20	PROMOTE unless polyQ is disrupted, then UNCLEAR	PROMOTE	0.86
+ F	F promotes condensate formation (can act as a “sticker”)	11, 13	PROMOTE	PROMOTE	0.82
+ Y	Y promotes condensate formation (can act as a “sticker”)	11-13	PROMOTE	PROMOTE	0.82
F to W	See notes for F and W; W makes stronger cation-π interactions with R than Y or F; W promotes condensate formation more than Y	20, 90	PROMOTE	PROMOTE	0.81

	or F in prion-like domains				
DE to G	Adding negatively charged residues can decrease condensate formation; Total charge far from 0 disrupts condensate formation *in vitro*; Net positive charge can promote condensate formation in cells	[11, 13, 15, 18-20]	PROMOTE	PROMOTE	0.76
+ K	Total charge far from 0 disrupts condensate formation *in vitro*; Net positive charge can promote condensate formation in cells; K weakens condensate formation *in vitro*	[11, 13, 15, 18-20]	PROMOTE	PROMOTE	0.71
- E	Adding negatively charged residues can decrease condensate formation; Total charge far from 0 disrupts condensate formation *in vitro*; Net positive charge can promote condensate formation in cells	[13, 15, 18-20]	PROMOTE	PROMOTE	0.71

Supplementary Table 5. Co-localization of GFP and SNAP-tag sequences with endogenous nuclear condensates.
(separate file)

Supplementary Table 6. Number of condensate-forming, nucleolar, and chromatin-associated sequences.

**Library/concentration range**	**Total number of protein sequences**	**Number condensate forming sequences**	**Number nucleolar sequences**	**Number chromatin sequences**
GFP low concentration	8671	3899	396	93
GFP medium concentration	6994	3837	622	176
GFP high concentration	5502	3001	666	205
SNAP low concentration	6126	4497	63	10
SNAP medium concentration	7824	5804	359	254
SNAP high concentration	9442	6185	924	353

Supplementary Table 7. Effects of mutating positively charged residues on nucleolar and chromatin localization. Localization is classified as nucleolar, chromatin, other (the sequence forms condensates, but does not localize to the nucleolus or chromatin), or none (the sequence does not form condensates). All data is shown for GFP fusions in the medium concentration bin.
(separate file)

Supplementary Table 8. Primer sequences and DNA sequences encoding constructs for arrayed experiments.
(separate file)

Supplementary Table 9. p-values and n values for **Supplementary Figure 12**.
(separate file)

Supplementary Data 1. Data for small sequence library.
(separate file)

Supplementary Data 2. Long sequence library information and data.
(separate file)

Supplementary Data 3. Data for large sequence library.
(separate file)

References

1. Bracha, D., et al., *Mapping local and global liquid phase behavior in living cells using photo-oligomerizable seeds.* Cell, 2018. **175**(6): p. 1467-1480. e13.
2. Borcherds, W., A. Bremer, M.B. Borgia, and T. Mittag, *How do intrinsically disordered protein regions encode a driving force for liquid-liquid phase separation?* Curr Opin Struct Biol, 2021. **67**: p. 41-50.
3. Banani, S.F., H.O. Lee, A.A. Hyman, and M.K. Rosen, *Biomolecular condensates: organizers of cellular biochemistry.* Nat Rev Mol Cell Biol, 2017. **18**(5): p. 285-298.
4. Banani, S.F., et al., *Compositional Control of Phase-Separated Cellular Bodies.* Cell, 2016. **166**(3): p. 651-663.
5. Ginell, G.M., et al., *Direct prediction of intermolecular interactions driven by disordered regions.* bioRxiv, 2024: p. 2024.06.03.597104.
6. Wang, J., et al., *A Molecular Grammar Governing the Driving Forces for Phase Separation of Prion-like RNA Binding Proteins.* Cell, 2018. **174**(3): p. 688-699 e16.
7. Cohan, M.C., M.K. Shinn, J.M. Lalmansingh, and R.V. Pappu, *Uncovering non-random binary patterns within sequences of intrinsically disordered proteins.* Journal of molecular biology, 2022. **434**(2): p. 167373.
8. Nott, T.J., et al., *Phase transition of a disordered nuage protein generates environmentally responsive membraneless organelles.* Mol Cell, 2015. **57**(5): p. 936-947.
9. Martin, E.W., et al., *Valence and patterning of aromatic residues determine the phase behavior of prion-like domains.* Science, 2020. **367**(6478): p. 694-699.
10. Pak, C.W., et al., *Sequence Determinants of Intracellular Phase Separation by Complex Coacervation of a Disordered Protein.* Mol Cell, 2016. **63**(1): p. 72-85.
11. Bremer, A., et al., *Deciphering how naturally occurring sequence features impact the phase behaviours of disordered prion-like domains.* Nat Chem, 2022. **14**(2): p. 196-207.
12. Schuster, B.S., et al., *Identifying sequence perturbations to an intrinsically disordered protein that determine its phase-separation behavior.* Proc Natl Acad Sci U S A, 2020. **117**(21): p. 11421-11431.
13. Martin, E.W. and T. Mittag, *Relationship of Sequence and Phase Separation in Protein Low-Complexity Regions.* Biochemistry, 2018. **57**(17): p. 2478-2487.
14. Lin, Y., S.L. Currie, and M.K. Rosen, *Intrinsically disordered sequences enable modulation of protein phase separation through distributed tyrosine motifs.* Journal of Biological Chemistry, 2017. **292**(46): p. 19110-19120.
15. Greig, J.A., et al., *Arginine-enriched mixed-charge domains provide cohesion for nuclear speckle condensation.* Molecular cell, 2020. **77**(6): p. 1237-1250. e4.
16. Kaur, T., et al., *Sequence-encoded and composition-dependent protein-RNA interactions control multiphasic condensate morphologies.* Nature communications, 2021. **12**(1): p. 872.
17. Quiroz, F.G., et al., *Liquid-liquid phase separation drives skin barrier formation.* Science, 2020. **367**(6483): p. eaax9554.

18. Quiroz, F.G. and A. Chilkoti, *Sequence heuristics to encode phase behaviour in intrinsically disordered protein polymers.* Nature materials, 2015. **14**(11): p. 1164-1171.
19. Yang, Y., H.B. Jones, T.P. Dao, and C.A. Castañeda, *Single amino acid substitutions in stickers, but not spacers, substantially alter UBQLN2 phase transitions and dense phase material properties.* The Journal of Physical Chemistry B, 2019. **123**(17): p. 3618-3629.
20. Dzuricky, M., et al., *De novo engineering of intracellular condensates using artificial disordered proteins.* Nature chemistry, 2020. **12**(9): p. 814-825.
21. Schuster, B.S., et al., *Biomolecular condensates: Sequence determinants of phase separation, microstructural organization, enzymatic activity, and material properties.* The journal of physical chemistry B, 2021. **125**(14): p. 3441-3451.
22. Shapiro, D.M., M. Ney, S.A. Eghtesadi, and A. Chilkoti, *Protein phase separation arising from intrinsic disorder: first-principles to bespoke applications.* The Journal of Physical Chemistry B, 2021. **125**(25): p. 6740-6759.
23. Burke, K.A., A.M. Janke, C.L. Rhine, and N.L. Fawzi, *Residue-by-residue view of in vitro FUS granules that bind the C-terminal domain of RNA polymerase II.* Molecular cell, 2015. **60**(2): p. 231-241.
24. Weber, S.C., *Sequence-encoded material properties dictate the structure and function of nuclear bodies.* Current opinion in cell biology, 2017. **46**: p. 62-71.
25. Tripathi, S., et al., *Defining the condensate landscape of fusion oncoproteins.* Nat Commun, 2023. **14**(1): p. 6008.
26. Rekhi, S., et al., *Expanding the molecular language of protein liquid-liquid phase separation.* Nat Chem, 2024. **16**(7): p. 1113-1124.
27. Youn, J.Y., et al., *High-Density Proximity Mapping Reveals the Subcellular Organization of mRNA-Associated Granules and Bodies.* Mol Cell, 2018. **69**(3): p. 517-532 e11.
28. Youn, J.Y., et al., *Properties of Stress Granule and P-Body Proteomes.* Mol Cell, 2019. **76**(2): p. 286-294.
29. Irgen-Gioro, S., S. Yoshida, V. Walling, and S. Chong, *Fixation can change the appearance of phase separation in living cells.* Elife, 2022. **11**.
30. Dörner, K., et al., *Tag with Caution — How protein tagging influences the formation of condensates.* bioRxiv, 2024: p. 2024.10.04.616694.
31. Mészáros, B., et al., *PhaSePro: the database of proteins driving liquid–liquid phase separation.* Nucleic acids research, 2020. **48**(D1): p. D360-D367.
32. Schmidt, H.B., A. Barreau, and R. Rohatgi, *Phase separation-deficient TDP43 remains functional in splicing.* Nature communications, 2019. **10**(1): p. 4890.
33. Altmeyer, M., et al., *Liquid demixing of intrinsically disordered proteins is seeded by poly (ADP-ribose).* Nature communications, 2015. **6**(1): p. 8088.
34. Saito, M., et al., *Acetylation of intrinsically disordered regions regulates phase separation.* Nature chemical biology, 2019. **15**(1): p. 51-61.
35. Andrusiak, M.G., et al., *Inhibition of axon regeneration by liquid-like TIAR-2 granules.* Neuron, 2019. **104**(2): p. 290-304. e8.
36. Piovesan, D., et al., *MobiDB: intrinsically disordered proteins in 2021.* Nucleic acids research, 2021. **49**(D1): p. D361-D367.

37. Wang, X., et al., *LLPSDB v2. 0: an updated database of proteins undergoing liquid–liquid phase separation in vitro.* Bioinformatics, 2022. **38**(7): p. 2010-2014.
38. Quaglia, F., et al., *DisProt in 2022: improved quality and accessibility of protein intrinsic disorder annotation.* Nucleic Acids Research, 2022. **50**(D1): p. D480-D487.
39. Mészáros, B., G. Erdős, and Z. Dosztányi, *IUPred2A: context-dependent prediction of protein disorder as a function of redox state and protein binding.* Nucleic acids research, 2018. **46**(W1): p. W329-W337.
40. Kyte, J. and R.F. Doolittle, *A simple method for displaying the hydropathic character of a protein.* Journal of molecular biology, 1982. **157**(1): p. 105-132.
41. Zulkower, V. and S. Rosser, *DNA Chisel, a versatile sequence optimizer.* Bioinformatics, 2020. **36**(16): p. 4508-4509.
42. Zhang, Q., et al., *Visualizing Dynamics of Cell Signaling In Vivo with a Phase Separation-Based Kinase Reporter.* Mol Cell, 2018. **69**(2): p. 334-346 e4.
43. Grigoryan, G., et al., *Computational design of virus-like protein assemblies on carbon nanotube surfaces.* Science, 2011. **332**(6033): p. 1071-6.
44. Huang, P.S., et al., *High thermodynamic stability of parametrically designed helical bundles.* Science, 2014. **346**(6208): p. 481-485.
45. Thomson, A.R., et al., *Computational design of water-soluble alpha-helical barrels.* Science, 2014. **346**(6208): p. 485-8.
46. Garcia-Jove Navarro, M., et al., *RNA is a critical element for the sizing and the composition of phase-separated RNA–protein condensates.* Nature communications, 2019. **10**(1): p. 3230.
47. Necci, M., D. Piovesan, Z. Dosztanyi, and S.C.E. Tosatto, *MobiDB-lite: fast and highly specific consensus prediction of intrinsic disorder in proteins.* Bioinformatics, 2017. **33**(9): p. 1402-1404.
48. Stringer, C., T. Wang, M. Michaelos, and M. Pachitariu, *Cellpose: a generalist algorithm for cellular segmentation.* Nature methods, 2021. **18**(1): p. 100-106.
49. Feldman, D., et al., *Pooled genetic perturbation screens with image-based phenotypes.* Nature protocols, 2022. **17**(2): p. 476-512.
50. Kobayashi, H., K.C. Cheveralls, M.D. Leonetti, and L.A. Royer, *Self-supervised deep learning encodes high-resolution features of protein subcellular localization.* Nature methods, 2022. **19**(8): p. 995-1003.
51. McInnes, L., J. Healy, and J. Melville, *Umap: Uniform manifold approximation and projection for dimension reduction.* arXiv preprint arXiv:1802.03426, 2018.
52. Pedregosa, F., et al., *Scikit-learn: Machine learning in Python.* the Journal of machine Learning research, 2011. **12**: p. 2825-2830.
53. Joseph, J.A., et al., *Physics-driven coarse-grained model for biomolecular phase separation with near-quantitative accuracy.* Nat Comput Sci, 2021. **1**(11): p. 732-743.
54. von Bülow, S., G. Tesei, and K. Lindorff-Larsen, *Prediction of phase separation propensities of disordered proteins from sequence.* bioRxiv, 2024: p. 2024.06.03.597109.
55. Scheer, U. and D. Weisenberger, *The nucleolus.* Curr Opin Cell Biol, 1994. **6**(3): p. 354-9.

56. Gall, J.G., M. Bellini, Z. Wu, and C. Murphy, *Assembly of the nuclear transcription and processing machinery: Cajal bodies (coiled bodies) and transcriptosomes.* Mol Biol Cell, 1999. **10**(12): p. 4385-402.
57. Bauerlein, F.J.B., et al., *In Situ Architecture and Cellular Interactions of PolyQ Inclusions.* Cell, 2017. **171**(1): p. 179-187 e10.
58. Ayache, J., et al., *P-body assembly requires DDX6 repression complexes rather than decay or Ataxin2/2L complexes.* Molecular Biology of the Cell, 2015. **26**(14): p. 2579-2595.
59. Jain, S., et al., *ATPase-Modulated Stress Granules Contain a Diverse Proteome and Substructure.* Cell, 2016. **164**(3): p. 487-98.
60. Villanueva, E., et al., *System-wide analysis of RNA and protein subcellular localization dynamics.* Nat Methods, 2024. **21**(1): p. 60-71.
61. Andersen, J.S., et al., *Nucleolar proteome dynamics.* Nature, 2005. **433**(7021): p. 77-83.
62. Weihs, D., T.G. Mason, and M.A. Teitell, *Bio-microrheology: a frontier in microrheology.* Biophys J, 2006. **91**(11): p. 4296-305.
63. Feric, M. and C.P. Brangwynne, *A nuclear F-actin scaffold stabilizes ribonucleoprotein droplets against gravity in large cells.* Nat Cell Biol, 2013. **15**(10): p. 1253-9.
64. Ishikawa-Ankerhold, H.C., R. Ankerhold, and G.P. Drummen, *Advanced fluorescence microscopy techniques--FRAP, FLIP, FLAP, FRET and FLIM.* Molecules, 2012. **17**(4): p. 4047-132.
65. Li, P., et al., *Phase transitions in the assembly of multivalent signalling proteins.* Nature, 2012. **483**(7389): p. 336-40.
66. Gao, G., E.R. Sumrall, and N.G. Walter, *Single molecule tracking reveals nanodomains in biomolecular condensates.* bioRxiv, 2024: p. 2024.04.01.587651.
67. Parmar, B.S. and S.C. Weber, *Single-Molecule Tracking of RNA Polymerase In and Out of Condensates in Live Bacterial Cells.* Methods Mol Biol, 2023. **2563**: p. 371-381.
68. Maharana, S., et al., *RNA buffers the phase separation behavior of prion-like RNA binding proteins.* Science, 2018. **360**(6391): p. 918-921.
69. Perego, E., et al., *Single-photon microscopy to study biomolecular condensates.* Nat Commun, 2023. **14**(1): p. 8224.
70. Lee, K.H., et al., *C9orf72 Dipeptide Repeats Impair the Assembly, Dynamics, and Function of Membrane-Less Organelles.* Cell, 2016. **167**(3): p. 774-788 e17.
71. Banani, S.F., et al., *Genetic variation associated with condensate dysregulation in disease.* Dev Cell, 2022. **57**(14): p. 1776-1788.e8.
72. Barkley, R.J.R., et al., *Fluorescent protein tags affect the condensation properties of a phase-separating viral protein.* Mol Biol Cell, 2024. **35**(7): p. ar100.
73. Kim, H.J., et al., *Mutations in prion-like domains in hnRNPA2B1 and hnRNPA1 cause multisystem proteinopathy and ALS.* Nature, 2013. **495**(7442): p. 467-73.
74. Mitrea, D.M., et al., *Methods for Physical Characterization of Phase-Separated Bodies and Membrane-less Organelles.* J Mol Biol, 2018. **430**(23): p. 4773-4805.

75. Alberti, S., A. Gladfelter, and T. Mittag, *Considerations and challenges in studying liquid-liquid phase separation and biomolecular condensates.* Cell, 2019. **176**(3): p. 419-434.
76. Wang, W., et al., *A histidine cluster determines YY1-compartmentalized coactivators and chromatin elements in phase-separated enhancer clusters.* Nucleic Acids Res, 2022. **50**(9): p. 4917-4937.
77. Lim, J., et al., *Liquid-Liquid Phase Separation of Short Histidine- and Tyrosine-Rich Peptides: Sequence Specificity and Molecular Topology.* J Phys Chem B, 2021. **125**(25): p. 6776-6790.
78. Das, R.K., K.M. Ruff, and R.V. Pappu, *Relating sequence encoded information to form and function of intrinsically disordered proteins.* Curr Opin Struct Biol, 2015. **32**: p. 102-12.
79. Martin, E.W., et al., *Sequence Determinants of the Conformational Properties of an Intrinsically Disordered Protein Prior to and upon Multisite Phosphorylation.* Journal of the American Chemical Society, 2016. **138**(47): p. 15323-15335.
80. Lin, Y.H., J.P. Brady, J.D. Forman-Kay, and H.S. Chan, *Charge pattern matching as a 'fuzzy' mode of molecular recognition for the functional phase separations of intrinsically disordered proteins.* New Journal of Physics, 2017. **19**.
81. Lin, Y.H. and H.S. Chan, *Phase Separation and Single-Chain Compactness of Charged Disordered Proteins Are Strongly Correlated.* Biophys J, 2017. **112**(10): p. 2043-2046.
82. Lin, Y.-H., J.D. Forman-Kay, and H.S. Chan, *Sequence-specific polyampholyte phase separation in membraneless organelles.* Physical review letters, 2016. **117**(17): p. 178101.
83. Das, R.K. and R.V. Pappu, *Conformations of intrinsically disordered proteins are influenced by linear sequence distributions of oppositely charged residues.* Proceedings of the National Academy of Sciences, 2013. **110**(33): p. 13392-13397.
84. Huihui, J., T. Firman, and K. Ghosh, *Modulating charge patterning and ionic strength as a strategy to induce conformational changes in intrinsically disordered proteins.* The Journal of Chemical Physics, 2018. **149**(8).
85. Pesce, F., et al., *Design of intrinsically disordered protein variants with diverse structural properties.* Sci Adv, 2024. **10**(35): p. eadm9926.
86. Zheng, W., et al., *Hydropathy patterning complements charge patterning to describe conformational preferences of disordered proteins.* The journal of physical chemistry letters, 2020. **11**(9): p. 3408-3415.
87. Statt, A., H. Casademunt, C.P. Brangwynne, and A.Z. Panagiotopoulos, *Model for disordered proteins with strongly sequence-dependent liquid phase behavior.* The Journal of chemical physics, 2020. **152**(7).
88. Darling, A.L., Y. Liu, C.J. Oldfield, and V.N. Uversky, *Intrinsically Disordered Proteome of Human Membrane-Less Organelles.* Proteomics, 2018. **18**(5-6): p. e1700193.
89. Weiner, B.G., A.G. Pyo, Y. Meir, and N.S. Wingreen, *Motif-pattern dependence of biomolecular phase separation driven by specific interactions.* PLoS computational biology, 2021. **17**(12): p. e1009748.

90. Maristany, M.J., et al., *Decoding Phase Separation of Prion-Like Domains through Data-Driven Scaling Laws*. 2024, eLife Sciences Publications, Ltd.
